# Discovery of a product’s unconscious culture code and testing with fMRI

**DOI:** 10.3389/fnbeh.2026.1737998

**Published:** 2026-03-24

**Authors:** Sabri Çimen, F. Burcu Candan Çam, Sema Kurtuluş, Ş. Ebru Toksoy, Hale Yapıcı Eser, Onur Ozyurt

**Affiliations:** 1 Department of Marketing, Faculty of Business Administration, Kocaeli University, Kocaeli, Türkiye; 2 Department of Marketing, Faculty of Business Administration, Istanbul University, İstanbul, Türkiye; 3 Trauma and Disaster Mental Health PhD Program, Health Sciences Institute, Kocaeli University, Kocaeli, Türkiye; 4 Department of Psychiatry, School of Medicine, Koç University, İstanbul, Türkiye; 5 Research Center for Translational Medicine, Koç University, İstanbul, Türkiye; 6 Graduate School of Health Sciences (GSHS) Neuroscience Program, Koç University, İstanbul, Türkiye; 7 Department of Clinical Neurosciences, Wolfson Brain Imaging Center, University of Cambridge, Cambridge, United Kingdom

**Keywords:** culture code, fMRI, migration, neuromarketing, truck, unconscious

## Abstract

**Introduction:**

In the quest to investigate the internal cognitive mechanisms underlying consumer behavior, often metaphorically termed the “black box,” studies indicate that purchasing decisions are significantly influenced by implicit cognitive processes. Conventional data collection methods that appeal to the conscious level may mislead marketing strategies. This motivates the rapid increase of research in two fields: psychology and neuroscience. This interdisciplinary research was focused on the cultural unconscious. Similar to the archetypes (the building blocks of the collective unconscious), cultural codes (the building blocks of the cultural unconscious) play a crucial role in shaping purchasing decisions. Based on this, the research question is “What is the cultural code of the selected product in the social unconscious of the target population?”

**Methods:**

This research comprises two consecutive sub-studies: in-depth interviews (Study 1), and an fMRI task (Study 2). A heavy-duty vehicle (truck) was chosen as the product, and professional truck drivers (*n* = 22 for Study 1 and *n* = 34 for Study 2) from Nortwest Anatolia, in Türkiye, were selected to represent the target population.

**Results:**

In Study 1, the unconscious cultural code associated with the truck was discovered as “migration” through in-depth interviews utilizing psychoanalytic and transactional approaches. In Study 2, an fMRI experiment was developed to test whether the “truck + migration” image would elicit brain activation patterns similar to those associated with unconscious relational encoding and retrieval, reported in the literature (fusiform gyrus, middle temporal gyrus, parahippocampus / hippocampus, precuneus, angular gyrus, anterior cingulate), and significantly different from those elicited by control images. Detecting these specific activations would provide neuroscientific support for the hypothesis that the concepts of truck and migration are relationally encoded in participants’ cultural unconscious, thereby validating the qualitative findings by in-depth interviews.

**Discussion:**

Identifying a product’s unconscious cultural code enables a more precise design of the marketing mix. By using these interdisciplinary qualitative and quantitative methods sequentially, an innovative and original method was developed, yielding both theoretical contributions in cultural code discovery and consumer neuroscience, as well as practical implications, particularly in marketing.

## 1 Introduction

To what extent do non-conscious cognitive processes modulate consumer choice beyond the boundaries of conscious rationalization? This inquiry is central to contemporary marketing research, which seeks to identify the neural mechanisms underlying decision-making—often metaphorically termed the “black box”—through objective psychological and neuroscientific methodologies. Understanding these latent processes is essential for accurately modeling consumer judgment and predicting behavior that remains inaccessible to traditional self-report measures. This research focuses on the growing need to bridge the gap between observed consumer discourse and the internal cognitive structures that govern market choices.

Studies show that purchasing decisions result from both conscious and unconscious processes, with unconscious processes exerting a significant influence on consumer judgment (Bargh, 2021; Fitzsimons et al., 2002; Özkara and Bagozzi, 2021). Consumer judgment, decision-making, and behavior largely rest on mental processes that reside outside of conscious awareness (Chartrand and Fitzsimons, 2010; Wörfel, 2021). More specifically, 95 percent of thoughts take place in our unconscious mind, and these are scattered, diverse memories, emotions, thoughts, primes and other cognitive processes that we are not aware of and cannot express (Bargh and Chartrand, 1999; Wegner, 2002; Woodside et al., 2012; Zaltman, 2003). Marketing professionals invest over $750 billion annually in traditional marketing, which is usually behavioral and subjective, relying on consumer self-reports from questionnaires and focus groups (Mashrur et al., 2023). The problem is that these traditional tools, which only capture external and explicit responses, may not be able to detect unconscious effects or accurately predict consumer behavior (Calvert and Brammer, 2012; Xu et al., 2023) due to the limits of the data processing capacity of working memory (Kalaganis et al., 2021; Özkara and Bagozzi, 2021; Sánchez-Fernández et al., 2021) or various psychological and social reasons (Casado-Aranda et al., 2023; Cowley and Anthony, 2019; Solnais et al., 2013; Shiv et al., 2005; Webb et al., 1966; Wilson, 2002; Woodside et al., 2012; Zaltman, 2003). This situation puts the marketing research results and, by extension, the marketing mix designs at risk. Various researchers have estimated that up to 95% of new brand launches fail (Rodríguez et al., 2023), and among other reasons, the aforementioned misalignment has the potential to be one of the main root causes. The limitations of self-report techniques (i.e., questionnaires or surveys) in measuring consumer response to marketing stimuli (Sánchez-Fernández et al., 2021) and the necessity to identify the unconscious processes and codings have resulted in the tendency to alternative data collection methods and more objective and accurate tools from two fields: psychology and neuroscience. Aligned with this shift, the present study focuses on both fields within a single research frame.

In the field of psychology, there is significant attention on the unconscious codes (such as archetypes and cultural codes) of the collective mindand and on their discovery (Adams, 2006; Bechter et al., 2016; Gu, 2023; Henderson, 1990; Jung, 1968, 1989; Lloyd and Woodside, 2013; McPeek, 2008; Mazurek-Łopacińska, 2016; Singer and Kimbles, 2004; Snowden, 2013; Woodside et al., 2012). Understanding the social dimensions of the unconscious is necessary because it is largely structured by social, cultural, and collective coordinates (Dajani, 2022). The information that is encoded in relation to a collective mind is psychologically amplified and prioritized in individual cognition (Shteynberg et al., 2023).

In the field of neuroscience, consumer neuroscience is an important advancement in understanding how the unconscious mind influences consumer decisions (Kalaganis et al., 2021; Kesarwani et al., 2025). This evolving field bridges consumer behavior studies and neuroscience and lies at the intersection of three disciplines: marketing, psychology, and neuroscience (Alsharif et al., 2025; Alvino et al., 2020; Cenizo, 2025; He et al., 2021; Jansson-Boyd and Bright, 2024; Javor et al., 2013; Singh et al., 2023; Plassmann et al., 2012). Neuromarketing, which is defined as the practical application of neuroscientific tools in the corporate and commercial sectors (Javor et al., 2013; Ramsøy, 2019), promising to overcome the limitations of traditional data collection tools, is supposed to fill the gap between survey results and the actual consumer behavior so it may assist brands and companies in accurately predicting future customer preferences (Mashrur et al., 2023; Pal et al., 2021). Neuroimaging techniques use brain-computer interface technology to understand customer preferences in response to marketing stimuli (Mashrur et al., 2023) and focuses on brain activations, not consumers’ conscious discourse (Zurawicki, 2010).

This research integrates the aforementioned fields of psychology (the cultural unconscious) and consumer neuroscience within a unified framework. It does so by sequentially applying methods from each discipline, thereby developing an original, novel, and innovative methodology. Furthermore, by utilizing both qualitative and quantitative research techniques in a consecutive manner, it presents a comprehensive and holistic study. The study focuses on the discovery of unconscious cultural codes of products using in-depth interview techniques, a field of psychology (Study 1), and the testing of these codes using fMRI (Functional Magnetic Resonance Imaging), a tool from neuroscience (Study 2). In the first stage, psychoanalytic and transactional analysis-based approaches, one of the exploratory and qualitative research methods, were employed, specifically the in-depth interview technique. The purpose of choosing this approach is to bypass conscious rationalizations. Qualitative research is frequently used in the sciences; however, it is acknowledged that it relies on experts’ experience, opinions, and comments. In this case, margins of error in the research may naturally increase due to the researcher’s subjectivity. To enhance the power of this research, in the second phase, it was decided to utilize neuroscientific imaging techniques, specifically fMRI, a causal and quantitative research method, to test the unconscious cultural code previously claimed to have been discovered. fMRI was selected for its high spatial resolution, enabling investigation of deep-seated neural activations. As a result of this setup, the output of qualitative research—the unconscious cultural code—was used as input for quantitative research. The need to use the right stimuli, a primary concern in neuroscientific research, and the need to verify the discovered cultural code, a key requirement in qualitative research, were both addressed by employing two methods in tandem and sequentially within the same research framework. Convergent scientific results, such as completing each other’s deficiencies and a much more powerful method, have been obtained.

Since the selection of the product, the sampling process, participants, general data collection methods, and tools are common to both studies, it is more efficient to share this information in the introduction. In this research, a heavy-duty vehicle, commonly known as a truck, was selected as the product. The truck is an important product in the automotive industry. It is a product with high added value in terms of design and engineering, a complex production process that requires significant capital investment, heavy industry applications, a complex supply chain, a comprehensive marketing and sales process, and a robust after-sales structure. It plays a critical role in the construction, mining, transportation, and logistics sectors, carrying raw materials, intermediate products, and final products, and has significant economic value in distribution channels. Aiming for the correct, efficient, and accurate design of the marketing mix for this critical product, discovering the unconscious culture code can offer significant advantages. Unconscious cultural codes may differ across societies and cultures. In this study, Turkish Culture and a certain geographical region in Türkiye were chosen as the target population. As a result the research question was refined as: “What is the cultural code of the truck product in the social unconscious of the target population?”

Since the product selected for research is the heavy commercial vehicle (truck), the truck drivers, owners, and drivers, or just drivers, who are influential in the purchasing decision, were determined as the target population. For this reason, truck drivers living in Northwest Anatolia in Türkiye were chosen as the target population. In total, five different participant groups were invited to 14 different data collection sessions to support the three studies mentioned above. These sessions are summarized in a table in Supplementary Material A. The names of truck drivers were obtained from transportation cooperatives, logistics companies, and transportation departments of public institutions in İstanbul, Kocaeli, Eskişehir, and Bilecik, which are located in Northwest Anatolia. They were contacted by phone and invited to participate in the study sessions.

## 2 Study 1: discovery of the product’s unconscious cultural code

### 2.1 Conceptual basis of Study 1

To examine the concept of the unconscious culture code, it is necessary to consider cognitive processes in the brain, the unconscious, the collective unconscious, archetypes, and the cultural unconscious. To understand how individuals’ general decision-making mechanisms, and, from a marketing perspective, consumers’ purchasing decision-making mechanisms, work, it is necessary to investigate the processes the human mind or brain follows. Many psychology-based and neurology-based models explain how the human mind works. These appear in different terminologies: such as “subconscious, superconscious,” “conscious level, unconscious level,” “explicit memory, implicit memory,” “ego, superego, id,” “individual unconscious, collective unconscious, cultural unconscious,” “cortex, limbic system, the lower brain,” “rational brain, emotional brain, reptilian brain,” “System 1 and System 2 processes” (Evans, 2008; Megehee and Woodside, 2010). In this research, the effects of the “Dual Process Theory” (System 1 and System 2 processes) and the concepts of “individual unconscious that Freud gave weight to, collective unconscious that Jung emphasized, and, cultural unconscious” are used as main references. Without rejecting Freud’s basic definitions, Jung says that the unconscious has two layers. The first is the individual unconscious, which is a completely private soul realm composed of repressed desires and impulses, sub-threshold perceptions, and forgotten experiences that the ego does not know or cannot access directly (Snowden, 2013), while the second is the collective unconscious, a part of the mind common to all of us. He refers to the patterns of this collective unconscious as archetypal or mythological motifs. Archetypes are universal, primitive, and pure forms of mind, and the theory that they are inherited and a priori in people’s minds is still an academic phenomenon in terms of its results, even though it is still debated as to how this happens or why (Jung, 1968, 1989; Lloyd and Woodside, 2013; Snowden, 2013; Woodside et al., 2012).

It would not be wrong to say that the concept of the cultural unconscious is built on Jung’s concept of the collective unconscious and the archetypes. Individuals, in the simplest terms, have universal belongings and values because they are human, and they are fed by the collective consciousness and collective unconscious of all humanity. Knowingly or unknowingly, they have certain codes just because they are human. However, most of the time, their beliefs, ideas, attitudes, and behaviors are fed, influenced, and adapted by the culture they belong to. When examining human behavior, especially when creating strategies in areas such as marketing, it may not be enough to act only on universal similarities and common points. This can even lead to mistakes.

The theoretical framework of the unconscious has evolved beyond individual and universal domains to include a specialized “cultural” stratum that bridges the gap between personal repression and collective archetypes. Henderson (1990) was a pioneer in defining the cultural unconscious as a distinct dimension situated between the Freudian personal unconscious and the Jungian collective unconscious, representing the shared historical and social experiences of a specific group. This conceptualization was further refined by Singer and Kimbles (2004), who introduced “cultural complexes” as semi-autonomous patterns of the collective psyche that organize a group’s emotional life and expectations. Adams (2006) finds Henderson’s definition insufficient and claims that the cultural unconscious, which includes stereotypes and stereotypical images, is the second dimension of the collective unconscious, an addition to the archetypes and archetypal images. More recently, Gu (2023) has reconceptualized the cultural unconscious as a dynamic “unconscious structure of feelings,” describing it not as a biological inheritance but as a cultivated ideology acquired through lived experience and education. Within this paradigm, cultural codes function as the essential components of a society’s archive, consisting of historical symbols and “unconscious signifiers” that dictate the invisible motivations behind social perceptions and actions. Collectively, these perspectives suggest that cultural codes act as semiotic bridges, translating latent collective memories into the manifest behaviors and judgments observed within a given society (Gu, 2023). The universal building blocks of the collective unconscious are the archetypes; similarly, it would not be wrong to define cultural codes as the building blocks of the cultural unconscious. According to Krasnykh, the culture code is a web that culture throws over the world to classify, categorize, structure, and analyze (Tambovtsev, 2015). For example, while white represents purity in most Western countries, in India, it is the color of mourning and sorrow. This is so important that, for example, the effectiveness of communication depends on the correct determination of consumers’ cultural codes, because these structures directly affect the perception of different events and objects by assigning meaning (Mazurek-Łopacińska, 2016). The features that distinguish one culture from others are those adopted by its members. Individuals belonging to that culture attribute similar meanings to events and objects. Perception and thought patterns, attitudes, behaviors, beliefs, reactions, and decision-making mechanisms are similar. In summary, cultural elements, which can be referred to as cultural codes in their unconsciously processed forms, are created by societies with a shared culture through the historical process, preserved in specific environments, and passed down from generation to generation. There are two interesting questions here. The first is “How are these common unconscious patterns and cultural codes created?” Secondly, “How do cultures transfer these codes to future generations independently of time and place?” In the creation process of the culture code, centuries of repetitions (social institutionalization and the education of individuals with the effect of mirror neurons), sudden and significant collective traumatic events, their blending with emotions, immutability, and the modular system of cognitive mechanisms located in the minds that enable basic perceptions about the world come to the forehand (Tambovtsev, 2015). Cultural codes also live and find meaning in the environment of tales, myths, heroes, lullabies, folk songs, symbols, legends, and epics. They are transmitted from generation to generation through the repetition of these environments, rituals, language, and communication. The mechanisms that enable the formation and transmission of cultural codes appear in the following basic findings of psychology, neurology, and sociology: the importance of repetition and animation in social learning as well as in individual learning (Assmann, 2015), the effect of traumas on the formation of codes on a social scale as well as in individuals (Roesler, 2012), the influence of emotions on learning (Brown and Kulik, 1977; Talarico and Rubin, 2003), completion of learning mostly in early childhood (Gantt and Agazarian, 2011), children are under the influence of a single culture (their own culture) to a great extent in early childhood (Göka, 2008; Mlynar, 2014; Roesler, 2012; Vicedo, 2009), language and narrative (story) phenomenon (Mlynar, 2014).

It is evident that the concepts of the collective unconscious and archetype, which Jung and his successors emphasized, are increasingly used in marketing. If archetypes are first identified with human personality traits and then with brand personality traits, they can be turned into power or a key tool that can influence the purchasing behavior and decision-making process of consumers in favor of brands (Bechter et al., 2016; Lloyd and Woodside, 2013; McPeek, 2008; Woodside et al., 2012). Similarly, the cultural unconscious and the unconscious cultural codes are increasingly making their weight felt in marketing (Lloyd and Woodside, 2013; Mazurek-Łopacińska, 2016). It should be noted that out of the three types of unconsciousness that must be examined to understand how consumers make decisions, none can be said to be entirely true or entirely wrong. All three are correct, but one or the other yields a more accurate result depending on the specific details required. To answer the question “Which unconscious should the marketing researcher focus on?” it will be useful to consider the segmentation perspective. The discovery of unconscious cultural codes is similar to the work done in discovering archetypes. Some of the methods used are: drawing a picture of dreams, VNA (Visual Narrative Art), enneagram and visual art, and psychoanalysis (Megehee and Woodside, 2010; Woodside et al., 2012). Analyses for archetype (or culture code) discovery are characterized by, first, identifying the root of why and how much a symbol or theme emotionally affects its followers; second, explaining why a symbol is culturally significant; and third, ultimately explaining why a symbol is motivating. For example, it discovers how the symbol is linked to the product and how this link is useful for using the product (Maso-Fleischman, 1997). In summary, the literature provides robust findings on two main topics. First, consumer judgment, decision-making, and behavior largely rest on unconscious cognitive processes. Second, the cultural unconscious and its unconscious cultural codes have a reasonable impact on consumer behavior. This is simply driven by the need to link products or brands to the codes they encounter in their culture without being aware of their existence.

### 2.2 Materials and methods

Study 1 aims to discover the unconscious cultural code of truck drivers residing in Northwest Anatolia, which has been chosen as the target population for the truck product. A pilot study was conducted as a test prior to the main sessions, during which the research team carefully observed the course and needs of the research process, the setting, and the participants, given the nature of qualitative research. Following the pilot study, the research team decided to employ the in-depth interview technique, drawing on the psychoanalytic and transactional analysis approaches, within the scope of exploratory qualitative research. The in-depth interviews were conducted in a controlled environment to minimize external distractions, utilizing high-fidelity audio-visual equipment. Led by a licensed psychotherapist to ensure clinical depth and thematic consistency, the sessions followed a standardized, semi-structured interview guide. The questions regarding the truck were strategically ordered to transition from the conscious to the unconscious level, or from System 2 to System 1 processing. Finally, all participant responses were recorded and subsequently transcribed for analysis. These texts were analyzed using qualitative research analysis techniques. The results were converted into numerical data (Kümbetoğlu, 2008). Ultimately, the alleged unconscious cultural code was discovered. The discovered cultural code was incorporated into the hypothesis for use in neuroscientific experiments, which constitutes the second phase.

#### 2.2.1 Participants: in-depth interview

The interviews were completed within three different days, 22 people attended, all male, minimum age 28, maximum age 62, Mage = 44, SD = 10, 17 of them were born in Northwest Anatolia, 22 people work in Northwest Anatolia, 12 of them are primary school graduates, four of them are secondary school graduates, five of them are high school graduates and one of them is associate degree graduate, 22 of them are married. The average number of children is 3, minimum 0, maximum 8, SD = 2. The average seniority in the profession is 22 years, with a minimum of 5 and a maximum of 40, SD = 10. A total of 22 of them are drivers, and 14 of them are both drivers and vehicle owners. A total of 22 of them drive trucks, and 17 of them drive both trucks and tow trucks.

#### 2.2.2 Data collection

Before the data collection process, the drivers were contacted through transportation cooperatives and logistics companies and provided with preliminary information. The study was conducted with three groups on three different days. Providing detailed information about the research to the participants, reading the informed consent forms in line with the Ethics Committee Decision (Kocaeli University, KOU KAEK 2015/270, Decision Number: 12/15, Date: 01/09/2015) (see Supplementary Material B), obtaining their written consent, informing the participants about the camera recording and audio recording during the studies, filling out the demographic data collection forms (see Supplementary Material C), and then the data collection process were performed. Pilot trials were conducted with the first group. A trial was made to draw a truck picture, and interpret pictures and images, first in groups and then individually. In the next experiment, participants were asked to relax and enter a deeply relaxed state in a comfortable, dim, and musical environment, accompanied by the psychotherapist. They then answered questions about the truck as a group during the transition from a deeply relaxed state to an awake state. The research team decided not to use these methods and instead changed them, based on the common view that high efficiency could not be obtained from these trial studies. The reason for this is that the participants were skeptical of these methods due to their socio-cultural and demographic characteristics, and they were observed to be unable to fully devote themselves to the study. It was decided to continue the research with an individual and an in-depth interview of approximately 45 min each.

The design of the content and the order of the questions (see Supplementary Material C) to be directed to participants during the in-depth interview is a critical stage in qualitative research. The interview questions were designed by the Authors’ Team led by the psychotherapist who processed the interview, in accordance with the theoretical structure outlined above. Throughout the interview, psychoanalytic and transactional analysis-based approaches (Berne, 1996) were followed. The interview order is designed to proceed from System 2 to System 1 (Evans, 2008; Megehee and Woodside, 2010), starting with rational questions, then more emotion-related questions, and finally ending with questions that target discovering the childhood memories, symbols, and cultural codes. This order aligns with the principles of transactional analysis as well. The content design and question order have been targeted to ensure repeatability and comparability across participants. However, due to the nature of qualitative research, the psychotherapist who conducted the interview was able to make intentional deviations when appropriate, based on the participant’s emotions and thoughts, or on the structure and content of the answers.

### 2.3 Analysis and results

Qualitative data analysis techniques were used to analyze the research findings, particularly those of Kümbetoğlu, Fielding, Thomas, Gibbs, Robson, Hall, and May. The qualitative data analysis followed a systematic approach similar to template analysis, where a coding structure is developed iteratively to organize and interpret the data (King, 2012; Kümbetoğlu, 2008). The qualitative data analysis techniques employed in this study are as follows: As Kümbetoğlu (2008) notes, citing Fielding, ideas, concepts, and themes in qualitative analysis emerge after a recursive reading of the text. In this context, a descriptive analysis is initially conducted; this involves a definitive examination of words, expressions, linguistic nuances, dialogue structure, and the symbolic narratives and metaphors present in the interview transcripts. Aligning with this view, McLuhan (1964) asserts that structure, not content, conveys the message. Therefore, the descriptive analysis phase prioritizes the structural examination of the text over the mere literal content provided by participants. Subsequently, through systematic analysis, specific concepts and themes within the data are identified, and their relationships are defined to reach causal and explanatory conclusions. In qualitative data analysis, the entire dataset should first be reduced into analytical categories. The process of establishing these analytical categories is defined as coding. Concepts and categories are decomposed into codes that serve as a guide for researchers navigating the vast array of qualitative data. Each code serves as a symbol for classifying a group of words. These codes and signs are organizational markers that allow the researchers to aggregate specific types of examples and locate them within the text. Codes constitute the structure of the text and denote a particular idea, subject, or concept within the relevant segment (Kümbetoğlu, 2008).

To arrive at a theory based on coding, the following steps were implemented (Kümbetoğlu, 2008): (1). Exploratory discovery (identifying sequences, words, and phrases within their spoken context), (2). Coding during discovery (decomposing, organizing, and categorizing data), (3). Theory-based coding, (4). Reorganizing coded segments, (5). Adding analytical memos, (6). Rendering indicators within the data visible, (7). Establishing links between different segments of the text and various files, (8). Verifying ideas and opinions by querying coded schemas and databases to form subgroups, (9). Mapping ideas and opinions; modeling processes, relationships, and connections, (10). Presenting the final report and conclusions.

To ensure the quality of the coding process, Gibbs’ recommendations were integrated (as cited in Kümbetoğlu, 2008): (1). Achieving total command of the data by the researchers, (2). Maintaining sensitivity to the context of the data, (3). Generating new codes, modifying existing ones, or avoiding specific codes while paying attention to interconnected ones during the process, (4). Recording the criteria for why specific codes were selected, (5). Considering alternative codes and interpretations of the data.

The research team performed the qualitative analysis in light of this framework. Regarding the technical execution of the analysis, the research team decided to use manual analysis rather than computer-aided qualitative data analysis software. The audio and video recordings collected during in-depth interviews were first transcribed into written texts. The transcripts were read repeatedly, analytical categories were noted, and each was assigned a code number. Codes identified during the initial readings were recorded and numbered sequentially. As new codes emerged, previous texts were re-examined to determine whether they contained these new categories. This iterative process continued until no new categories (codes) emerged, as suggested by Fielding (Kümbetoğlu, 2008). Upon completion of the text scanning and coding process, 34 distinct codes were identified across 22 transcripts, derived from audio and video recordings of 22 participants.

In the subsequent stage of the analysis, the frequency of each code, memo, and metaphor was determined for every individual participant. Prior to reaching the final conclusions, the process of forming subgroups by querying the codes—as outlined in the eighth step by Lewins [cited in Kümbetoğlu (2008)]—was implemented. At this juncture, through a process of thematic synthesis and investigator triangulation, the 34 initial codes were consolidated into four distinct subgroups. These subgroups, referred to as the potential culture code, were identified as Home, Freedom, Migration, and Ego Satisfaction. While some codes correspond to a single subgroup based on their meaning and structure, others may overlap across multiple subgroups. For instance, “Code 1: Using a massive machine, powerful car, greatness” aligns with the “Ego Satisfaction” subgroup, and “Code 2: Comfort” corresponds to the “Home” subgroup. Conversely, “Code 10: Coping with harsh conditions, achieving, and deriving pleasure from it” intersects with both the “Migration” and “Ego Satisfaction” subgroups. The comprehensive list of codes corresponding to each of the four subgroups is presented in Table 1.

**TABLE 1:** Codes and subgroups data table.

			Subgroups (potential culture codes)			
Code	Code description	Frequency of each code out of 22 participants	Home	Freedom	Migration	Ego satisfaction
Code 1	Driving a huge machine, powerful car, sense of greatness	16				16
Code 2	Comfort	10	10			
Code 3	Not everyone can do it, rarity, uniqueness, reputation, prestige, pride	16				16
Code 4	Cleanliness	7	7			
Code 5	Living space	15	15		15	
Code 6	Ostentation, beauty, aesthetics	6				6
Code 7	Dangerous journey, uncertainty, excitement	5			5	
Code 8	Safety anxiety, stress	13			13	
Code 9	Help, solidarity, support in bad times, acquiring an environment	8			8	
Code 10	Coping with difficult conditions, succeeding, enjoying it	10			10	10
Code 11	Life on the road is beautiful, enjoyable, fun	8		8	8	
Code 12	Geographical freedom, traveling, resting	15		15	15	
Code 13	Freedom to not account to anyone, it’s hard to deal with people	10		10	10	10
Code 14	Being natural, being straightforward	1		1		
Code 15	Reaction against Authority, State, NGOs	10			10	
Code 16	Oral culture, conversation	1			1	
Code 17	Disrespect, belittlement, contempt	13			13	
Code 19	Become a wise man; have a lot of memories	7			7	7
Code 20a	Patience, hard work, endurance, ambition	4			4	4
Code 20b	Protection: Family, burden, trust, asset	6	6		6	
Code 21	Freedom from family, father, puberty	14		14		14
Code 22	Loneliness, sadness, longing, suffering	14			14	
Code 23	Comrade, friend, confidant, companion, personification	9			9	
Code 24	Fear as a child	2				
Code 25	A passion for cars from a young age	6				
Code 26	Accommodation place	3	3		3	
Code 27	Fondness for innovation, fondness for firsts, technology	3			3	3
Code 28	Conflict with the settlers	2		2	2	
Code 29	Common property, kin, cooperation, bond, community	2			2	
Code 30	Trucking with family or friends	2	2		2	
Code 31	Respect for the law, compliance with the rules.	2			2	
Code 32	Upgrade, class difference, symbolizing it with property	1			1	1
Code 33	The joy of returning home, peace, confidence	1	1			
	**Frequency of the codes for each subgroup**	**344**	**44**	**50**	**163**	**87**
	**Percentage of the codes for each subgroup**	**100%**	**13%**	**15%**	**47%**	**25%**

In the final stage of the analysis, the frequencies of the codes for the four subgroups were calculated from the interview transcripts. In total, 34 codes were repeated 242 times by 22 participants. These 34 codes were allocated into 4 subgroups (potential culture codes). Since some codes can be categorized under more than one subgroup, there are 46 repetitions of codes under four subgroups, and there are 344 repetitions of codes for 22 participants based on subgroups. Out of 46 repetitions, seven codes were determined for the “Home” subgroup (15%), 6 codes (13%) for the “Freedom” subgroup, 23 codes (50%) for the “Migration” subgroup, and 10 codes (22%) for the “Ego satisfaction” subgroup. Out of 344 repetitions, 44 codes were repeated (13%) for the “Home” subgroup, 50 codes for the “Freedom” subgroup (15%), 163 codes for the “Migration” subgroup (47%), and 87 codes for the “Ego satisfaction” subgroup (25%). According to these data, the “Migration” subgroup accounted for 50% of 46 code types and 47% of 344 individual code occurrences.

To ensure analytical rigor and minimize individual bias, a peer debriefing and investigator triangulation approach was adopted. The researchers independently reviewed the initial codes, and any discrepancies were resolved through collaborative scholarly discussion until a 100% consensus was reached on the final four subgroups.

Consequently, the “Migration” subgroup emerged as the most predominant category. The frequency and depth of the codes associated with this theme were so robust and consistent across the participants that “Migration” was identified as the primary cultural code. This conclusion is based on the qualitative density and thematic saturation observed throughout the analysis, suggesting that “Migration” serves as the fundamental cultural code in the unconscious of truck drivers in Northwest Anatolia regarding the truck as a product.

By the nature of qualitative research, evaluating the analysis results through expert peer review, examining the structure and content from various perspectives, and engaging in a process of synthesis and argumentation enhances the robustness of the study. Therefore, the analysis results, codes, and subgroups shared above were subjected to collaborative scholarly discussion. The research team for Study 1 consisted of 4 researchers: the corresponding author, the psychotherapist who led the data collection phase, which was in-depth interviews using a psychoanalytic and transactional analysis-based approach, and two professors in marketing. All members of the research team are in the author team. These sessions, conducted in the format of an investigator triangulation protocol, covered the data collection methodology, in-depth interview questions, transcriptions, the coding process, and the clustering of codes into subgroups. These scholarly deliberations culminated in a consensus that the theme of “Migration” serves as the cultural code for the truck within the unconscious of the selected target audience.

### 2.4 Discussions: migration and its place in Turkish culture

After the discovery of the cultural code of the truck as the concept of migration, an interdisciplinary and in-depth literature research was conducted in the fields of history, sociology, ethnology, and art within the context of Turkish culture. The literature research aligns with the qualitative findings, providing a robust historical and cultural foundation for this code. Within this framework, migration is defined as the movement of individuals or groups from one country or settlement to another, driven by economic, social, and political factors (Çimen, 2021). However, beyond its functional definition, for Turkish culture, the continuous repetition of this migratory experience and the associated intense emotional states—particularly historical traumas—have enabled migration to transcend mere eventhood and become a foundational cultural code. Migrations experienced by Turkish culture throughout their history can be categorized into three main pillars: great mass migrations (driven by territorial shifts or disasters), nomadism (seasonal economic subsistence), and commercial mobility (such as the Silk Road trade) (see Supplementary Material D). Individuals and society are deeply affected by these movements across economic, social, political, and psychological dimensions. The emotional load, trauma, and tragedies associated with these migrations have left indelible traces in social and cultural elements. The more pervasive the effect of the migration phenomenon across various cultural elements, the more effectively it is embedded in the social culture and the social unconscious. This impact is evident in literary works, folk literature, mythological stories, epics, fairy tales, folk songs, folk poems, minstrel literature, modern literature, cinema, theater, architecture, music and in painting (see Supplementary Material D).

While migration is a catalyst for change that can lead to the disappearance of certain cultural markers, it can also serve as a central cultural code due to its repetitive nature and emotional intensity. At this juncture, it is essential to distinguish between the truck’s functional utility as a tool for “hauling products” and its symbolic resonance in the cultural unconscious. In conjunction with the concept of migration, one must consider the concepts of mobility, relocation, and transportation. In the modernization process, the vehicles experienced by society as means of transportation—such as horses, camels, and caravans—have been superseded by the truck. Consequently, the truck serves as the modern technological successor to these ancestral vehicles, bridging the gap between contemporary labor and historical migratory identity (Çimen, 2021; Gökçe and Eroğlu, 2017; Vural, 2017). Through this symbolic continuity, the truck becomes identified with the “Migration” code, representing the ancestral experience of being “on the move” in a modern industrial context. It is important to emphasize that the “migration” code was not imposed as a top-down sociological concept but emerged bottom-up from the drivers’ own in-depth interviews. For them, it represents an unconscious cultural code rather than an argument shaped by a popular political or socio-cultural agenda.

## 3 Study 2: fMRI study to test the unconscious cultural code of the product

### 3.1 Conceptual basis of Study 2

Consumer neuroscience is defined as the scientific study of consumer behavior through the integration of neuroscience with psychology and economics. It aims to adapt theoretical knowledge and neuroscientific methods to develop a better understanding of consumer psychology and marketing theory. It is primarily an academic and research-oriented field that focuses on identifying the neural mechanisms underlying consumer choices and preferences. Neuromarketing is defined as the practical application of neuroscientific tools in the corporate and commercial sectors. It seeks to apply findings from consumer neuroscience to specific commercial problems, such as product design, branding, and advertising. It is a practitioner-oriented field that aims to improve marketing efficiency and sales by directly measuring brain activity in response to marketing stimuli (Javor et al., 2013; Ramsøy, 2019). Many neuroscientists and marketing professionals have taken advantage of neuroimaging technology to explore consumer’s response to various marketing elements, such as products, packaging, advertising or consumer financial decisions (Adalarasu et al., 2025; Bajaj et al., 2023; Bansal et al., 2025; Hamelin et al., 2025; Kalaganis et al., 2021; Casado-Aranda et al., 2021; Oliveira et al., 2022; Xu et al., 2023; Zhao and Wang, 2021), helping to improve marketing strategies (Kajla et al., 2023). The use of neuroscience tools has improved the understanding of cognitive, neuronal, and emotional mechanisms related to marketing-relevant behavior, and given a more profound understanding of the decision-making process and consumer behavior, affected by emotion, attention, and memory (Alsharif et al., 2025; Alvino et al., 2020; Hamelin et al., 2025).

In Study 2, fMRI, a neuroscientific imaging method, was used within the scope of quantitative research. fMRI was selected for its high spatial resolution, enabling investigation of deep-seated neural activations. Study 1 was an exploratory, qualitative research study that claims the unconscious cultural code of the product “truck” is migration. Study 2 aims to take this output as input to test it. In this case, the hypothesis of Study 2 would be as follows: “The unconscious cultural code of the product truck is migration.” However, this hypothesis statement cannot be tested using fMRI. To test this hypothesis, it was necessary to reformulate it as an equivalent hypothesis that could be tested within a research model from the neuroscience literature. To design the model of the fMRI task and the final hypothesis, a neuroscientific literature review regarding the encoding and recall of unconscious codes was carried out in a comprehensive review format (Çimen, 2021). As a result of this review (see Supplementary Material E), it is determined that there are studies in the literature where two different concepts are related and encoded to each other in an unconscious experiment setup (unconscious relational encoding) and tested whether this relationship is remembered at a conscious and/or unconscious level (conscious or unconscious retrieval) (Duss et al., 2014; Hannula and Greene, 2012; Henke et al., 2003b; Reber et al., 2014; Züst et al., 2015).

Henke et al. (2003b) designed a series of unconscious and conscious encoding and retrieval experiments, in which face and profession matching were performed. This was the first finding on the role of the hippocampal formation in unconscious encoding and retrieval processes in the human brain. With this study, during unconscious encoding and retrieval, right and left hippocampal formation, right perirhinal cortex, bilateral fusiform regions (right and left fusiform gyrus), left middle temporal gyrus, left middle and superior temporal gyrus, right and left superior temporal gyrus, right inferior frontal gyrus regions were found to be activated.

In a similar experiment by Henke et al. (2003a), both the accuracy of the answers and the reaction time are measured, and it is investigated whether reaction time differs between correct and incorrect responses. When brain activations were controlled in the fMRI task, it was observed that the regions where the experimental group was more activated than the control group during coding were the posterior and anterior cingulate, pre/postcentral gyrus, superior and inferior parietal lobe, angular gyrus, fusiform gyrus, lingual gyrus, cuneus, inferior and middle frontal gyrus, middle and superior temporal gyrus. During the retrieval, it was determined that the regions where the experimental group was more activated than the control group were hippocampal and perirhinal, middle and superior temporal gyrus, temporal pole, superior temporal and supramarginal gyrus, inferior frontal gyrus, middle and superior frontal gyrus, orbital and superior frontal/medial frontal and anterior cingulate gyrus, middle occipital gyrus, retrosplenial gyrus, precuneus, putamen, angular gyrus, superior parietal lobe and precentral gyrus, middle and bilateral superior occipital gyrus. Additionally, in parallel with the knowledge that the components of the medial temporal lobe — namely, the hippocampus, perirhinal, entorhinal, and parahippocampal cortex — make distinct contributions to memory, the frequency of activation of each region was measured in a subset of participants. These findings are also consistent with Elliot and Dolan’s experiment with Japanese ideograms, in which activation of the right parahippocampal gyrus, left mediodorsal thalamus, left fusiform gyrus, left superior temporal gyrus, and right cuneus was observed (Henke et al., 2003a).

In the research by Lau and Passingham on the cognitive control system (2007), participants’ reactions were measured against the instructions given, based on the objects displayed at the unconscious (subliminal) level. Here, too, activation differences were observed in the left ventral premotor region for phonological decisions and in the middle temporal gyrus and left inferior frontal cortex for semantic decisions, depending on whether the instructions during unconscious encoding were compatible or incompatible.

In the study by Reber et al. (2012), participants were first subliminally coded with related and unrelated word pairs. In the experiment, it was found that there was more hippocampal activity in matched word pairs than in non-matches during unconscious coding, and more hippocampal activity in related words than in unrelated words during unconscious retrieval. Moreover, the increase in hippocampal activity during unconscious coding also enabled the prediction of the decision-making process’s outcome. In this study, it was determined that all relevant cognitive processes of the participants (coding, recalling, associating, and inferring) occurred at an unconscious level, and there were extensive activations in the medial temporal regions, particularly in the anterior hippocampus. More specifically, it was determined that participants who made relational inferences exhibited greater activation in the posterior cingulate gyrus region during encoding, as well as in the parahippocampal cortex, anterior cingulate gyrus, and middle occipital gyrus regions during recall. Previous studies have identified that the posterior cingulate gyrus is a multimodal junctional center that integrates recalled and newly presented information (Reber et al., 2012). A similar activation was also observed in their study.

When it comes to relational retrieval, it is observed that the medial temporal lobe and neocortex areas are more activated in unconscious processes than in conscious processes, and on the contrary, in conscious processes, the hippocampal anterior thalamic axis and its connections to the neocortex are more activated than in unconscious processes (Duss et al., 2014). In the study by Duss et al. (2014), 11 amnesic patients and 11 participants from a normal control group were examined on the retrieval of relational encoded concepts using single-item retrieval. When the retrieval performance of subliminally encoded words was measured, it was observed that the single-item recall performance was the same in both groups, as it activated the neocortex. In contrast, the amnesic patient group failed at relational retrieval, whereas the participants in the control group succeeded. While generally unconscious relational retrieval appears to be supported by hippocampal and neocortical activation, more specifically, the control group showed greater activation in the bilateral hippocampus, right thalamus, bilateral medial prefrontal cortex, and anterior cingulate during relational retrieval.

In the fMRI experiment conducted by Züst et al. (2015), after matching the face and profession at the subliminal level, response time and region of interest were observed at the conscious and unconscious. It was observed that response time decreased when participants were asked to identify the profession to which a face matched a subliminally coded face-profession pair. Meanwhile, activation was observed in the hippocampus, temporal pole, superior temporal sulcus, angular gyrus, and precuneus/calcarine sulcus regions (Züst et al., 2015).

Any hypothesis test about a society needs to be conducted with its members. The literature on the relationships among social, cultural, collective, and individual memory (Assmann, 2008; Markowitsch, 2008; Welzer, 2008) complements the concept of relational encoding and retrieval at the unconscious level. In this way, it is possible to trace a society’s unconscious cultural code in the unconscious minds of its members.

In line with the conceptual basis, if two concepts, truck and migration, are relationally encoded in the unconsciousness of the participants, this can be a powerful argument that they are encoded in the cultural unconsciousness of the participants who represent Turkish culture, therefore one of them is the cultural code of the other one; in this case, it can be said that the migration is the cultural code of the truck. This finding would be consistent with the exploratory result from Study 1.

Therefore, the new and final version of the hypothesis could be defined as follows:

H1: The concepts of truck and migration are relationally encoded in the cultural unconscious of the truck drivers in the target population.

To test the hypothesis, control variables were needed. The research team conducted a sub-study to define the control variables rather than relying on experience-based selection. The marketing communication studies of the two brands, which are the leaders of the Turkish truck market over the years, were used (Ford Otosan, 2015a,b). In this study, 38 past communication studies for the first heavy truck brand and 44 for the second heavy truck brand were scanned. As a result of the qualitative analysis, the theme of the economy was chosen (see Supplementary Materials F, I). As the final step, the “neutral” background (Z) was selected as the second control variable in the study (see Supplementary Materials F, I; Çimen, 2021).

Based on the model found in the literature, it can be said that in this study, there is the “truck and migration” pair, similar to the “face and profession” pair. It is desired to determine whether these two concepts are relationally encoded in the target society’s cultural unconscious over the historical timeline and whether “migration” serves as the unconscious cultural code within that unconscious. In the fMRI experiment, when two images, “truck image + migration image at background,” are shown together as visual stimuli, if similar activation occurs in the same regions as in the literature, then it can be said that there is a possibility that these two concepts are unconsciously related. Since an unconscious relation is not expected in control visuals, “truck + economy” and “truck + neutral” are not expected to activate in these regions. Thus, the research hypothesis can be tested using quantitative data.

**FIGURE 1:**
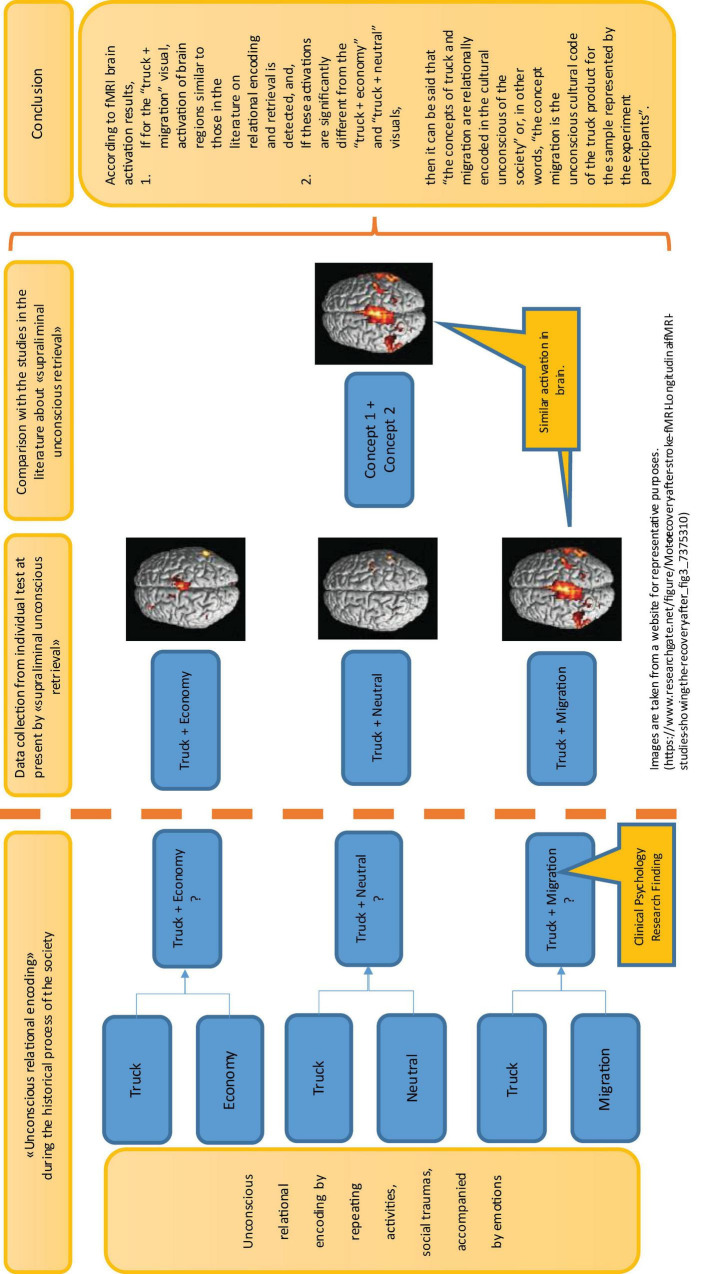
Functional Magnetic Resonance Imaging (fMRI) experiment model (schematic representation of the fMRI task paradigm).

Starting from this point, the research model is constructed as follows: The experimental variable, “migration,” which is claimed to be the unconscious cultural code, and the control variables “economy” and “neutral” are transformed into background pictures. Truck images are placed on these background pictures. In the fMRI experiment, the visuals are shown to the participants supraliminally unconsciously, accompanied by a question to keep the participants’ focus on the truck pictures. In this model, by placing several different truck images on three background images that represent the “migration” and control images “economy” and “neutral,” the backgrounds will be investigated to determine whether there are significant differences between them. According to fMRI brain activation results, if for the “truck + migration” visuals, activation of brain regions similar to those in the literature on relational encoding and retrieval is detected, and if these activations are significantly different from the “truck + economy” and “truck + neutral” visuals, then it can be said that the concepts of “truck” and the concept of “migration” are relationally encoded in the cultural unconscious of the society or, in other words, the concept “migration” is the unconscious cultural code of the “truck” product for the sample represented by the experiment participants. Therefore, the model of the fMRI experiment can be visualized as in Figure 1.

### 3.2 Materials and methods

#### 3.2.1 Preparation of stimuli (visuals)

**FIGURE 2:**
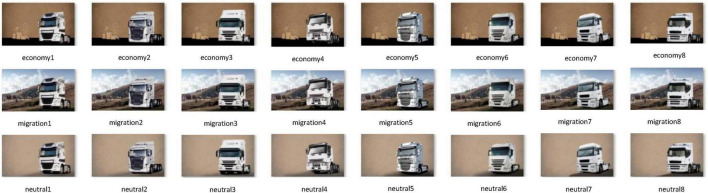
A total of 24 visuals obtained with eight trucks, three background themes.

The creation of images or visuals as stimuli and the preparation of the design experiment to be displayed during the fMRI task are critical processes in the research to determine the unconscious effect of the migration theme on participants’ brains. At this stage, following steps were completed sequentially: research of the truck images, transforming them into visuals by a visual arts expert, brand guess survey with a group of truck drivers and a group of industry experts to eliminate the noise factors of brands, truck advertisement research to define the control variables, background image selection survey with truck drivers, combination of the images by the visual art expert (see Figure 2), the experimental design and its simulations to optimize the estimation efficiency and detection power, software programming to define the sequence, duration and interval timings of the images, and final visual preparation for fMRI task. The fMRI task paradigm was structured with the following technical parameters: The stimulus set comprised 24 unique combinations derived from 8 distinct truck images and 3 thematic backgrounds. To ensure robust signal acquisition, each truck visual was presented 9 times across the task. The background conditions were distributed as follows: Background 1 (Economy) was presented 22 times, Background 2 (Neutral) 17 times, and Background 3 (Migration) 33 times. Each stimulus was presented for a duration (SD) of 3 s, interleaved with a jittered inter-trial interval (ITI) ranging from 2 to 4 s to optimize the detection power (the measure of the ability to detect the activation) and estimation efficiency (the measure of the ability to predict the shape of the hemodynamic response). Detailed specifications regarding stimulus randomization, counterbalancing, and genetic algorithm-based paradigm optimization are provided in Supplementary Materials F–J.

#### 3.2.2 Participants

Logistics companies, cooperatives, and associations were contacted to find participants. The study was completed on seven separate days, 34 people participated, all male, Mage = 46, minimum 31, maximum 65, SD = 9, 14 of them were born in Northwest Anatolia, 34 people work in Northwest Anatolia, 11 of them are primary school graduates, 11 of them are secondary school graduates, 11 of them are high school graduates, and one of them are associate degree graduate, 29 of them are married, three of them are single, two of them are widowed. The average number of children per participant was two, with a minimum of 0, a maximum of 5, and an SD = 1. The average seniority in the profession is 13 years, with a minimum of 1 and a maximum of 43, SD = 11. As a profession, there are 34 drivers, three who are both drivers and vehicle owners, 21 who drive trucks, 28 who drive tow trucks, and 21 who drive both trucks and tow trucks. All participants reported no history of neurological disorders or current psychiatric diagnoses, with the exception of one individual who reported a remitted depressive episode not requiring current intervention. Standard fMRI safety screening was applied: One participant was excluded due to the presence of a cardiac stent (MRI contraindication), while two participants with dental prostheses were included after being cleared by the imaging staff for safety and image quality. Handedness was assessed, with 31 participants right-handed and three ambidextrous, all capable of performing the task with their dominant hand. Regarding visual requirements, participants either had normal or corrected-to-normal vision; 15 individuals utilized MR-compatible corrective measures or had impairments that did not interfere with stimulus perception. Following one participant’s voluntary withdrawal during the scan, the final fMRI study was completed with 33 participants.

#### 3.2.3 Data collection

In this process, the neuroscience laboratory, the fMRI device and its auxiliary equipment, optical devices, software for displaying stimuli, and software for designing the experiment were utilized as tools. Preliminary information about working on the phone was given to all participants. The study was conducted over seven different group sessions, each held on a separate day. Verbal and written information was given to the volunteer participants. In line with the Ethics Committee requirements (Koç University, Decision Number: 2017.146.IRB052, Date: 30.10.2017), signatures were obtained on the Consent Form and Permission to Use Personal Health Information for Research Purposes. Using the Participant Information Form (see Supplementary Material C), demographic, occupational, and health data were collected to assess participants’ suitability for the task. Verbal information was given to each participant again just before the task. Information about the setup was provided without mentioning the task’s technical design or objectives. It was stated that when they enter the fMRI device, they will be given a button in their right hand. Initially, there will be a calibration time of about 6 min, during which they will see a flower image on the screen. It was informed that after the calibration, the radiologist would warn that the task would begin via the microphone, and they would be shown the truck pictures, and they were asked to press the button of the handset once when they liked these truck pictures less, twice when they liked them moderately, and three times when they liked them very much. After the truck pictures were shown, a 2-min wait passed, and the task was announced to be terminated via the microphone. Necessary warnings and regulations were provided regarding the fMRI general protocol (focus on staying awake, panic button, no metal objects to be kept inside, staying still, earplugs, fixation apparatus, screen and reflector adjustment, privacy warning). The task took approximately 15 min, including 6 min of preset structural imaging and calibration, 7 min (410 s) for the task itself, and approximately 2 min of final wait.

A 3T MRI (Magnetic Resonance Imaging - Siemens Magnetom) equipped with a 64-channel head coil was utilized for MR scanning. The MRI protocol started with structural scanning using a T1-weighted MPRAGE (Magnetization Prepared Rapid Gradient Echo) sequence. The parameters for this sequence were set as follows: TR/TE = 1,900/2.52 ms, voxel size of 1 × 1 × 1 mm, 192 slices in interleaved order, 250 mm FOV, TI = 900 ms, flip angle of 9 degrees, base resolution = 256, and a scan time of 3 min and 6 s. Subsequently, BOLD (Blood Oxygen Level Dependent) images were acquired using an fMRI sequence with EPI readout. The parameters for this sequence included: TR/TE = 3,000/30 ms, voxel size of 3 × 3 × 3 mm, 47 slices, flip angle of 85 degrees, 144 measurements, and a scan time of 7 min and 23 s.

### 3.3 Analysis and results

In this study, fMRI analysis was performed using FEAT (Functional Magnetic Resonance Imaging Expert Analysis Tool) version 6, an integral component of FSL (Functional Magnetic Resonance Imaging of the Brain’s Software Library) (FSL, 2020). The preprocessing phase involved several steps, including motion correction (Jenkinson et al., 2002), skull stripping (Smith, 2002), spatial smoothing with a 5 mm Gaussian kernel, and slice time correction. At the subject level, distinct explanatory variables (EVs) were defined for each stimulus type (background images of economy, neutral, and migration), and for each, the on-off time signal was convolved with a gamma function to generate the expected BOLD response. Temporal derivatives of these expected responses were also incorporated into the model. The resultant data were then fed into the model (Woolrich et al., 2001) to derive mean activations and activation differences for all EVs. Z (Gaussianised T/F) statistical images were thresholded using clusters defined by Z > 2.3 and *p* = 0.05 as the cluster significance level (Worsley, 2001). Group-level analyses were conducted utilizing FSL’s mixed-effects analysis tool (FLAME). The General Linear Model (GLM) encompassed a group mean Z significance level of 2.3 and a cluster p significance level of .01 for the cluster threshold level.

Mean activation and activation differences for each stimulus type were quantified using cluster-corrected z-images. As these z-images had been registered to standard space, a custom script was developed to extract the names of brain regions, including both cortical and subcortical structures, and distinguish between left and right hemispheres, where statistically significant activation or changes in activation between stimulus types were observed. The number of activated voxels in these regions was also quantified, and only regions with more than three active voxels were included in the report.

**FIGURE 3:**
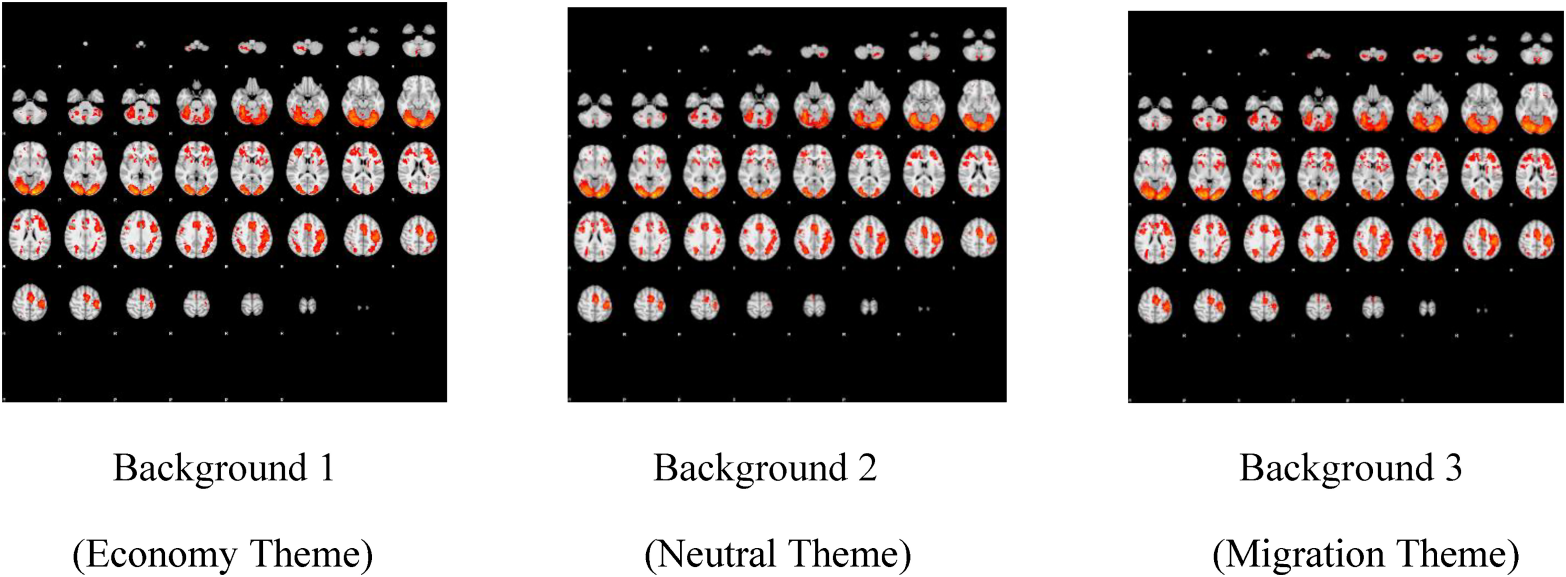
Average activities of backgrounds.

**FIGURE 4:**
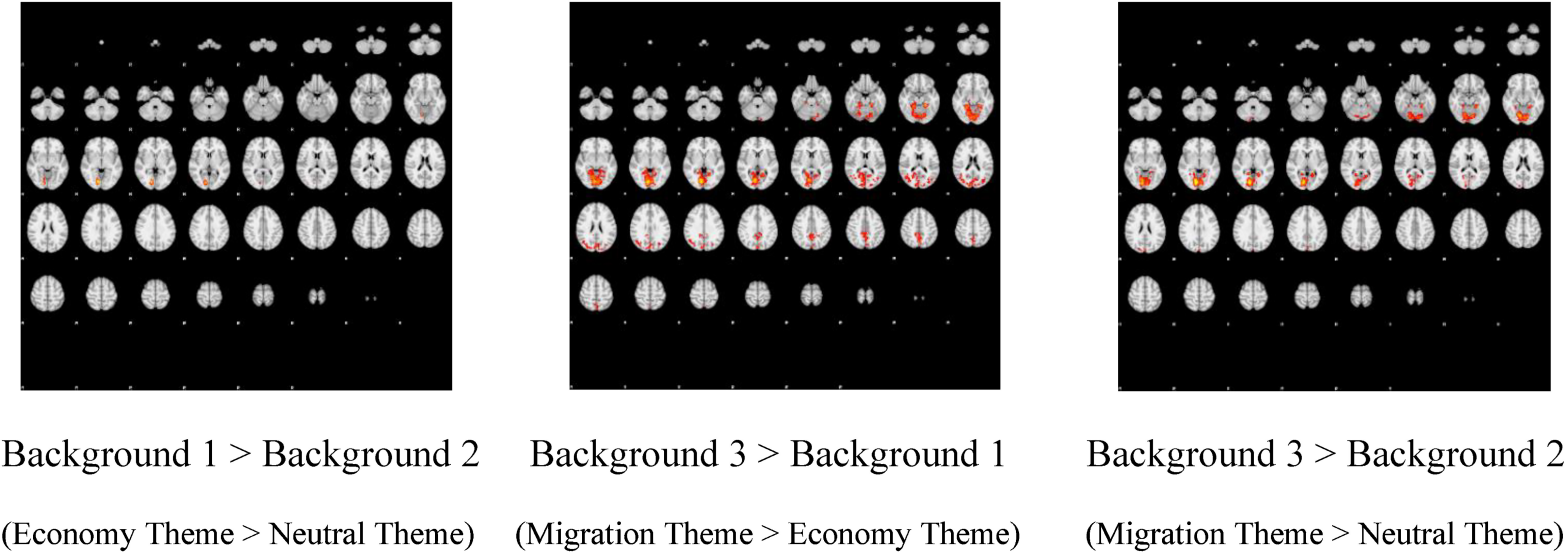
Comparison of activations for each background.

The data are presented in two complementary formats: first, through figures (Figures 3, 4) illustrating activations and differences in activations, and second, in statistical tables (Table 2) detailing activations, including coordinates, z-statistics, and cluster size, with a distinction between right- and left-brain regions. For each of these, the mean and the difference in activations (if present) were calculated for Background 1 (economy theme), Background 2 (neutral theme), and Background 3 (migration theme). Therefore, for each Background, average activation figures and tables, Background 1 > Background 2, Background 3 > Background 1, and Background 3 > Background 2 figures and tables were evaluated. Here, the symbol > (greater than) does not represent mathematical magnitude; rather, it indicates a statistically significant difference in activation. In other words, the phrase “Background 1 > Background 2” means that the regions where the images with the Background 1 (economy theme) created a statistically significant difference in activation compared to the images with the Background 2 (neutral theme). In addition, although Background 1 and Background 2 generated some activations on average, they did not yield statistically significant activations in any region, as determined by comparing Background 1 to Background 3, Background 2 to Background 1, and Background 2 to Background 3. In other words, there is no region that Background 1 activates with a significant difference compared to Background 3; and Background 2 did not create a significant difference in activation in any part of the brain compared to Background 1 or Background 3. For this reason, the figures and tables for Background 1 > Background 3, Background 2 > Background 1, and Background 2 > Background 3 are eliminated and not shown. To facilitate the analysis, the data are organized as follows: Background 1 > Background 2, Background 3 > Background 1, and Background 3 > Background 2, as shown in Table 2. All brain regions in which activation was observed, including means and differences, were included in the table. Upon examining both the figures with anatomical representation and the tables, the following summary was observed: Regions where Background 1 shows significantly higher activation than Background 2 include the intracalcarine cortex, the lingual gyrus, the occipital fusiform gyrus, and the occipital pole. Background 3 created activation in many regions with a significant difference compared to both Background 1 and Background 2. The areas activated by Background 3 with a significant difference compared to Background 1 are postcentral gyrus, angular gyrus, lateral occipital cortex superior division, lateral occipital cortex inferior division, intracalcarine cortex, cingulate gyrus, posterior division, precuneus, cuneus, parahippocampal gyrus posterior division, lingual gyrus, temporal fusiform cortex posterior division, temporal occipital fusiform gyrus, supracalcarine cortex, occipital pole, middle temporal gyrus temporooccipital part. The regions activated by Background 3 with a significant difference compared to Background 2 are largely similar to the previous one: lateral occipital cortex superior division, intracalcarine cortex, cingulate gyrus, posterior division, precuneus, cuneus, parahippocampal gyrus posterior division, lingual gyrus, temporal fusiform cortex posterior division, temporal occipital fusiform cortex, occipital fusiform gyrus, supracalcarine cortex, occipital pole (see Supplementary Material K).

**TABLE 2:** Comparison of backgrounds by brain activations.

	Background 1 > Background 2									
	Left cluster					Right cluster				
Label	Size	z-stats	Coordinates			Size	z-stats	Coordinates		
			x	y	z			x	y	z
Postcentral gyrus										
Angular gyrus										
Lateral occipital cortex, superior division										
Lateral occipital cortex, inferior division										
Intracalcarine cortex						102.0	4.801	14	−80	2
Cingulate gyrus, posterior division										
Precuneous cortex										
Cuneal cortex										
Parahippocampal gyrus, posterior division										
Lingual gyrus						234.0	4.740	14	−74	−2
Temporal fusiform cortex, posterior division										
Temporal occipital fusiform cortex										
Occipital fusiform gyrus						3.0	2.737	16	−74	−10
Supracalcarine cortex										
Occipital pole						6.0	2.600	16	−90	2
Middle temporal gyrus, temporooccipital part										
	**Background 3 > Background 1**									
	**Left cluster**					**Right cluster**				
**Label**	**Size**	**z-stats**	**Coordinates**			**Size**	**z-stats**	**Coordinates**		
			**x**	**y**	**z**			**x**	**y**	**z**
Postcentral gyrus						3.0	2.592	12	−38	50
Angular gyrus						20.0	3.111	48	−58	32
Lateral occipital cortex, superior division	231.0	3.152	−38	−80	22	478.0	3.815	34	−76	20
Lateral occipital cortex, inferior division	31.0	3.001	−42	−72	14	29.0	2.724	44	−68	14
Intracalcarine cortex	72.0	3.250	0	−80	4	499.0	5.701	12	−82	0
Cingulate gyrus, posterior division	107.0	3.268	−10	−44	−2	203.0	4.001	6	−36	43
Precuneous cortex	622.0	4.088	−20	−58	4	464.0	3.971	2	−60	48
Cuneal cortex	162.0	3.797	−4	−74	24	195.0	3.834	4	−80	26
Parahippocampal gyrus, posterior division	116.0	5.109	−20	−38	−16	72.0	3.607	22	−38	−14
Lingual gyrus	938.0	4.638	−20	−42	−14	1356.0	5.748	6	−80	−4
Temporal fusiform cortex, posterior division	40.0	4.277	−24	−38	−18	9.0	3.470	22	−38	−18
Temporal occipital fusiform cortex	47.0	3.944	−22	−44	−16	97.0	4.506	22	−48	−14
	**Background 3 > Background 1**									
	**Left cluster**					**Right cluster**				
**Label**	**Size**	**z-stats**	**Coordinates**			**Size**	**z-stats**	**Coordinates**		
			**x**	**y**	**z**			**x**	**y**	**z**
Occipital fusiform gyrus	78.0	3.823	−18	−70	−14	77.0	4.009	20	−70	−12
Supracalcarine cortex	37.0	3.261	0	−74	20	107.0	3.935	4	−78	12
Occipital pole	91.0	3.409	−8	−92	20	238.0	4.269	8	−90	−4
Middle temporal gyrus, temporooccipital part						4.0	2.684	40	−56	12
	**Background 3 > Background 2**									
	**Left cluster**					**Right cluster**				
**Label**	**Size**	**z-stats**	**Coordinates**			**Size**	**z-stats**	**Coordinates**		
			**x**	**y**	**z**			**x**	**y**	**z**
Postcentral gyrus										
Angular gyrus										
Lateral occipital cortex, superior division						28.0	3.402	22	−84	24
Lateral occipital cortex, inferior division										
Intracalcarine cortex	53.0	3.107	−16	−62	6	419.0	6.060	12	−82	2
Cingulate gyrus, posterior division	11.0	3.222	−18	−50	2	13.0	3.452	18	−48	2
Precuneous cortex	142.0	3.661	−20	−58	6	86.0	3.555	20	−56	10
Cuneal cortex	35.0	3.188	0	−80	36	33.0	2.834	10	−86	32
Parahippocampal gyrus, posterior division	63.0	4.270	−24	−42	−14	12.0	3.087	22	−38	−16
Lingual gyrus	770.0	4.684	−8	−82	−10	915.0	6.054	14	−82	−6
Temporal fusiform cortex, posterior division	28.0	4.335	−26	−42	−14	7.0	3.141	24	−38	−18
Temporal occipital fusiform cortex	15.0	3.802	−24	−44	−14	51.0	3.276	22	−40	−18
Occipital fusiform gyrus	37.0	3.302	−14	−80	−10	133.0	4.482	16	−74	−12
Supracalcarine cortex	13.0	2.593	0	−74	18	34.0	3.170	20	−66	16
Occipital pole	17.0	3.136	−2	−90	28	221.0	4.605	12	−90	−2
Middle temporal gyrus, temporooccipital part										

For a more comprehensive interpretation of the results in Figures 3, 4, and Table 2, it is worth noting that the experimental design was implemented according to the principle of Cognitive Subtraction. Under this framework, BOLD signals generated by the Backgrounds (experimental conditions) were subtracted from the baseline BOLD signals; these activations were subsequently presented via neuroimaging visualizations and statistical tables. First, the BOLD signal differentials—representing neural activations—for each background were visualized in Figure 3 relative to the rest position (passive baseline). In this context, the actual experimental conditions are represented by the neural activations observed during the presentation of specific thematic backgrounds, relative to the baseline state. Following this, the BOLD signal contrasts for each background were visualized in Figure 4, utilizing the other backgrounds as control variables (active baselines).

### 3.4 Discussion

As stated in the fMRI experiment model, the aim was to determine whether the activations obtained in the task were similar to those in the literature on unconscious level relational encoding and retrieval. According to fMRI brain activation results, if for the “truck + migration” image, activation of brain regions similar to those in the literature on relational encoding and retrieval is detected, and if these activations are significantly different from the “truck + economy” and “truck + neutral” visuals, then it can be said that the concepts of truck and migration are relationally encoded in the cultural unconscious of the society or, in other words, the concept migration is the unconscious cultural code of the truck product for the sample represented by the experiment participants. If for the “truck + migration” image, activation of brain regions similar to those in the literature on relational encoding and retrieval cannot be detected, or if these activations are not significantly different from the “truck + economy” and “truck + neutral” visuals, then it cannot be said that the concepts of truck and migration are relationally encoded in the cultural unconscious of the society or, in other words, it cannot be said that the concept X is the unconscious cultural code of the truck product for the sample represented by the experiment participants. In parallel, the hypothesis is developed as follows:

H1: The concepts of truck and migration are relationally encoded in the cultural unconscious of the truck drivers in the target population.

If this hypothesis is valid, it can be said that the “truck + migration” relationship obtained by in-depth interviews and qualitative research methods is neuroscientifically supported. Accordingly, when the brain regions in the aforementioned studies were compared with those in this fMRI experiment, similarities were observed in the following brain regions.

The findings of this study align with the previous literature, as noted in the study by Henke et al. (2003b), which reported that regions activated during unconscious learning and recall included the bilateral fusiform gyrus and middle temporal gyrus, findings also observed in this study. Another study by Henke et al. (2003a) reported activation in the superior occipital cortex, middle temporal gyrus, angular gyrus, parahippocampus, precuneus, right cuneus, and left fusiform gyrus, findings similar to those of this study. The middle temporal gyrus, which was activated in the study of Lau and Passingham (2007), was also detected in this study. In the study by Reber et al. (2012), activation of the parahippocampus, posterior cingulate, middle occipital, and fusiform gyrus was also reported. In the study of Duss et al. (2014), it was observed that, more specifically, greater activation was observed in the bilateral hippocampus, right thalamus, bilateral medial prefrontal cortex, and anterior cingulate during relational retrieval in healthy participants in the control group in their experiment, whereas generally unconscious relational retrieval seems to be supported by hippocampal and neocortical activation. Among these regions, it is seen that the common point with this study is the anterior cingulate. The regions detected in the study by Züst et al. (2015) and the common regions identified in this study include the angular gyrus, precuneus, and intracalcarine cortex/supracalcarine cortex.

As part of the discussion, one issue that could potentially receive criticism is that regions such as the occipital lobe and visual cortex, which are heavily involved in visual function, are activated with a significant difference in the brain cross-sectional images of Background 3, which includes the theme of migration. It can be observed that the migration image in Background 3 contains more intense visual elements than the economy image in Background 1. Likewise, the migration image is more intense than the neutral background. For this reason, it can be criticized that the activation is high due to the intensity of the visual stimuli.

Similarly, a potential criticism, as noted regarding the “pretty picture” effect, is that the activation could be driven simply by the aesthetic appeal of comforting elements like clouds or tree-lined hills found in the migration background. Indeed, it is evident that the cerebral cortex, one of the visual centers, is more activated in Background 3 than in Backgrounds 1 and 2. However, there is an activation not only in regions related to vision, such as the occipital cortex, but also in many regions (fusiform gyrus, middle temporal gyrus, parahippocampus / hippocampus, precuneus, angular gyrus, anterior cingulate) that are commonly mentioned in the literature review above. The robust responses observed in these brain regions provide strong evidence against a simple aesthetic explanation. These regions are inextricably linked to relational encoding and memory retrieval, suggesting that the drivers’ brains were processing a specific symbolic narrative—the “Migration” code—rather than merely reacting to a pleasant view from a windshield.

According to these results, similar brain regions in the literature were activated, and these brain regions were activated with a significant difference in the experimental group “truck and migration” compared to the control group “truck and economy,” and “truck and neutral.” Based on this finding, it can be argued that the H1 hypothesis is valid, that is, the concepts of truck and migration are relationally coded in the cultural unconscious of the truck drivers in the target population; in other words, the concept of migration is the unconscious cultural code of the truck product for the target population.

It is essential to bridge the findings of Study 1 with the discussion of Study 2 to understand the neural and cultural mechanisms at play. Throughout a society’s historical trajectory, the processes of social memory formation and the relational encoding of distinct concepts within the cultural unconscious are observable. In this context, the association between the truck and migration can be explained through a systematic learning mechanism: Throughout history, the phenomenon of migration has been repeated for millennia. This repetitive process was consistently accompanied by emotionally intense experiences such as separations, reunions, wars, famines, debacles, social and economic disasters, conquests, victories, and the establishment of new homelands.

In other words, the three critical catalysts of cognitive and social learning—repetition, emotional intensity, and trauma—converged to create a robust unconscious learning environment. In addition, this learning and coding process might be supported by the following three social mechanisms, which were mentioned above: Most of the learning is completed in early childhood, children are under the influence of a single culture (their own culture) to a great extent in early childhood, and psycho-social mechanisms such as language and story phenomena. Within this framework, a permanent link was forged between the phenomenon of migration and its facilitators of mobility such as caravans, horses, horse-drawn carriages and camels.

Through the lens of relational encoding, the human brain does not store the truck as an isolated logistical tool for hauling products; instead, it categorizes the vehicle based on the ancestral “mobility” space it occupies. As the truck is the modern technological successor to the vehicles used in historical mass migrations, nomadism, and commercial mobility, it triggers the pre-existing “Migration” code. Consequently, the truck and migration are relationally encoded in the cultural unconscious, transforming a modern commercial activity into a deep-rooted cultural signifier (Çimen, 2021).

## 4 General discussion and conclusion

Traditional marketing research often relies on conscious self-reports (surveys, focus groups), which can be misleading as they fail to capture the “black box” of consumer cognition. Indeed, the literature indicates that approximately 95% of cognitive processes occur unconsciously.

Against this backdrop, this interdisciplinary research was focused on the cultural unconscious. Similar to the archetypes (the building blocks of the collective unconscious), cultural codes (the building blocks of the cultural unconscious) play a crucial role in shaping purchasing decisions. Since consumers make their decisions largely unconsciously, and in these daily decisions, they frequently refer to the unconscious codes encoded in the culture they are part of. Consequently, these codes serve as the interpretive lens through which a specific society assigns meaning to a product. If the cultural code of a product is discovered in the cultural unconscious of the target society, it can be used as input for marketing strategies and to design and implement marketing mix elements accurately.

Aligning with these findings, this research emphasizes the critical importance of discovering unconscious cultural codes. However, the primary focus of the study is to design a more robust method for the discovery process. This is claimed to be the main theoretical and managerial contribution. Historically, the discovery of unconscious cultural codes relies primarily on qualitative research, such as in-depth reviews, visual narrative arts, enneagram or archetype discovery methods. While valuable, these methods inherently carry the disadvantages of qualitative studies, such as subjectivity and a high dependence on the expertise of the research team. To address these limitations, the main goal of this research is to support the findings of the qualitative research with quantitative research in the area of neuroimaging, specifically fMRI.

Accordingly, in Study 1, a psychoanalytic and transactional analysis-based approach (one of the exploratory and qualitative research methods) was employed via in-depth interviews to discover the unconscious culture code. Subsequently, to increase the power of this research, in the second phase, it was decided to use neuroscientific imaging techniques, specifically fMRI, a causal and quantitative research method, to test the unconscious cultural code claimed to have been discovered previously. By doing so, the output of qualitative research, the unconscious cultural code, was used as an input in quantitative research.

The need to use the right stimuli, which is the primary need of neuroscientific research, and the need to verify the discovered cultural code, which is the primary need of such qualitative research, were met separately, using two methods together and sequentially under the same research framework. Therefore, attractive scientific results, such as filling each other’s gaps and achieving accurate results, have been obtained. By using the qualitative output of Study 1 (the code) as the quantitative input for Study 2 (the fMRI task), this study proposes a holistic, interdisciplinary methodology that bridges the gap between the social sciences (psychoanalysis) and the natural sciences (neuroscience). An innovative and original method was developed, yielding both theoretical contributions and practical implications, particularly in marketing (please see section “1 Introduction” for the details and references).

In this research, it was discovered, using an in-depth interview technique, that the cultural code of the truck product in the cultural unconscious of the target population is migration. Then, this culture code discovery was supported neurologically by fMRI, a brain-imaging technique.

As a theoretical contribution, the research is an interdisciplinary study, encompassing scientific fields such as psychology, neurology, sociology, general history, as well as specialized fields including art history, genetic algorithms, and software coding languages. Secondly, the research aims to develop an innovative and original method by combining qualitative and quantitative research methods sequentially, and to apply this method to the marketing and neuroscience literature. Thirdly, there might be possible doubts in academic and business environments, since both the discovery of unconscious codes and neuromarketing research are relatively new fields. Using two different methods together will increase reliability by supporting each other. Fourthly, a new example has been added to the neuroscience literature that researchers will benefit from, especially in terms of the task setup and experimental design.

As a practical contribution and managerial implication, the discovery of the unconscious culture code facilitates better segmentation, targeting, and positioning. By shifting the focus from conscious consumer feedback to unconscious cultural anchors, managers can move beyond functional competition and establish a deeper emotional bond with the target audience. The discovery of the unconscious cultural codes has the potential to support the success of international marketing efforts. Given the complexity of national markets from an international company’s perspective, adapting or contingent strategies due to discretionary factors (e.g., cultural dissimilarity) would be necessary (Mandler et al., 2021; Özsomer et al., 2022; Poulis, 2023; Theodosiou and Leonidou, 2003). These strategies would help to solve the common problem for global brands, which is offering the same marketing mix in all national markets. In this sense, the “Migration” code acts as a local lens that global brands can use to personalize their communication without losing their core brand essence. A single psychographic segment or a collective unconscious and archetypal perspective applicable to all countries may lead to ineffective strategies. On the other hand, the cultural code of the same product across national markets may differ from the perspective of the cultural unconscious. At this point, the agile and contingent strategies require the collection of accurate information, including knowledge of the unconscious cultural code embedded in the target market’s social memory. Second, in the next step, knowledge of the cultural code can lead to the design of the marketing mix with precision and efficiency. Recognizing that the customer is not only a driver but also a “migrant,” the product, the messages in promotion, and the places such as showrooms, service areas, fuel stations, and recreational areas can be designed according to the psychology and physical needs of a migrating person. Specifically, recognizing the customer as a “migrant” implies that the product (truck cabin) should be marketed as a “mobile home” that offers psychological security; promotional narratives should emphasize “resilience and the journey” rather than mere horsepower; and places such as showrooms should be designed to evoke the atmosphere of a “safe haven” or a starting point of a significant life event. Third, while common managerial practice often relies on the quick testing of incidental scenes to identify positive reactions, this process is frequently unconcerned with theoretical relevance. We argue that this elaborate development effort is justified because it facilitates a shift from trial-and-error to “Neurological Positioning.” Rapid behavioral tests can identify which image triggers a reaction, but they cannot explain why, often leading to misleading guidance based on fleeting aesthetic appeal. By validating the cultural code through fMRI, manufacturers can mitigate strategic risk, ensuring that their positioning is built on a scientifically verified neural anchor rather than a superficial visual preference. Fourth, knowing the culture code also contributes to the brand strategy; the use of migration code can make a significant difference in strategic brand analysis, brand identity, and brand personality studies (Aaker, 2009). This is an added value that allows the business to price accordingly. This cultural alignment allows brands to implement a “premium pricing” strategy, as the product is no longer perceived as a generic commodity but as a culturally resonant signifier of the driver’s identity. Fifth, as a result of the above-mentioned practical implications, knowledge of the unconscious cultural code may ensure greater investment accuracy and shorter payback times in the automotive industry, which requires very long-term investments and high initial costs. The use of fMRI as a validation tool provides a “risk-mitigation” framework for marketing executives, ensuring that high-budget international campaigns are built on objective neural evidence rather than subjective qualitative interpretations. Finally, these practical results can be extended to all products and social marketing areas (Candan, 2013). If marketing specialists or professionals are familiar with the product’s cultural code, they will have more information about the sociocultural environment, thereby increasing the accuracy and efficiency of their marketing strategies.

### 4.1 Limitations and autocritique

This interdisciplinary research faced several limitations due to its diverse methods, technologies, and the need for various expertise. The self-critique aligns with these limitations. First, culture code discovery is a qualitative research process, which can be criticized for its subjectivity. However, the psychotherapist’s competence, the analysis techniques and collaborative scholarly discussion sessions are sufficient to address this. Second, reaching and securing the participation of truck drivers proved to be difficult. Thus, participants were not chosen via random sampling, which is a potential criticism. Despite this, the participants’ demographic characteristics helped to minimize risks. Third, while this research seeks to explore the cultural unconscious within a broader national context, the empirical focus was geographically localized to Northwest Anatolia. Participants were selected from truck drivers born or residing in this specific region. Although Northwest Anatolia offers a rich cultural microcosm, it is important to acknowledge that regional sub-cultures may exhibit unique nuances that do not fully encapsulate the entire national diversity. Therefore, the findings regarding the “Migration” code should be primarily interpreted within the socio-cultural framework of Northwest Anatolia. Generalizing these results to the entire national society should be done with caution, as other regions might possess distinct cultural anchors influenced by different historical or geographic trajectories. Fourth,. regarding the sample size, while *N* = 33 may be perceived as a limitation for broader generalization, it is consistent with the typical range of task-based fMRI studies in consumer neuroscience. Notably, recent meta-research (Turner et al., 2018) indicates that even significantly larger samples (e.g., *N* = 100) do not guarantee perfect replicability in neuroimaging, highlighting a field-wide challenge rather than a study-specific deficiency. Therefore, our sample provides an adequate statistical basis for identifying the neural correlates of cultural codes while maintaining the necessary scientific caution in our interpretations. Fifth, regarding customer segmentation, the ideal approach would be to form a participant list based on the population rates that affect the product’s purchasing decision-making process in the market. In this case, truck drivers, fleet managers, fleet owners, and purchasing team members of the client company, as well as steering committee members, would be included in the participant list at the same ratio as in the market. However, out of the 22 participants, 14 (64%) were owner-operators, individuals who act as both the user and the primary decision-maker for the vehicle purchase. For the remaining professional drivers, their role is that of a key influencer; in the truck market, the psychological and physical comfort of the driver is often a decisive factor for fleet owners during the purchasing process. Consequently, since the majority of our sample directly holds purchasing authority and the rest represents the most significant influence group, the findings effectively capture the unconscious drivers of the actual market decision-makers. Nevertheless, future research could further refine this segmentation by including other corporate stakeholders such as fleet managers or purchasing officers to differentiate their respective influences. Sixth, the selection of control themes (economy and neutral) could be subject to criticism, as it is theoretically possible that other culturally significant concepts—not tested in this study—could elicit similar neural resonance. However, the strength of this research lies in its tandem methodological design, where the qualitative discovery of Study 1 provides the specific foundation for the neuroimaging validation in Study 2. This alignment between exploratory findings and neural activation provides strong convergent evidence for the “Migration” code, making the results more robust against alternative explanations. While we consider the current control framework sufficient for this initial validation, this point may motivate future researchers to utilize a broader array of cultural codes and control variables to further test the boundaries of this unconscious relational encoding through the falsification principle. Finally, a limitation concerns the distinction between non-conscious processing and conscious awareness. It is plausible, that the “Migration” background benefits from “processing fluency,” leading it to be fluidly recalled by drivers compared to control images. The current design focused on neural patterns of implicit relational encoding and did not include a measure of conscious recall. While the lack of significant prefrontal cognitive appraisal in the fMRI data supports an implicit processing model, we acknowledge that the stimuli may be consciously accessible. Future research should incorporate post-hoc behavioral recall tasks to disentangle the effects of conscious memory from the visceral, non-conscious neural rewards identified in this study.

### 4.2 Future research directions

Suggestions for future research can be summarized as follows: First, as this study was geographically focused on Northwest Anatolia, future research should adopt a multi-regional or nationwide approach to test the consistency of the “Migration” code across Türkiye. Expanding the research universe would allow for a comparative analysis to determine whether this cultural anchor is a universal national phenomenon or if distinct regional sub-cultures assign different symbolic meanings to the same product. Such a broader scale would provide global marketers with more granular data for hyper-local or nationwide targeting strategies. Second, segmentation of participants can be refined in future research to better match purchasing decision-makers, including fleet managers, purchasing team members, and C-level decision-makers, or to differentiate their influences. Third, conducting research on the same product across different societies may yield interesting results. Fourth, further progress toward research reproducibility can be made by incorporating additional control variables. Fifth, there are endless ways to apply the same method to other products. Sixth, it is recommended to apply the same method not only for products but also for social concepts. Seventh, it would be interesting to replicate the unconscious culture code discoveries made by other researchers for other products and concepts, using the methodology employed in this research. Eighth, the product archetypes discovered in other research can be tested using the fMRI model presented in this research. Finally, testing the discovered unconscious culture codes with neuroscientific measurement techniques beyond fMRI may be the subject of future studies.

## Data Availability

The raw data supporting the conclusions of this article will be made available by the authors, without undue reservation, but respecting the limits of the Ethics Committees’ directions.

## Author’s note

This article is derived from the correspondent author’s doctoral thesis completed at Kocaeli University, Turkiye (Çimen, 2021).

## References

[B1] Aaker D. A. (2009). *Güçlü Markalar Yaratmak.* İstanbul: MediaCat. Turkish.

[B2] Adalarasu K. Begum K. G. Priyan M. V. Devendranath C. Sriram G. V. (2025). Neuro-signaling techniques in advertisement endorsements: Unveiling consumer responses and behavioral trends. *J. Retail. Consum. Serv.* 84:104175. 10.1016/j.jretconser.2024.104175

[B3] Adams M. V. (2006). The Islamic cultural unconscious in the dreams of a contemporary muslim man. *J. Jungian Theory Pract.* 8 31–40.

[B4] Alsharif A. H. Wang J. Isa S. M. Salleh N. Z. M. Dawas H. A. Alsharif M. H. (2025). The synergy of neuromarketing and artificial intelligence: A comprehensive literature review in the last decade. *Future Bus. J.* 11:170. 10.1186/s43093-025-00591-x

[B5] Alvino L. Pavone L. Abhishta A. Robben H. (2020). Picking your brains: Where and how neuroscience tools can enhance marketing research. *Front. Neurosci.* 14:577666. 10.3389/fnins.2020.577666 33343279 PMC7744482

[B6] Assmann J. (2008). “Communicative and cultural memory,” in *Cultural Memory Studies*, eds Erll A. Nünning A. (Berlin: Walter de Gruyter), 109–118.

[B7] Assmann J. (2015). *Kültürel Bellek Eski Yüksek Kültürlerde Yazı, Hatırlama ve Politik Kimlik. Translated by A. Tekin.* İstanbul: Ayrıntı. Turkish.

[B8] Bajaj R. Syed A. A. Singh S. (2023). Analysing applications of neuromarketing in efficacy of programmatic advertising. *J. Consum. Behav.* 23 939–958. 10.1002/cb.2249

[B9] Bansal S. Nangia P. Koles B. (2025). Neuromarketing and the marketing mix: An integrative review and future research agenda using the TMC approach. *Int. J. Consum. Stud.* 49:e70072. 10.1111/ijcs.70072

[B10] Bargh J. A. (2021). The Hidden life of the consumer mind. *Consum. Psychol. Rev.* 5 3–18. 10.1002/arcp.1075

[B11] Bargh J. A. Chartrand T. L. (1999). The unbearable automaticity of being. *Am. Psychol. Assoc. Inc.* 54 462–479. 10.1037/0003-066X.54.7.462

[B12] Bechter C. Farinelli G. Daniel R. D. Frey M. (2016). Advertising between archetype and brand personality. *Adm. Sci.* 6 1–11. 10.3390/admsci6020005

[B13] Berne E. (1996). Principles of transactional analysis. *Indian J. Psychiatry* 38 154–159. 21709849 PMC2970834

[B14] Brown R. Kulik J. (1977). Flashbulb memories. *Cognition* 5 73–99. 10.1016/0010-0277(77)90018-X

[B15] Calvert G. A. Brammer M. J. (2012). Predicting consumer behavior: Using novel mind-reading approaches. *IEEE Pulse* 3 38–41. 10.1109/MPUL.2012.2189167 22678839

[B16] Candan B. (2013). “Pazarlamada sosyal sorumluluk ve etik,” in *Pazarlama Yönetimi*, eds Kırcova İ Benli T. (Istanbul: Lisans Yayınları), 445–472. Turkish.

[B17] Casado-Aranda L.-A. Dimoka A. Sánchez-Fernández J. (2021). Looking at the brain: Neural effects of “made in” labeling on product value and choice. *J. Retail. Consum. Serv.* 60:102452. 10.1016/j.jretconser.2021.102452

[B18] Casado-Aranda L.-A. Sánchez-Fernández J. Bigne E. Smidts A. (2023). The application of neuromarketing tools in communication research: A comprehensive review of trends. *Psychol. Mark.* 40 1737–1756. 10.1002/mar.21832

[B19] Cenizo C. (2025). A neuromarketing approach to consumer behavior on web platforms. *Int. J. Consum. Stud.* 49:e70034. 10.1111/ijcs.70034

[B20] Chartrand T. L. Fitzsimons G. J. (2010). Nonconscious consumer psychology. *J. Consum. Psychol.* 21 1–3. 10.1016/j.jcps.2010.12.001d

[B21] Çimen S. (2021). *Pazarlamada kültür kodlarının ve nörobilim deneylerinin kullanımı: Bir ürünün bilinçdışı kültür kodunun keşfi ve nöropazarlama kapsamında fMRI tekniği ile test edilmesi (Publication No. 662527).* doctoral dissertation. Kocaeli: University of Kocaeli. Turkish.

[B22] Cowley E. Anthony C. I. (2019). Deception memory: When will consumers remember their lies? *J. Consum. Res.* 46 180–199. 10.1093/jcr/ucy066

[B23] Dajani K. G. (2022). The social unconscious: Then and now. *Int. J. Appl. Psychoanal. Stud.* 19 179–186. 10.1002/aps.1755

[B24] Duss S. B. Reber T. P. Hänggi J. Schwab S. Wiest R. Müri R. M. et al. (2014). Unconscious relational encoding depends on hippocampus. *Brain* 137 3355–3370. 10.1093/brain/awu270 25273998 PMC4240286

[B25] Evans J. S. B. T. (2008). Dual-processing accounts of reasoning, judgment, and social cognition. *Annu. Rev. Psychol*. 59 255–278. 10.1146/annurev.psych.59.103006.093629 18154502

[B26] Fitzsimons G. Hutchinson J. W. Williams P. Alba J. W. Chartrand T. Huber J. et al. (2002). Non-conscious influences on consumer choice. *Mark. Lett.* 13 269–279. 10.1023/A:1020313710388

[B27] Ford Otosan (2015a). *Ford Cargo rekabet analizi 2004-2015.* Gölcük: Ford Otosan. Turkish.

[B28] Ford Otosan (2015b). *Mercedes Trucks rekabet analizi 2004-2014.* Gölcük: Ford Otosan. Turkish.

[B29] FSL (2020). *FMRIB’s Software Library.* Available online at: www.fmrib.ox.ac.uk/fsl/ (Accessed June 6, 2020)

[B30] Gantt S. P. Agazarian Y. M. (2011). “The group mind, systems-centred functional sub grouping, and interpersonal neurobiology,” in *The Social Unconscious in Persons, Groups and Societies*, eds Hopper E. Weinberg H. (London: Karnac Books), 99–123.

[B31] Göka E. (2008). *Türklerin Psikolojisi.* İstanbul: Timaş. Turkish.

[B32] Gökçe M. Eroğlu E. F. (2017). “Geçmişten günümüze Beşkaza yörüklerinin göç hareketleri ve yolları,” in *Geçmişten Günümüze Göç II*, ed. Köse O. (Karşıyaka: Canik Belediyesi Kültür Yayınları), 801–813.

[B33] Gu M. D. (2023). Cultural unconscious: A theory of cultural criticism. *Eur. Rev.* 32 1–22. 10.1017/S1062798723000339

[B34] Hamelin N. Ferm L.-E. C. Huszar Z. R. Thaichon P. Quach S. (2025). The role of emotions and imagery in financial decision-making: A comparative analysis of neuromarketing and self-report data. *J. Consum. Behav.* 24 2293–2315. 10.1002/cb.2501

[B35] Hannula D. E. Greene A. J. (2012). The hippocampus reevaluated in unconscious learning and memory: At a tipping point? *Front. Hum. Neurosci*. 6:80. 10.3389/fnhum.2012.00080 22518102 PMC3324888

[B36] He L. Freudenreich T. Yu W. Pelowski M. Liu T. (2021). Methodological structure for future consumer neuroscience research. *Psychol. Mark.* 38 1161–1181. 10.1002/mar.21478

[B37] Henderson J. L. (ed.) (1990). “The cultural unconscious,” in *Shadow and Self: Selected Papers in Analytical Psychology*, (Wilmette, IL: Chiron Publications).

[B38] Henke K. Mondadoria C. R. A. Treyer V. Nitsch R. M. Buck A. Hock C. (2003a). Nonconscious formation and reactivation of semantic associations by way of the medial temporal lobe. *Neuropsychologia* 41 863–876. 10.1016/s0028-3932(03)00035-6 12667523

[B39] Henke K. Treyer V. Nagy E. T. Kneifel S. Dürsteler M. Nitsch R. M. et al. (2003b). Active hippocampus during nonconscious memories. *Conscious. Cogn*. 12 31–48. 10.1016/s1053-8100(02)00006-5 12617861

[B40] Jansson-Boyd C. V. Bright P. (2024). “An overview of published articles in consumer neuroscience,” in *Consumer Neuroscience, Theory and Application*, (Amsterdan: Elsevier Science), 211–226. 10.1016/B978-0-443-13581-1.00011-X

[B41] Javor A. Koller M. Lee N. Chamberlain L. Ransmayr G. (2013). Neuromarketing and consumer neuroscience: Contributions to neurology. *BMC Neurol.* 13:13. 10.1186/1471-2377-13-13 23383650 PMC3626833

[B42] Jenkinson M. Bannister P. Brady M. Smith S. (2002). Improved optimisation for the robust and accurate linear registration and motion correction of brain images. *NeuroImage* 17 825–841. 10.1016/s1053-8119(02)91132-8 12377157

[B43] Jung C. G. (1968). *The Archetypes and the Collective Unconscious, The Collected Works of C. G. Jung, vol. 9, issue 2. Translated by R.F.C. Hull.* Princeton: Princeton University Press.

[B44] Jung C. G. (1989). *Memories, Dreams, Reflections. Translated by R. Winston, and C. Winston. Vintage.* Philadelphia: The John C. Winston Company.

[B45] Kajla T. Raj S. Kansra P. Gupta S. L. Singh N. (2023). Neuromarketing and consumer behavior: A bibliometric analysis. *J. Consum. Behav.* 23 959–975. 10.1002/cb.2256

[B46] Kalaganis F. P. Georgiadis K. Oikonomou V. P. Laskaris N. A. Nikolopoulos S. Kompatsiaris I. (2021). Unlocking the subconscious consumer bias: A survey on the past, present, and future of hybrid EEG schemes in neuromarketing. *Front. Neuroergon.* 2:672982. 10.3389/fnrgo.2021.672982 38235255 PMC10790945

[B47] Kesarwani J. Rai H. Kesarwani R. (2025). “A neuromarketing framework for data-driven intelligent automation in marketing,” in *Handbook of Intelligent Automation Systems Using Computer Vision and Artificial Intelligence*, eds Gill R. Hooda S. Srivastava D. Harnal S. 449–469. 10.1002/9781394302734.ch20

[B48] King N. (2012). “Template analysis,” in *Qualitative Organizational Research: Core Methods and Current Challenges*, eds Symon G. Cassell C. (Thousand Oaks, CA: SAGE Publications), 426–450.

[B49] Kümbetoğlu B. (2008). *Sosyolojide ve Antropolojide Niteliksel Yöntem ve Araştırma.* İstanbul: Bağlam Yayıncılık. Turkish.

[B50] Lau H. C. Passingham R. E. (2007). Unconscious activation of the cognitive control system in the human prefrontal cortex. *J. Neurosci.* 27 5805–5811. 10.1523/JNEUROSCI.4335-06.2007 17522324 PMC6672767

[B51] Lloyd S. Woodside A. G. (2013). Animals, archetypes, and advertising (A^3^): The theory and the practice of customer brand symbolism. *J. Mark. Manag.* 29 5–25. 10.1080/0267257X.2013.765498

[B52] Mandler T. Sezen B. Chen J. Özsomer A. (2021). Performance consequences of marketing standardization/adaptation: A systematic literature review and future research agenda. *J. Bus. Res.* 125 416–435. 10.1016/j.jbusres.2020.12.023

[B53] Markowitsch H. J. (2008). “Cultural memory and the neurosciences,” in *Cultural Memory Studies*, eds Erll A. Nünning A. (Berlin: Walter de Gruyter), 275–284.

[B54] Mashrur F. R. Rahman K. M. Miya M. T. I. Vaidyanathan R. Anwar S. F. Sarker F. (2023). Intelligent neuromarketing framework for consumers’ preference prediction from electroencephalography signals and eye tracking. *J. Consum. Behav.* 23 1146–1157. 10.1002/cb.2253

[B55] Maso-Fleischman R. (1997). Archetype research for advertising: A Spanish-language example. *J. Advertising Res.* 37 81–84.

[B56] Mazurek-Łopacińska K. (2016). The cultural paradigm in marketing. *Oeconomia* 15 55–66.

[B57] McLuhan M. (1964). *Understanding Media: The Extensions of Man.* New York, NY: McGraw-Hill.

[B58] McPeek R. W. (2008). The Pearson-Marr archetype indicator and psychological type. *J. Psychol. Type.* 68 52–67.

[B59] Megehee C. M. Woodside A. G. (2010). Creating visual narrative art for decoding stories that consumers and brands tell. *Psychol. Mark.* 27 603–622. 10.1002/mar.20347

[B60] Mlynar J. (2014). Language and collective memory: Insights from social theory. *Slovak J. Polit. Sci.* 14 217–236. 10.1002/mar.20347

[B61] Oliveira P. M. Guerreiro J. Rita P. (2022). Neuroscience research in consumer behavior: A review and future research agenda. *Int. J. Consum. Stud.* 46 2041–2067. 10.1111/ijcs.12800

[B62] Özkara B. Y. Bagozzi R. (2021). The use of event related potentials brain methods in the study of conscious and unconscious consumer decision making processes. *J. Retail. Consum. Serv.* 58:102202. 10.1016/j.jretconser.2020.102202

[B63] Özsomer A. Simonin B. Mandler T. (2022). Marketing agility in subsidiaries: Market orientation and marketing program standardization as the “twin engines” of performance. *J. Int. Mark.* 31:e221130740. 10.1177/1069031X221130740

[B64] Pal S. Das P. Sahu R. Dash S. R. (2021). “Study of neuromarketing with EEG signals and machine learning techniques,” in *Machine Learning for Healthcare Applications*, eds Mohanty S. N. Nalinipriya G. Jena O. P. Sarkar A. (Bristol: IOP Publishing), 10.1002/9781119792611.ch3

[B65] Plassmann H. Ramsøy T. Z. Milosavljevic M. (2012). Branding the brain: A critical review and outlook. *J. Consum. Psychol.* 22 18–36. 10.1016/j.jcps.2011.11.010

[B66] Poulis K. (2023). Standardization and adaptation as a coconstituted process: The pursuit of relational fit in international markets. *J. Int. Mark.* 32:e231212414. 10.1177/1069031X231212414

[B67] Ramsøy T. Z. (2019). Building a foundation for neuromarketing and consumer neuroscience research: How researchers can apply academic rigor to the neuroscientific study of advertising effects. *J. Advertising Res.* 59 281–294. 10.2501/JAR-2019-034

[B68] Reber T. P. Luechinger R. Boesiger P. Henke K. (2012). Unconscious relational inference recruits the hippocampus. *J. Neurosci*. 32 6138–6148. 10.1523/JNEUROSCI.5639-11.2012 22553020 PMC6622124

[B69] Reber T. P. Luechinger R. Boesiger P. Henke K. (2014). Detecting analogies unconsciously. *Front. Behav. Neurosci*. 8:9. 10.3389/fnbeh.2014.00009 24478656 PMC3898596

[B70] Rodríguez V. J. C. Antonovica A. Martín D. L. S. (2023). Consumer neuroscience on branding and packaging: A review and future research agenda. *Int. J. Consum. Stud.* 47 2790–2815. 10.1111/ijcs.12936

[B71] Roesler C. (2012). Are archetypes transmitted more by culture than biology? Questions arising from conceptualizations of the archetype. *J. Anal. Psychol*. 57 223–246. 10.1111/j.1468-5922.2011.01963.x 22444357

[B72] Sánchez-Fernández J. Casado-Aranda L.-A. Bastidas-Manzano A.-B. (2021). Consumer neuroscience techniques in advertising research: A bibliometric citation analysis. *Sustainability* 13:1589. 10.3390/su13031589

[B73] Shiv B. Loewenstein G. Bechara A. Damasio H. Damasio A. R. (2005). Investment behavior and the dark side of emotion. *Psychol. Sci.* 16 435–439. 10.1111/j.0956-7976.2005.01553.x 15943668

[B74] Shteynberg G. Hirsh J. B. Wolf W. Bargh J. A. Boothby E. J. Colman A. M. et al. (2023). Theory of collective mind. *Trends Cogn. Sci.* 27 1019–1031. 10.1016/j.tics.2023.06.009 37532600

[B75] Singer T. Kimbles S. L. (2004). “Introduction,” in *The Cultural Complex: Contemporary Jungian Perspectives on Psyche and Society*, eds Singer T. Kimbles S. L. (New York: Routledge), 1–9. 10.4324/9780203536889

[B76] Singh N. Singh S. Nagaraj S. (2023). Editorial for the special section on neuromarketing in predicting consumer behavior: The efficiency & effectiveness. *J. Consum. Behav.* 23 1597–1601. 10.1002/cb.2285

[B77] Smith S. (2002). Fast robust automated brain extraction. *Hum. Brain Mapp.* 17 143–155. 10.1002/hbm.10062 12391568 PMC6871816

[B78] Snowden R. (2013). *Jung Kilit Fikirler. Translated by K. Atakay.* İstanbul: Optimist. Turkish.

[B79] Solnais C. Andreu-Perez J. Sánchez-Fernández J. Andréu-Abelac J. (2013). The contribution of neuroscience to consumer research: A conceptual framework and empirical review. *J. Econ. Psychol.* 36 68–81. 10.1016/j.joep.2013.02.011

[B80] Talarico J. M. Rubin D. C. (2003). Confidence, not consistency, characterizes flashbulb memories. *Psychol. Sci*. 14 455–461. 10.1111/1467-9280.02453 12930476

[B81] Tambovtsev V. (2015). The myth of the “culture code” in economic research. *Russian J. Econ.* 1 294–312. 10.1016/j.ruje.2015.12.006

[B82] Theodosiou M. Leonidou L. C. (2003). Standardization versus adaptation of international marketing strategy: An integrative assessment of the empirical research. *Int. Bus. Rev.* 12 141–171. 10.1016/S0969-5931(03)00002-3

[B83] Turner B. O. Paul E. J. Miller M. B. Barbey A. K. (2018). Small sample sizes reduce the replicability of task-based fMRI studies. *Commun. Biol.* 1 1–10. 10.1038/s42003-018-0073-z 30271944 PMC6123695

[B84] Vicedo M. (2009). The father of ethology and the foster mother of ducks: Konrad Lorenz as expert on motherhood. *Isis* 100 263–291. 10.1086/599553 19653490

[B85] Vural T. (2017). “Türkülerdeki göç algısı,” in *Geçmişten Günümüze Göç III*, ed. Köse O. (Samsun: Canik Belediyesi Kültür Yayınları), 2003–2008. Turkish.

[B86] Webb E. J. Campbell D. T. Schwartz R. D. Sechrest L. (1966). *Unobtrusive Measures: Nonreactive Research in the Social Sciences.* Chicago, IL: Rand McNally.

[B87] Wegner D. M. (2002). *The Illusion of Conscious Will.* Massachusetts: Bradford Books.

[B88] Welzer H. (2008). “Communicative memory,” in *Cultural Memory Studies*, eds Erll A. Nünning A. (Berlin: Walter de Gruyter), 285–298.

[B89] Wilson T. D. (2002). *Strangers to Ourselves: Discovering the Adaptive Unconscious.* Cambridge, MA: Harvard University Press.

[B90] Woodside A. G. Megehee C. M. Sood S. (2012). Conversations with(in) the collective unconscious by consumers, brands, and relevant others. *J. Bus. Res.* 65 594–602. 10.1016/j.jbusres.2011.02.016

[B91] Woolrich M. W. Ripley B. D. Brady J. M. Smith S. M. (2001). Temporal autocorrelation in univariate linear modelling of fMRI data. *NeuroImage* 14 1370–1386. 10.1006/nimg.2001.0931 11707093

[B92] Wörfel P. (2021). Unravelling the intellectual discourse of implicit consumer cognition: A bibliometric review. *J. Retail. Consum. Serv.* 61:101960. 10.1016/j.jretconser.2019.101960

[B93] Worsley K. J. (2001). “Statistical analysis of activation images,” in *Functional MRI: An Introduction to Methods*, eds Jezzard P. Matthews P. M. Smith S. M. (Oxford: Oxford University Press), 243–250.

[B94] Xu Z. Zhang M. Zhang P. Luo J. Tu M. Lai Y. (2023). The neurophysiological mechanisms underlying brand personality consumer attraction: EEG and GSR evidence. *J. Retail. Consum. Serv.* 73:103296. 10.1016/j.jretconser.2023.103296

[B95] Zaltman G. (2003). *How Customers Think.* Massachusetts: Harvard Business School Press.

[B96] Zhao M. Wang X. (2021). Perception value of product-service systems: Neural effects of service experience and customer knowledge. *J. Retail. Consum. Serv.* 62:102617. 10.1016/j.jretconser.2021.102617

[B97] Zurawicki L. (2010). *Neuromarketing: Exploring the Brain of the Consumer.* Berlin: Springer.

[B98] Züst M. A. Colella P. Reber T. P. Vuilleumier P. Hauf M. Ruch S. et al. (2015). Hippocampus is place of interaction between unconscious and conscious memories. *PLoS One.* 10:e0122459. 10.1371/journal.pone.0122459 25826338 PMC4380440

