# Supplementary material for: Discovery of a product’s unconscious culture code and testing with fMRI
Source: Front Behav Neurosci. 2026 Mar 24;20:1737998. doi: 10.3389/fnbeh.2026.1737998 (PMC13133558; doi:10.3389/fnbeh.2026.1737998)
Supplement: Supplementary file 1 [file Supplementary_file_1.pdf]

Supplementary Material

Discovery of a Product's Unconscious Culture Code and Testing with fMRI

Supplementary Material A: Data Collection Sessions 2

Supplementary Material B: Ethics Committee Approvals 3

Supplementary Material C: Data Collection Forms and Guideline for Study 1 4

Supplementary Material D: Migration in Turkish Culture 10

Supplementary Material E: Comprehensive Review Study 12

Supplementary Material F: Preparation of stimuli (visuals) 19

Supplementary Material G: Selection of Truck Pictures and Preparation of Images 21

Supplementary Material H: Selection of Experiment Background Theme and Visual Preparation 24

Supplementary Material I: Selection of Control Variables and Preparation of Background Images 28

Supplementary Material J: Design Experiment Parameters by Genetic Algorithm 32

Supplementary Material K: fMRI Task Results 49

References 55

Supplementary Material A: Data Collection Sessions

In summary, 14 data collection sessions were conducted. There were 5 participant groups to support 3 studies: Study 1 (in-depth interview), Study 2 (fMRI task), and Study 3 (behavioral test). These data collections were conducted at several locations, and in total, 86 eligible participants participated (Supplementary Table 1). Note: Study 3 was a supplementary study, conducted as the final stage of the research to provide a basis for potential future articles. Therefore, the Study 3 section has been removed from the manuscript and placed in the Supplementary Material.

Supplementary Table 1: The List of The Data Collection Sessions

Data Collection #	Participant Group	Study #	Study Name	Description of Study	Participant Quantity
1	A	1	In-depth interview	In-depth interview	22
2	A	1&2&3	In-depth interview & fMRI & Beh. Test	Demographic Data Collection for Group A	22
3	B	2	fMRI	Pilot Trial: fMRI Task including Product Liking Survey	12
4	B	3	Behavioral Test	Pilot Trial: Product Liking Survey	12
5	B	2	fMRI	Pilot Trial: Demographic Data Collection for Group B	12
6	C	2	fMRI	Design Experiment: Brand Guess Survey with Experts	5
7	D	2	fMRI	Design Experiment: Background Selection Survey	25
8	D	2	fMRI	Design Experiment: Demographic Data Collection Collection for Group D	25
9	-	2	fMRI	Design Experiment: Truck Advertisement Search to Define the Control Variables	-
10	E	2	fMRI	fMRI Task including Product Liking Survey	34
11	E	2	fMRI	Brand Guess Survey	34
12	E	2	fMRI	Background Selection Survey with the Participants	34
13	E	2&3	fMRI & Behavioral Test	Demographic Data Collection Collection for Group E	34
14	E	3	Behavioral Test	Product Liking Survey	34
					86

Supplementary Material B: Ethics Committee Approvals

Ethics Committee approval was received twice from two different universities for the research. The first is for the in-depth interview and pilot study of the neuroscientific tests, and the second is for the main neuroscientific tests.

Kocaeli University Ethics Committee Decision

Ethics Committee approval was received from Kocaeli University for the research's first phase. With this approval, both in-depth interview studies and pilot neuroscientific research studies were conducted. The documents listed below were prepared for the Ethics Committee application and submitted for approval:

- Clinical Research Application Form
- I-2 Document approved by the head of the department or the education officer
- I-3 Clinical Research Protocol
- I-4 Research Flow Chart
- I-5 Participant Information Form (Informed Volunteer Consent Form - BGOF)
- I-10 Research Budget
- I-11 Researcher CV
- P-18 Supporting document/literature regarding the research
- CD containing the documents attached to the I-19 Application file

As a result of the reviews made by the Ethics Committee, it was approved that the research could be conducted with the board's letter

Koç University Ethics Committee Decision

After the in-depth interview and pilot fMRI tests phase of the research, an Ethics Committee application was submitted to Koç University to conduct the actual fMRI and behavioral tests. During this application, the following documents were prepared and submitted for approval:

- Biomedical Clinical Research Ethics Committee Application Form (IRB2)
- Informed Consent Form
- Permission to Use Personal Health Information for Research Purposes
- CV
- Appendix 1: Participant Information Form
- Appendix 2: fMRI Task Participant Responses Record Form
- Appendix 3: Data Collection Form for Visual Stimuli
- Appendix 4: Brand Guess Data Collection Form
- Appendix 5: Short version of images to be shown

After the Ethics Committee's review, approval was obtained via the board's letter, and the work began.

Supplementary Material C: Data Collection Forms and Guideline for Study 1

Participants were asked to complete the Participant Information Form (Supplementary Figure 1) to provide data that would determine their demographic information and eligibility for the test.

Supplementary Figure 1: Participant Information Form

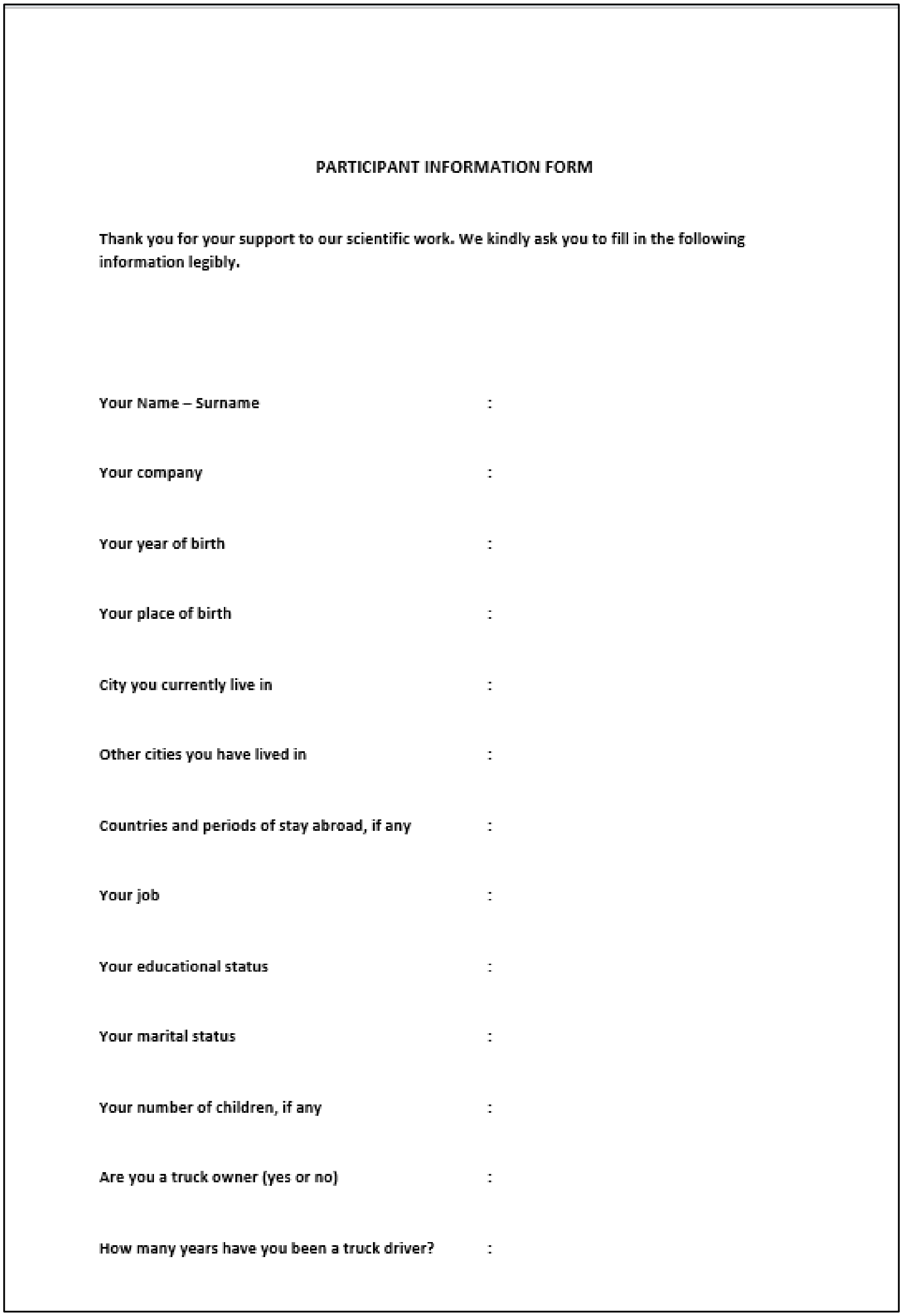

PARTICIPANT INFORMATION FORM

Thank you for your support to our scientific work. We kindly ask you to fill in the following information legibly.

Your Name – Surname	:
Your company	:
Your year of birth	:
Your place of birth	:
City you currently live in	:
Other cities you have lived in	:
Countries and periods of stay abroad, if any	:
Your job	:
Your educational status	:
Your marital status	:
Your number of children, if any	:
Are you a truck owner (yes or no)	:
How many years have you been a truck driver?	:

Supplementary Figure 1: Participant Information Form (continued)

Which of these did you use

- Road truck ☐
- Tow truck ☐
- Construction Truck ☐

Which brand of truck or TIR did you use :

Which routes did you work on and for how long? :

Your mobile phone number where we can reach you :

HEALTH INFORMATION

Have you had any neurological disorders in the past? :

If so, are you currently receiving treatment for such a disease? :

Have you suffered from any psychiatric disorder? :

If so, are you currently receiving treatment for such a disease? :

Do you have any prostheses on your body? :

Do you wear a pacemaker? :

Do you use your right or left hand? :

Do you have any visual impairment? :

Do you use glasses due to vision impairment? :

The answers (liking scores) of the participants to the question on liking were collected while they were in the fMRI task by counting the push buttons, as well as during the behavioral test after the fMRI task. The latter was recorded in a separate form (Supplementary Figure 2).

Supplementary Figure 2: fMRI Task Participants’ Responses Record Form

fMRI Task Participants' Responses Record Form

Name of the Participant: **Date:**

Visual #	Score	Visual #	Score	Visual #	Score
1		25		49	
2		26		50	
3		27		51	
4		28		52	
5		29		53	
6		30		54	
7		31		55	
8		32		56	
9		33		57	
10		34		58	
11		35		59	
12		36		60	
13		37		61	
14		38		62	
15		39		63	
16		40		64	
17		41		65	
18		42		66	
19		43		67	
20		44		68	
21		45		69	
22		46		70	
23		47		71	
24		48		72	

Guideline for Study 1

Archetype Discovery Session with Truck Drivers: Framework Document

This guideline has been prepared by the Authors' Team led by the expert psychologist.

The aim is to structure and design the processes for psychological and in-depth interview sessions.

Interviews will be conducted with 8 people in each group.

Interviews will be held individually first, and then as a group.

Due to the nature of qualitative research, the psychotherapist who conducted the interview can intentionally deviate when appropriate, based on the participant's emotions and thoughts or on the structure and content of the answers.

Before the interviews begin:

1. Participants should be invited to a common area, and an informative speech should be delivered.
2. It should be asked what terminology they use (TIR, truck, tractor-unit), and this terminology should be utilized throughout the research.
3. During the informative speech, the following information and explanatory sentences should be shared verbally: "We are conducting research regarding the vehicle preferences of truck and TIR drivers in Türkiye / about truck drivers. We have reached out to you for this reason. In the research, we will ask you some questions related to the subject and use your answers to reach a conclusion. For this, an individual conversation interview of approximately 30 minutes will be held. Afterwards, a meal will be served. After the meal, you will participate in a collective interview together with a total of 8 of your colleagues. Throughout the meeting, we expect you to answer our questions, listen to other participants without interrupting them while they speak, and actively participate in the study. Your personal information (name) will be kept confidential in this research, and the information we obtain will not be used for purposes other than the research aim we have explained to you. Thank you for your contributions."
4. During the informative speech, there is no need to read the entire Participant Information Form. It should be distributed to participants with a request that they read and sign it.
5. In order to avoid any misinterpretation during the informative speech, Ford brand or the identity of the research team should not be mentioned.
6. Preliminary information should be given stating that a camera and audio recording will be used.
7. After the first individual interviews, the demographic information form should be filled out backstage. The drivers' previous work histories should be obtained through questions: how many years they have been driving, which ones (truck or TIR) they have used, which brands they have used, and on which routes and for how long they have worked.

Questions to be asked in the individual interview:

System 2 / Rational /Addressing the "Adult" Ego

1. Can you describe a truck or TIR to me? What is it? What is it for?
2. What kind of features does the truck you are currently using have? (Detailed explanations should be obtained. In TA terminology, this is a question directed toward the adult ego. It appeals to the rational brain.)
3. According to you, what are the features that should be in a good truck? (Detailed explanations should be obtained. While this question also appeals to the adult ego or rational brain, it gradually begins to descend further into the personal with the phrases "good truck" and "according to you.")

System 2 to System 1 / Emotional / Addressing the "Child", "Parent" Ego

1. Was there anyone in your family who previously worked as a TIR or truck driver?
2. How and why did you start this profession?
3. What kind of prestige and what kind of reputation does using a truck or TIR provide for you?
    - How do you see yourself?
    - How do you guess you appear in the eyes of others?
4. What kind of feeling is it to use a truck or TIR?
    - How do you feel before hitting the road? How do your family and social circle perceive you?
    - How do you feel while behind the wheel?
    - How do you feel when you are alone on a long road with your truck or TIR?
    - How do you feel upon returning home? How do others see you?
    - If I say "being away from home" to you, how would you complete the sentence?
    - If I say "returning home" to you, how would you complete the sentence?
    - If I say "loneliness" to you, how would you complete the sentence?
    - If I say "My vehicle means .... to me," with which words would you complete the sentence?
5. What kind of objects/items of personal importance do you keep in your truck? (Photo, prayer—it should be asked which prayer—writings or sayings—it should be asked what kind of sayings—cover, something given as a gift—it should be asked by whom it was gifted and what the gift was—the music and artist they listen to, etc. Detailed explanations should be obtained. With this question, there is a gradual orientation toward the child's ego. Contact with more primal, more archaic elements begins. Answers may also begin to differentiate personally.)

In Deeper Levels / System 1

6. If I were a 5-year-old child who had never seen a TIR or truck and did not know what it was, how would you describe the truck to me?
7. Can you tell me about your first memory involving trucks or TIRs that you experienced in your childhood? * Who was there? * What had you experienced? * What had you felt?
8. You must have had a toy vehicle/truck in your childhood. * What kind of toy truck was it? * What kind of games would you play? * Where is this truck going? * What is this truck

carrying? * What awaits this truck where it is going? * What does its driver feel at that moment? What are his emotions?

9. If you were to give your truck a name, what name would you give it? What would you say about the meaning of this name? (This is a more suitable question for understanding our culture. Names and their meanings are important for us).
10. If you were to define your truck with an animal name, which animal would your vehicle be?

Questions to be asked in group interviews: ....

General Notes:

- Could the differentiation between individual results and collective results yield a valuable outcome?
  - Due to Türkiye being very heterogeneous from an ethnic perspective,
  - It might be logical that a homogeneous group has been selected.
- Since other countries have more homogeneous ethnic structures, this comment can be added to the study.
- By going in reverse, if cultural code and archetype discovery cannot be performed even in a homogeneous group, then one should turn to other areas, especially in terms of segmentation and positioning.
- Individual interviews will be held before the meal, and the group study will be held after the meal.
- There should be a maximum of 3 people in the room during the individual interview, including the participant.
- The camera should be placed in a fixed and inconspicuous manner.
- For the method, respectively, one should descend from System 2 to System 1, from conscious to unconscious. After reaching System 1, distinguishing the source of the answers, such as individual, cultural or collective unconscious, should be assessed during the analysis stage.
- For the validity and reliability study, the number of participants can be increased, and different analysts can be requested to resolve the same data.
- It would be beneficial to provide a literature summary of archetype discovery to the analysts beforehand.

Physical elements that will be needed in the interviews:

- A carpeted hall
- Three different arrangements: theater arrangement for the opening speech, a 3-seat and coffee table arrangement for individual interviews, and a comfortable chair or armchair arrangement in a round layout for the group interview.
- The light should be adjustable.
- There should be a music system.
- It should be possible to make camera and audio recordings.
- There should be comfortable armchairs.
- There should be one small round table.
- There should be one large table.
- Plentiful paper in different sizes.
- Plentiful and colorful felt-tip drawing pens (markers).
- Treats in the common area (there might not be coffee service).

Supplementary Material D: Migration in Turkish Culture

Migration is defined as the movement of individuals or groups from one country or settlement to another, driven by economic, social, and political factors (Çimen, 2021).

The migrations that Turkish population have experienced throughout their history can be divided into three main categories (Avcıoğlu, 1978; Kafesoğlu, 1977; Ögel, 1984; Roux, 2007; Roxburgh, 2005; Togan):

- great mass migrations: climate change and natural disasters, quantitative or qualitative insufficiency of pastures, military conquests, expanding the territories of the state, being pushed out of their current homeland due to wars and exiles, withdrawal due to conquests, and resettlement policies of states.
- nomadism (seasonally moving to spring, winter or autumn areas for economic subsistence),
- commercial mobility (short distance or long distance, including Silk Road Trade, regular inter-country travel)

The migration phenomenon can be observed in various cultural elements; the more effectively this phenomenon is integrated into social culture and the social unconscious. It is possible to see the traces of the concept of migration and its impact:

- in literary, folk literature, mythological stories, epics, fairy tales (Vural, 2017),
- in folk songs, folk poems, and minstrel literature (Türkan and Arı, 2018),
- in modern literature (Satık, 2018),
- in cinema (Osmanoğlu, 2016),
- in theatre (Sevim, 2007),
- in architecture (Erkaya, 2016),
- in music (Erbay, 2016) and
- in painting (Üner, 2018).

Supplementary Table 2: Codes and Subgroups Data Table

Code	Description	Subgroups (Potential Culture Codes): Home	Freedom	Migration	Ego satisfaction
Code 1	Driving a huge machine, powerful car, sense of greatness				x
Code 2	Comfort	x			
Code 3	Not everyone can do it, rarity, uniqueness, reputation, prestige, pride				x
Code 4	Cleanliness	x			
Code 5	Living space	x		x	
Code 6	Ostentation, beauty, aesthetics				x
Code 7	Dangerous journey, uncertainty, excitement			x	
Code 8	Safety anxiety, stress			x	
Code 9	Help, solidarity, support in bad times, acquiring an environment			x	
Code 10	Coping with difficult conditions, succeeding, enjoying it			x	x
Code 11	Life on the road is beautiful, enjoyable, fun		x	x	
Code 12	Geographical freedom, traveling, resting		x	x	
Code 13	Freedom to not account to anyone, it's hard to deal with people		x	x	x
Code 14	Being natural, being straightforward		x		
Code 15	Reaction against Authority, State, NGOs			x	
Code 16	Oral culture, conversation			x	
Code 17	Disrespect, belittlement, contempt			x	
Code 19	Become a wise man; have a lot of memories			x	x
Code 20a	Patience, hard work, endurance, ambition			x	x
Code 20b	Protection: Family, burden, trust, asset	x		x	
Code 21	Freedom from family, father, puberty		x		x
Code 22	Loneliness, sadness, longing, suffering			x	
Code 23	Comrade, friend, confidant, companion, personification			x	
Code 24	Fear as a child				
Code 25	A passion for cars from a young age				
Code 26	Accommodation place	x		x	
Code 27	Fondness for innovation, fondness for firsts, technology			x	x
Code 28	Conflict with the settlers		x	x	
Code 29	Common property, kin, cooperation, bond, community			x	
Code 30	Trucking with family or friends	x		x	
Code 31	Respect for the law, compliance with the rules.			x	
Code 32	Upgrade, class difference, symbolizing it with property			x	x
Code 33	The joy of returning home, peace, confidence	x			

Code	Day 1: Participant 1	Participant 2	Participant 3	Participant 4	Participant 5	Participant 6	Participant 7	Participant 8	Rest and Painting	Closing Comment	SUBTOTAL	
Code 1	1	1	1		1	1		1	1		6	
Code 2	1	1		1				1			4	
Code 3	1	1		1	1	1	1			1	6	
Code 4	1		1					1			3	
Code 5	1	1	1	1			1	1	1	1	6	
Code 6	1	1						1			3	
Code 7	1										1	
Code 8	1			1	1	1				1	4	
Code 9						1		1			2	
Code 10		1		1	1	1					4	
Code 11									1		0	
Code 12		1			1				1		2	
Code 13		1						1			2	
Code 14											0	
Code 15											0	
Code 16											0	
Code 17		1		1							2	
Code 19										1	0	
Code 20a											0	
Code 20b		1									1	
Code 21		1	1		1	1	1	1		1	6	
Code 22		1	1	1				1		1	4	
Code 23					1	1	1			1	3	
Code 24						1					1	
Code 25											0	
Code 26											0	
Code 27											0	
Code 28											0	
Code 29											0	
Code 30											0	
Code 31											0	
Code 32											0	
Code 33											0	
Total											82	100%
Home											14	17%
Freedom											10	12%
Migration											31	38%
Ego Satisfaction											27	33%

Code	Day 2: Participant 1	Participant 2	Participant 3	Participant 4	Participant 5	Participant 6	Participant 7	Participant 8	Participant 9	Group	Teachers' Commen	SUBTOTAL	
Code 1	1	1	1	1	1	1	1		1	1		8	
Code 2	1		1	1			1	1				5	
Code 3	1	1		1		1	1	1	1	1	1	7	
Code 4									1			1	
Code 5	1	1	1	1			1		1		1	6	
Code 6	1											1	
Code 7						1	1		1		1	3	
Code 8		1	1	1	1			1	1			6	
Code 9	1			1	1	1	1		1			6	
Code 10			1	1		1	1					4	
Code 11	1	1		1		1	1		1			6	
Code 12	1	1	1	1		1	1	1	1		1	8	
Code 13		1		1		1		1	1	1		5	
Code 14								1				1	
Code 15		1	1	1	1	1		1	1	1	1	7	
Code 16	1											1	
Code 17	1	1	1	1	1		1	1	1	1	1	8	
Code 19			1	1		1	1	1	1			6	
Code 20a				1		1		1				3	
Code 20b	1	1	1	1					1	1		5	
Code 21	1	1		1		1	1			1	1	5	
Code 22	1		1	1			1	1	1			6	
Code 23		1		1			1		1			4	
Code 24									1			1	
Code 25						1	1		1	1		3	
Code 26						1						1	
Code 27	1							1				2	
Code 28			1						1			2	
Code 29					1		1					2	
Code 30						1		1				2	
Code 31								1	1			2	
Code 32							1					1	
Code 33						1						1	
Total												186	100%
Home												21	11%
Freedom												27	15%
Migration												96	52%
Ego Satisfaction												42	23%

Code	Day 3: Participant 1	Participant 2	Participant 3	Participant 4	Participant 5	SUBTOTAL		TOTAL	
Code 1			1		1	2		16	
Code 2			1			1		10	
Code 3		1	1	1		3		16	
Code 4	1		1		1	3		7	
Code 5	1		1		1	3		15	
Code 6	1		1			2		6	
Code 7			1			1		5	
Code 8		1	1		1	3		13	
Code 9						0		8	
Code 10			1		1	2		10	
Code 11		1	1			2		8	
Code 12	1	1	1	1	1	5		15	
Code 13		1	1	1		3		10	
Code 14						0		1	
Code 15	1		1	1		3		10	
Code 16						0		1	
Code 17	1		1		1	3		13	
Code 19			1			1		7	
Code 20a			1			1		4	
Code 20b						0		6	
Code 21			1	1	1	3		14	
Code 22	1		1	1	1	4		14	
Code 23			1		1	2		9	
Code 24						0		2	
Code 25	1		1		1	3		6	
Code 26	1	1				2		3	
Code 27				1		1		3	
Code 28						0		2	
Code 29						0		2	
Code 30						0		2	
Code 31						0		2	
Code 32						0		1	
Code 33						0		1	
Total						76	100%	344	100%
Home						9	12%	44	13%
Freedom						13	17%	50	15%
Migration						36	47%	163	47%
Ego Satisfaction						18	24%	87	25%

Supplementary Material E: Comprehensive Review Study

The neuromarketing and fMRI literature was scanned in a "comprehensive review" format to clarify the research model and test its hypothesis. Scanning results are recorded in Supplementary Table 3. The criteria taken into consideration during this scanning and creating the table are as follows:

- "fMRI" or "unconscious" were used as two main keywords. In combination with these, the words "brand recognition", "economy", "emotion", "culture", "long-term memory", "memory", "motivation and reward", "trauma" and "metaphor" were used. The articles found are marked in the relevant columns of whichever of these words they are related to.
- The brain regions mentioned in the articles about the unconscious were recorded in the "Unconscious ROI (Region of Interest)" column. Brain regions related to other keywords were recorded in the "Other Brain Region" column.
- Articles are categorized according to the method used in scientific studies. These categories are motor movements (motor), visual stimuli (visual), tests with words (lexical), tests of sound and hearing (auditory-phonographic), and semantic tests (semantic). A design using visual stimuli was preferred in this study.
- Along with these, the following topics on which the research focuses are also recorded in the same column: physiological, face recognition, touch, emotion, preference, and decision.
- There is diversity depending on whether the stimuli used in scientific studies identified in the literature are unconscious or conscious, and whether the unconscious ones are subliminal or supraliminal. In some studies, the same stimulus was used both at the unconscious and conscious levels. Accordingly, articles were coded into three different categories: subliminal, supraliminal unconscious, and supraliminal conscious. Subliminal stimuli are stimuli to which a person is exposed through the senses but cannot perceive at the conscious level (Quoted by Brooks et al., 2012). For example, during the perception period, when the person sees the images below, the visual regions of the brain are active, but the person is not aware of seeing them. Similarly, subliminal stimuli can be understood as auditory stimuli (Stephan et al. 2002, p. 345). Subliminal stimuli can be created through exposure to SOA (Stimulus Onset Asynchrony) periods of 50 ms or less and are generally used in conjunction with the masking procedure (Brooks et al., 2012; Meneguzzo et al.,2014; Ruch, Herbert, and Henke, 2017). In supraliminal stimuli, the stimulus is above the limits of conscious perception, so the person can become aware of it if they pay attention or focus (Meneguzzo et al., 2014). Here, when a stimulus is supraliminal, it can be perceived at either the conscious or unconscious level. When people pay attention to a supraliminal stimulus, they become aware of it. This is referred to as supraliminal conscious stimulus or supraliminal conscious level perception state. If a person does not pay attention to a supraliminal stimulus —for example, a visual stimulus —the person sees it but does not realize it. This phenomenon is referred to as supraliminal unconscious stimulus or supraliminal unconscious perception state (Ran et al., 2016; Kouider and Dehaene, 2007). In scientific studies, these three stimulus types are used for encoding and recall. In some studies, both were used. If at least two of them are used in a study, it becomes important whether the same stimulus or different stimuli are used during encoding and retrieval. If the same stimulus was used, it was distinguished and recorded in the relevant column of the table.
- In studies in literature, activation levels of relevant brain regions (ROI - Region of Interest) are measured when stimuli are applied at the unconscious or conscious level. Accordingly, four different types of activation can be observed: Regions activated only by unconscious

stimuli. These are marked in the table as “unconscious”. Regions where unconscious activation is greater than conscious level activation. These are marked “U > C” in the table. Regions where unconscious activation is less than conscious level activation. These are marked “U < C” in the table. Regions where there is no difference between unconscious activation and conscious level activation. These are marked “U = C” in the table.

- Considering the hypotheses and models in the literature, 4 different models were considered to test the hypothesis in this thesis study. These models are coded as A, B, C and D in the table.

The models categorized in the systematic review study and coded as A, B, C, and D are discussed in detail. For each of these, arguments have been put forward for their selection as models; on the other hand, counterarguments that weaken their suitability as models have also been discussed. Ultimately, given these discussions, model D was chosen for this research.

Supplementary Table 3: Comprehensive Review Study

	Reference	Unconscious	Brand Recognition	Economy	Emotion	Culture	Long-term Memory	Memory	Motivation and Award	Trauma	Metaphor	Unconscious ROI-Region of Interest	Other Brain Region	Motor<br>Visual<br>Lexical<br>Audio<br>Semantic<br>Physiologic<br>Face Recognition<br>Touch<br>Emotion<br>Preference<br>Decision	Type of Stimuli:<br>1. Subliminal<br>2. Supraliminal Unconscious<br>3. Supraliminal Conscious<br>4. Both	If "Both":<br>Are the conscious and unconscious stimuli the same?	Relevant ROI Activation:<br>1. Unconscious only<br>2. U>C<br>3. U<C<br>4. U=C<br>5. Note for the compatibility<br><br>U: Unconscious Activation<br>C: Conscious Activation	Supported Method
1	Morris, J.S., A. Öhman, R.J. Dolan (1998). "Conscious and unconscious emotional learning in the human amygdala". Nature 393: 467-470	x			x							right amygdala		Visual, Face Recognition, Emotion	Subliminal and Supraliminal Conscious	Yes	U>C	A, B
2	Teuchies, Martyn (2017). Interactions of conscious and unconscious processes in decision making. Doktora Tezi, Ghent University Faculty of Psychology and Educational Sciences, Ghent.	x										rostral cingulate zone (rcz)<br>anterior insula<br>the supramarginal gyrus		Visual, Preference	Subliminal			B
3	Brooks, S.J., V. Savov, E. Allzén, vd (2012). "Exposure to subliminal arousing stimuli induces robust activation in the amygdala, hippocampus, anterior cingulate, insular cortex and primary visual cortex: A systematic meta-analysis of fMRI studies". NeuroImage, 59: 2962–2973	x			x			x				right amygdala<br>hippocampus<br>bilateral anterior cingulate cortex<br>insular cortex<br>primary visual cortex<br>fusiform		Physiologic, Face Recognition, Lexical, Audio	Subliminal			A, B
4	Dehaene, Stanislas, Lionel Naccache, Gurvan Le Clec'H, vd (1998). "Imaging unconscious semantic priming". Nature, 9: 597-600	x										amygdala						A, B
5	Ran, Guangming, Xu Chen, Xiaojun Cao, Qi Zhang (2016). "Prediction and unconscious attention operate synergistically to facilitate stimulus processing: An fMRI study". Consciousness and Cognition 44: 41–50.	x			x							left dorsolateral prefrontal cortex (dlpfc)		Visual	Subliminal and Supraliminal Conscious	Yes		B
6	Wimmer, G. Elliott, Daphna Shohamy, (2012). "Preference by Association: How Memory Mechanisms in the Hippocampus Bias Decisions". Science, 338 (6104): 270-273	x		x				x				hippocampus		Visual, Decision	Supraliminal Unconscious			A, B
7	Schaefer Michael, Georg Northoff, (2017). "Who Am I: The Conscious and the Unconscious Self". Frontiers in Human Neuroscience, 11(126): 1-5	x									x	primary somatosensory cortex		Motor, Touch				
8	Lacey, Simon, Randall Stilla, K. Sathian (2013). "Metaphorically Feeling: Comprehending Textural Metaphors Activates Somatosensory Cortex". Brain Lang 120 (3): 416-421	x									x	primary somatosensory cortex in parietal operculum		Motor, Touch	Supraliminal Unconscious			
9	Denke, Claudia, Michael Rotte, Hans-Jochen Heinze vd (2016). "Lying and the Subsequent Desire for Toothpaste: Activity in the Somatosensory Cortex Predicts Embodiment of the Moral-Purity Metaphor". Cerebral Cortex, 26: 477–484	x				x					x	primary somatosensory cortex<br>left sensorimotor activation<br>middle/ superior temporal gyrus<br>amygdala<br>hippocampus		Motor, Semantic	Supraliminal Unconscious			
10	Ferguson Melissa J. (2007). "The Automaticity of Evaluation". Şu kitapta: Ed. Arie W. Kruglanski, Joseph P. Forgas, Social Psychology and the Unconscious. New York: Psychology Press: 219-264	x			x							amygdala<br>right inferior prefrontal cortex						A
11	Bode, Stefan, Anna Hanxi He, Chun Siong Soon, vd (2011). "Tracking the Unconscious Generation of Free Decisions Using Ultra-High Field fMRI" Plos One, 6 (6) : 1-13	x										anterior frontopolar cortex		Visual, Lexical	Supraliminal Conscious			B
12	Squire, Larry R., Adam J.O. Dede, (2015). "Conscious and Unconscious Memory Systems". Cold Spring Harbor Laboratory Press, 7: 1-15	x			x		x	x				hippocampus<br>amygdala						A, B
13	Badiyan, Rajendra D. (2005). "Conscious awareness of retrieval: an exploration of the cortical connectivity". International Journal of Psychophysiology, 55: 257-262	x						x				extrastriate cortex						A
14	Lidaka, Tetsuya, Tokiko Harada (2016). "Cultural Values Modulate Emotional Processing in Human Amygdala". Şu kitapta: Ed. Peter e. Nathan, Joan Y. Chiao, Shu-Chen Li, Rebecca Seligman, Robert Turner. The Oxford Handbook of Cultural Neuroscience. Oxford: Oxford University Press: 107-120				x	x							amygdala	Visual	Supraliminal Unconscious			A, B
15	Phan, K.Luan, Tor Wager, Stephan F.Taylor, vd (2002). "Functional Neuroanatomy of Emotion: A Meta-Analysis of Emotion Activation Studies in PET and fMRI". NeuroImage, 16 (2): 331-348	x			x			x					medial prefrontal cortex<br>amygdala<br>subcallosal cingulate<br>occipital cortex<br>anterior cingulate<br>insula					A, B
16	Greene, Anthony J., William L. Gross, Catherine L. Elsing, vd (2007). "Hippocampal differentiation without recognition: An fMRI analysis of the contextual cueing task". Learn. Mem 14: 548-553	x						x				hippocampus		Visual	Supraliminal Unconscious			A, B
17	Inagaki, Kyota, Keiichi Onoda, Shuhei Yamaguchi (2014). "An fMRI study of unconscious emotional processing in cerebellum" International Journal of Psychophysiology 94: 247	x			x							the left cerebellum and the left inferior frontal gyrus		Visual, Face Recognition, Emotion	Subliminal and Supraliminal Conscious	Yes	U>C	A, B
18	Züst, Marc Alain, Patrizio Colella, Thomas Peter Reber, vd (2015). "Hippocampus Is Place of Interaction between Unconscious and Conscious Memories". Plos One: 1-19	x						x				hippocampus<br>hippocampal<br>angular gyrus		Visual, Face Recognition, Semantic	Subliminal and Supraliminal Conscious	Yes	Hippocampal activation decreases if compatible	A, D
19	Degonda, Nadia, Christian R.A. Mondadori, Simone Bosshardt, vd (2005) "Implicit Associative Learning Engages the Hippocampus and Interacts with Explicit Associative Learning". Neuron 46 : 505-520	x						x				hippocampus<br>parahippocampal gyrus		Visual, Face Recognition	Subliminal and Supraliminal Conscious	Yes		A, D
20	Henke, Katharina, Christian R.A. Mondadoria, Valerie Treyer vd (2003). "Nonconscious formation and reactivation of semantic associations by way of the medial temporal lobe". Neuropsychologia 41: 863–876	x						x				hippocampus<br>perirhinal cortex<br>medial temporal lobe<br>parahippocampal cortex/gyrus<br>angular gyrus<br>precuneus		Visual, Face Recognition, Semantic	Subliminal and Supraliminal Conscious	Yes		A, D
21	Reber, Roger, Thomas P., Luechinger, Peter Boesiger, vd (2012). "Unconscious Relational Inference Recruits the Hippocampus". The Journal of Neuroscience 32(18):6138–6148	x						x				hippocampus<br>medial temporal lobe<br>parahippocampal<br>posterior cingulate<br>fusiform<br>middle temporal gyrus		Visual, Face Recognition, Semantic	Subliminal and Supraliminal Conscious	Yes		A, D

Supplementary Table 3: Comprehensive Review Study (continued)

	Reference	Unconscious	Brand Recognition	Economy	Emotion	Culture	Long-term Memory	Memory	Motivation and Award	Trauma	Metaphor	Unconscious ROI-Region of Interest	Other Brain Region	Motor Visual Lexical Audio Semantic Physiologic Face Recognition Touch Emotion Preference Decision	Type of Stimuli: 1. Subliminal 2. Supraliminal Unconscious 3. Supraliminal Conscious 4. Both	If "Both": Are the conscious and unconscious stimuli the same?	Relevant ROI Activation: 1. Unconscious only 2. U>C 3. U<C 4. U=C 5. Note for the compatibility U: Unconscious Activation C: Conscious Activation	Supported Method
22a		x										subcortical (presumably basal ganglia–thalamic) control circuits corticostriate circuits (involving subcortical structures like the thalamus and caudate nucleus, and perhaps posterior parietal cortex		Motor	Subliminal and Supraliminal Conscious	Yes		B
22b	Berlin, Heather A. (2011). "The Neural Basis of the Dynamic Unconscious". 13 (1) : 5-31	x										subcortical visual pathway that includes the superior colliculus, pulvinar thalamus, and amygdala (aka a subcortical retino–tecto–thalamic route to the amygdala)		Visual				
22c		x										(in the monkey) the thalamic lateral geniculate nucleus (lgn)		Visual				
22d		x										striatum somatosensory association areas amygdala fusiform gyrus and superior temporal sulcus						
23	Smith, Rachelle, Kamyar Keramatian, Jonathan Smallwood, vd (2006). "Mind-wandering with and without Awareness: An fMRI study of spontaneous thought processes". Ed. R. Sun. 28th Annual Conference of the Cognitive Science Society. Vancouver: Curran Associates, Inc. : 804-809.	x					x	x				lateral temporal cortex anterior temporal and lateral mid-temporal regions		Lexical, Semantic			U>C	A, B
24	Creswell, John David, James K. Bursley, Ajay B. Satpute (2013). "Neural reactivation links unconscious thought to decision-making performance". SCAN, 8: 863 -869	x										right dorsolateral prefrontal cortex and left intermediate visual cortex		Lexical, Semantic	Supraliminal Unconscious ve Supraliminal Conscious	Yes	U=C	B
25	Vuilleumier, P., J.L. Armony, K. Clarke (2002). "Neural response to emotional Yüz Tanımas with and without awareness: event-related fMRI in a parietal patient with visual extinction and spatial neglect". Neuropsychologia 40: 2156–2166	x			x							amygdala, orbifrontal cortex		Visual, Face Recognition, Emotion	Subliminal and Supraliminal Conscious	Yes	U=C	A, B
26	Killgore, William D.S., Deborah A. Yurgelun-Todd (2004). "Activation of the amygdala and anterior cingulate during nonconscious processing of sad versus happy Yüz Tanımas". NeuroImage 21 : 1215–1223	x			x							amygdala and anterior cingulate gyrus		Visual, Face Recognition, Emotion	Subliminal			A
27	Kensinger, Elizabeth A (2008). "Neuroimaging the formation and retrieval of emotional memories". Şu kitapta: Ed. F. Columbus. Brain Mapping. New York, Hauppauge: Nova Science Publishers, Inc.	x			x			x				amygdala hippocampus						A
28	Hannula, Deborah E. , Anthony J. Greene (2012). "The hippocampus reevaluated in unconscious learning and memory: at a tipping point?". Frontiers in Human Neuroscience 6(80) : 1-20	x					x	x				hippocampus						A
29	Chaumon, Maximilien, Dominique Hasboun, Michel Baulac vd. (2009). "Unconscious contextual memory affects early responses in the anterior temporal lobe". Brain Research 1285 77-87	x										anterior temporal cortex		Visual				
30	Soon, Chun Siong, Marcel Brass, Hans-Jochen Heinz, John-Dylan Haynes (2008). "Unconscious determinants of free decisions in the human brain". Nature Neuroscience 11: 543–545.	x										primary motor cortex sma (supplementary motor area) precuneus frontopolar cortex		Motor	Supraliminal Conscious			
31	Duss, Simone B., Thomas P. Reber, Jürgen Hanggi vd (2014). "Unconscious relational encoding depends on hippocampus". Brain 1 : 2-16	x					x	x				hippocampus hippocampal- anterior thalamic axis and its connections to neocortex medial temporal lobe and neocortex parahippocampal gyrus postcentral gyrus, posterior cingulate, lingual gyrus, fusiform, middle temporal gyrus		Lexical, Semantic	Subliminal and Supraliminal Conscious	Yes	U<C	A, B, D
32	Stephan, K. M., M. H. Thaut, G. Wunderlich vd (2002). "Conscious and Subconscious Sensorimotor Synchronization—Prefrontal Cortex and the Influence of Awareness". NeuroImage 15, 345–352	x										ventral medial frontal areas (for motor adaptations performed subconsciously)		Motor, Audio	Subliminal and Supraliminal Conscious	Yes	Unique to unconscious	B
33a		x										anterior cingulate cortex (acc)					U=C	
33b	Meneguzzo, Paolo, Manos Tsakiris, Helgi B Schioth, vd (2014). "Subliminal versus supraliminal stimuli activate neural responses in anterior cingulate cortex, fusiform gyrus and insula: a meta-analysis of fMRI studies". Meneguzzo et al. BMC Psychology 2:52	x										right fusiform gyrus (part of middle occipital gyrus) and right insula (right posterior insula cortex)		Visual, Audio	Subliminal, Supraliminal Conscious Supraliminal Unconscious	Yes	U>C	B
33c		x										right hemisphere					U>C	
33d		x										left hemisphere					U<C	
33e		x										left rostral anterior cingulate					U<C	
34a	Zurawicki, Leon (2010). Neuromarketing Exploring the Brain of the Consumer. New York : Springer	x			x		x	x				ventromedial prefrontal cortex (vmpfc) amygdala in the limbic system pfc medial temporal lobe						
34b		x										amygdala						

Supplementary Table 3: Comprehensive Review Study (continued)

	Reference	Unconscious	Brand Recognition	Economy	Emotion	Culture	Long-term Memory	Memory	Motivation and Award	Trauma	Metaphor	Unconscious ROI-Region of Interest	Other Brain Region	Motor Visual Lexical Audio Semantic Physiologic Face Recognition Touch Emotion Preference Decision	Type of Stimuli:<br>1. Subliminal<br>2. Supraliminal Unconscious<br>3. Supraliminal Conscious<br>4. Both	If "Both": Are the conscious and unconscious stimuli the same?	Relevant ROI Activation:<br>1. Unconscious only<br>2. U>C<br>3. U<C<br>4. U=C<br>5. Note for the compatibility<br><br>U: Unconscious Activation<br>C: Conscious Activation	Supported Method
35	Hunt, Harry T. (2012). "A collective unconscious reconsidered: Jung's archetypal imagination in the light of contemporary psychology and social science". Journal of Analytical Psychology, 57 : 76–98	x										phylogenetically primitive subcortex, including both limbic areas and the thalamic-reticular system of the upper brain stem						
36	Cunningham, William A., Marcia K. Johnson, Carol L. Raye, vd (2004). "Separable Neural Components in the Processing of Black and White Yüz Tanımas". American Psychological Society 15(12): 806-813	x			x	x						amygdala		Visual, Face Recognition, Emotion	Subliminal and Supraliminal Conscious	Yes	U>C	A, B
37	Cunningham, William A., Carol L. Raye, Marcia K. Johnson (2004). "Implicit and Explicit Evaluation: fMRI Correlates of Valence, Emotional Intensity, and Control in the Processing of Attitudes". Journal of Cognitive Neuroscience 16 (10): 1717–1729	x			x							amygdala, right insula		Lexical, Semantic	Supraliminal Unconscious ve Supraliminal Conscious	Yes	U>C	A, B
38	Cunningham, William A., Marcia K. Johnson, Mahzarin R. Banaji, vd (2003). "Neural Components of Social Evaluation". Journal of Personality and Social Psychology 85(4) : 639-649	x										amygdala		Lexical, Semantic	Supraliminal Unconscious ve Supraliminal Conscious	Yes	U>C	A, B
39	Cunningham, William A., Philip David Zelazo (2007). "Attitudes and evaluations: a social cognitive neuroscience perspective". Trends in Cognitive Sciences 11 (3)	x										bilateral amygdala, orbitofrontal cortex (ofc) and right insula, brain stem		Lexical, Semantic	Supraliminal Unconscious ve Supraliminal Conscious	Yes	U>C	A, B
40a	Kouider, Sid, Stanislas Dehaene (2007). "Levels of processing during non-conscious perception: a critical review of visual masking" Philosophical Transactions of the Royal Society B 362 : 857-875	x										bilateral intraparietal cortices		Lexical, Semantic				
40b		x										the left middle temporal gyrus		Lexical, Semantic				
40c		x			x							amygdala		Visual, Face Recognition, Emotion				
40d		x			x							amygdala		Emotion				
41	Lau, Hakwan C., Richard E. Passingham (2007). "Unconscious Activation of the Cognitive Control System in the Human Prefrontal Cortex" The Journal of Neuroscience, 27 (21) : 5805-5811	x										mid-dorsolateral prefrontal cortex		Lexical, Audio, Semantic	Subliminal		If incompatible, activation in U increases, and activation in C decreases	D
42	Tusche, Anita, Thorsten Kahnt, David Wisniewski, vd (2013). "Automatic processing of political preferences in the human brain". NeuroImage 72 : 174-182	x										ventral striatum<br>insula<br>cingulate cortex		Visual	Supraliminal Unconscious			
43	Mostafa, Mohamed M. (2012). "Brain processing of vocal sounds in advertising: A functional magnetic resonance imaging (fMRI) study". Expert Systems with Applications 39 : 12114–12122		x										medial prefrontal cortex	Audio	Supraliminal Conscious			
44	Phua, D.Y. , G.I. Christopoulos (2014). "Social Neuroscience Tasks: Employing fMRI to Understand the Social Mind". InTech - Open Science Open Minds 25 : 653-678	x			x	x						amygdala						A, B
45a	Plassmann, Hilke, Thomas Zoëga Ramsøy, Milica Milosavljevic (2012). "Branding the brain: A critical review and outlook". Journal of Consumer Psychology 22(1) : 18-36	x	x		x	x		x	x			ventral striatum						A
45b	Plassmann, Hilke, Thomas Zoëga Ramsøy, Milica Milosavljevic (2012). "Branding the brain: A critical review and outlook". Journal of Consumer Psychology 22(1) : 18-36		x										dorsolateral prefrontal cortex					
45c			x										hippocampus and dorsolateral prefrontal cortex					
45d			x										superior frontal gyrus of the pfc					
45e			x										dlpfc, hippocampus, and parahippocampal cortex.					
45f			x										striatum					
46	Pessiglione, Mathias, Liane Schmidt, Bogdan Draganski vd (2007). "How the Brain Translates Money into Force: A Neuroimaging Study of Subliminal Motivation". Science 316 : 904 – 906	x							x			basal forebrain region<br>ventral pallidum		Motor, Visual, Semantic	Subliminal and Supraliminal Conscious	Yes	U<C	B
47	Pessiglione, Mathias, Predrag Petrovic, Jean Daunizeau, vd (2008). "Subliminal Instrumental Conditioning Demonstrated in the Human Brain" Neuron 59 : 561–567	x							x			ventral striatum		Visual, Semantic	Subliminal			
48a	Tusche, Anita, Stefan Bode, John-Dylan Haynes (2010). "Neural Responses to Unattended Products Predict Later Consumer Choices". The Journal of Neuroscience 30(23):8024–8031	x										insula and the medial prefrontal cortex		Visual, Semantic	Supraliminal Unconscious ve Supraliminal Conscious	Yes	U=C	B
48b		x	x	x		x		x	x									
49	Venkatraman, Vinod, John A. Clithero Gavan, J. Fitzsimons, vd (2012). "New scanner data for brand marketers: How neuroscience can help better understand differences in brand preferences". Journal of Consumer Psychology 22 : 143–153	x	x		x	x		x	x			anterior insula and the medial prefrontal cortex						
50	Kerbs, Claudia, Joanne Weinberg, Elizabeth Akesson (2018). Lippincott's Illustrated Review of Neuroscience. Philadelphia: Lippincott Williams & Wilkins	x	x		x		x	x	x			amygdala	reward circuitry: prefrontal cortex, nucleus accumbens, septal nuclei, hippocampus, amygdala, ventral tegmental area					A
51	Duan, Xujun, Qian Dai, Qiyong Gong, Huafu Chen (2010). Neural mechanism of unconscious perception of surprised facial expression". NeuroImage 52 : 401–407	x			x							unconscious emotion detection:<br>right amygdala<br>right thalamus	novelty detection:<br>parahippocampal gyrus<br>fusiform gyrus	Visual, Face Recognition, Emotion	Subliminal			A (for amygdala)
52	Jovasevic, Vladimir, Kevin A Corcoran, Katherine Leaderbrand, vd (2015). "GABAergic mechanisms regulated by miR-33 encode state-dependent fear". Nature Neuroscience 18(9) : 1265-1273	x			x		x	x		x		hippocampal areas						A
53	Derbaix, Christian, Jean-Luc Herrmann, Mathieu Kacha (2014). "Perception without awareness of supraliminal stimuli: A critical review and a proposition for an integrated model". Recherche et Applications en Marketing 29(2) : 57 –74	x	x				x	x										
54	Cahill, Larry, Richard Haier, James Fallon, vd (1996). "Amygdala activity at encoding correlated with long-term, free recall of emotional information". Proceedings of the National Academy of Sciences 93 : 8016-8021	x			x		x	x				amygdala		Visual, Emotion	Supraliminal Conscious			A
55	Whalen, Paul J.,Scott L. Rauch, Nancy L. Etcoff, vd (1998). "Masked Presentations of Emotional Facial Expressions Modulate Amygdala Activity without Explicit Knowledge" The Journal of Neuroscience 18(1):411–418	x			x							amygdala<br>sublenticular substantia innominata (si)		Visual, Face Recognition, Emotion	Subliminal			A

Supplementary Table 3: Comprehensive Review Study (continued)

	Reference	Unconscious	Brand Recognition	Economy	Emotion	Culture	Long-term Memory	Memory	Motivation and Award	Trauma	Metaphor	Unconscious ROI-Region of Interest	Other Brain Region	Motor<br>Visual<br>Lexical<br>Audio<br>Semantic<br>Physiologic<br>Face<br>Recognition<br>Touch<br>Emotion<br>Preference<br>Decision	Type of Stimuli:<br>1. Subliminal<br>2. Supraliminal Unconscious<br>3. Supraliminal Conscious<br>4. Both	If "Both":<br>Are the conscious and unconscious stimuli the same?	Relevant ROI Activation:<br>1. Unconscious only<br>2. U>C<br>3. U<C<br>4. U=C<br>5. Note for the compatibility<br><br>U: Unconscious Activation<br>C: Conscious Activation	Supported Method
56	Henke, Katharina, Valerie Treyer, Eva Turi Nagy, vd (2003). "Active hippocampus during nonconscious memories". Consciousness and Cognition 12 : 31–48	x						x				left and right hippocampus<br>right perirhinal cortex<br>hippocampal<br>parahippocampal		Visual, Face Recognition, Emotion	Subliminal and Supraliminal Conscious	Yes		A, D
57	Henke, Katharina (2010). "A model for memory systems based on processing modes rather than consciousness." Nature Reviews Neuroscience, 11(7): 523-532.	x					x	x										
58	Ruch, Simon, Elizabeth Herbert, Katharina Henke (2017). "Subliminally and Supraliminally Acquired Long-Term Memories Jointly Bias Delayed Decisions". Frontiers in Psychology 8 (1542)	x					x	x						Visual, Face Recognition, Semantic	Subliminal and Supraliminal Conscious	Yes		A
59	Glascher, Jan, Ralph Adolphs (2003). "Processing of the Arousal of Subliminal and Supraliminal Emotional Stimuli by the Human Amygdala". The Journal of Neuroscience 23(32):10274–10282	x			x							amygdala		Visual, Emotion	Subliminal and Supraliminal Conscious	Yes	U>C	A, B
60	LaBar, Kevin S., Roberto Cabeza (2006). "Cognitive neuroscience of emotional memory". Nature Reviews Neuro 7: 54-64	x			x		x	x				amygdala<br>hippocampus<br>entorhinal cortex<br>prefrontal cortex						A
61	Costafreda, Sergi G., Michael J. Brammer, Anthony S. David, Cynthia H.Y. Fu (2008). "Predictors of amygdala activation during the processing of emotional stimuli: A meta-analysis of 385 PET and fMRI studies". Brain Research Reviews, 58: 57-70	x			x							amygdala						A
62	Murphy, Fionnuala C., Ian Nimmo-Smith, Andrew D. Lawrence (2003). "Functional neuroanatomy of emotions: A meta-analysis". Cognitive, Affective, & Behavioral Neuroscience, 3 (3): 207-233				x							amygdala,<br>insula<br>globus pallidus<br>lateral orbitofrontal cortex						A
63	Phillips, Mary L., Leanne M. Williams, Maike Heining, vd, (2004). "Differential neural responses to overt and covert presentations of facial expressions of fear and disgust". NeuroImage 21: 1484– 1496.	x										fusiform						
64	McClure, Samuel M., Jian Li, Damon Tomlin, vd (2004). "Neural Correlates of Behavioral Preference for Culturally Familiar Drinks". Neuron, 44: 379–387		x		x	x							hippocampus and dorsolateral prefrontal cortex					
65	Brown, R.A., R. Seligman (2009). "Anthropology and cultural neuroscience: creating productive intersections in parallel fields". Şu kitapta: Ed. J.Y. Chiao. Cultural Neuroscience: Cultural Influences on brain function. Progress in brain research 178. Great Britain: Elsevier: 31-42.				x	x							amygdala					
66	Dominguez, Juan F. D., E. Douglas Lewis, Robert Turner, Gary F.Egan (2009). "The brain in culture and culture in the brain: a review of core issues in neuroanthropology". Şu kitapta: Ed. J.Y. Chiao. Cultural Neuroscience: Cultural Influences on brain function. Progress in brain research 178. Great Britain: Elsevier: 43-64.					x							prefrontal cortex					
67	Schacter, Daniel L., Angela H. Gutchess, Elizabeth A. Kensinger (2009). "Specificity of memory: Implications for individual and collective remembering" Şu kitapta: Ed. P. Boyer, J. Wertsch. Memory in Mind and Culture. Cambridge: Cambridge University Press: 83-111.				x	x							amygdala, hippocampus, orbitofrontalcortex					
68	Bernard Roullet, Olivier Droulers /2010). Neuromarketing, Le marketing revisité par les neurosciences du consommateur. Paris: Dunod		x										reduced activation in the dorsolateral prefrontal, posterior parietal, and occipital cortices and the left premotor area.<br>simultaneously, increased activity in the inferior precuneus and posterior cingulate, right superior frontal gyrus, right supramarginal gyrus, and, most pronounced, in the ventromedial prefrontal cortex.					
69	Schaefer, Michael (2009). "Neuroeconomics: in search of the neural representation of brands". Şu kitapta: Ed. J.Y. Chiao. Cultural Neuroscience: Cultural Influences on brain function. Progress in brain research 178. Great Britain: Elsevier: 238-252.		x										striatum and dorsolateral part of prefrontal cortex					
70a	Schaefer, Michael, Michael Rotte (2020). "Combining a semantic differential with fMRI to investigate brands as cultural symbols". Scan, 5: 274-281		x										medial prefrontal cortex					
70b			x										dorsolateral part of the prefrontal cortex (dlpfc), midbrain and hippocampi, ventromedial prefrontal cortex (vmpfc)					
70c			x										anterior medial prefrontal cortex (ampfc)					
71	Erk, Susanne, Manfred Spitzer, Arthur P.Wunderlich, vd (2002). "Cultural objects modulate reward circuitry". Neuroreport, 13(18): 2499-2503.		x										right ventral striatum, left orbitofrontal cortex, left anterior cingulate and bilateral prefrontal cortex, right fusiform gyrus and left lateral occipital complex					

Supplementary Table 3: Comprehensive Review Study (continued)

	Reference	Unconscious	Brand Recognition	Economy	Emotion	Culture	Long-term Memory	Memory	Motivation and Award	Trauma	Metaphor	Unconscious ROI-Region of Interest	Other Brain Region	Motor<br>Visual<br>Lexical<br>Audio<br>Semantic<br>Physiologic<br>Face<br>Recognition<br>Touch<br>Emotion<br>Preference<br>Decision	Type of Stimuli:<br>1. Subliminal<br>2. Supraliminal Unconscious<br>3. Supraliminal Conscious<br>4. Both	If "Both":<br>Are the conscious and unconscious stimuli the same?	Relevant ROI Activation:<br>1. Unconscious only<br>2. U>C<br>3. U<C<br>4. U=C<br>5. Note for the compatibility<br><br>U: Unconscious Activation<br>C: Conscious Activation	Supported Method
72a	Schaefer Michael, Michael Rotte (2007). "Thinking on luxury or pragmatic brand products: Brain responses to different categories of culturally based brands". Brain Research 1165: 98-104		x										bilateral<br>superior frontal gyri, hippocampus and posterior cingulate					
72b			x										medial prefrontal cortex (mpfc) and precuneus					
72c			x										left superior frontal gyrus and anterior cingulate cortex acc).					
73	Schaefer, Michael, Harald Berens, Hans-Jochen Heinze,Michael Rotte (2006). "Neural correlates of culturally familiar brands of car manufacturers". NeuroImage 31: 861 – 865		x										medial prefrontal cortex					
74	Schaefer, Michael, Michael Rotte (2006). "Favorite brands as cultural objects modulate reward circuit". NeuroReport 18(2): 141-145		x										ventral striatum, dorsolateral prefrontal cortex, and inferior frontal gyrus					
75a	Santos, José Paulo, Luiz Moutinho, Daniela Seixas, Sofia Brandão (2012). "Neural correlates of the emotional and symbolic content of brands: A neuroimaging study". Journal of Customer Behaviour, 11(1): 69-93		x										right and left frontal orbital cortex					
75b			x										insular cortex and frontal orbital cortex					
75c			x										frontal medial cortex					
75d			x										an activation in the ventro medial prefrontal cortex (which comprises the frontal medial cortex, the ventral medial frontal pole, and the subcallosal cortex) -- emotion based area					
75e			x										deactivation in the dorso lateral prefrontal cortex (a brain region that includes the dorso lateral frontal pole and the middle frontal gyrus) - working memory based					
75f			x										amygdala					
75g			x										paracingulate gyrus,<br>temporo-parietal junction (which encompasses the angular gyrus and the supramarginal gyrus)					
75h			x										armfc - anterior<br>rostral medial frontal cortex					
75i			x										ventral paracingulate gyrus<br>and the ventral medial frontal pole					

Supplementary Material F: Preparation of stimuli (visuals)

The creation of images or visuals as stimuli and the preparation of the design experiment to be displayed during the fMRI task are critical processes in the research to determine the unconscious effect of the migration theme on participants' brains. At this stage, the selection of truck and background images (best representing the migration theme and the control variables), their transformation into visuals with the help of a professional visual arts expert, and the completion of the experimental design and its simulations have been achieved.

First, a total of 248 different anonymous truck images were collected from the internet and expert archives and categorized. Processes were implemented to minimize visual differences (color, tone, size, angle) and brand effects (hiding brand logos and characteristic design details). In this study, the main purpose was to distinguish the effect of background themes, and brand predictability was especially attempted to be eliminated. Because the brand phenomenon is a complex set of psychological, sociological, and cultural meanings, it can elicit distinct activations in the brain (Erk et al., 2002; McClure et al., 2004; Plassmann, Ramsøy, and Milosavljevic, 2012; Schaefer and Rotte, 2010). Even if the logo and design details are hidden, truck drivers who have worked in this sector for many years can still guess the truck brands and models. To identify the least predictable truck brands, 17 trucks, visually filtered, were shown to a team of six experts, who were asked to identify the truck brands. As a result of this study, six brands were identified as the least predicted. To increase the number of images, two truck images from completely different markets were added to the work (see Supplementary Material G). For secondary validation, the study was repeated with 34 participants who had also participated in the fMRI study, yielding similar results.

To use the migration theme in the fMRI task, it needs to be visualized and made into a background image. To answer a research question about which image best represents the theme of migration, first, anonymous images with the theme of migration were collected from the Internet, and the number was reduced to four. These images were then filtered by a visual arts expert. In the study involving 25 truck drivers, both qualitative and quantitative data were collected and analyzed, and the most suitable image was selected (see Supplementary Material H). For secondary validation, the study was repeated with 34 individuals who had participated in fMRI studies, yielding results similar to those obtained previously.

To determine the themes of the control variables (Y and Z), instead of making a choice based on experience, the marketing communication studies of the two brands, which are the leaders of the Turkish truck market over the years were used (Ford Otosan 2015a, Ford Otosan 2015b). In this study, 38 past communication studies for the first heavy truck brand and 44 for the second heavy truck brand were scanned. Then, using clustering logic, all these themes were grouped into 8 parent themes: comfort, strength, economy, outstanding/good, performance, new product, capacity, and others. As a result of the analysis made using qualitative research methods, the theme of economy was chosen and the theme was illustrated by a visual arts expert (see Supplementary Material I). As the last step, the Neutral background (Z) was chosen as the second control variable of the study (see Supplementary Material I). As a result, eight trucks were placed on three background themes, and 24 images were obtained to be used in the fMRI experiment (see Supplementary Material I).

To design the fMRI experiment, the following should be determined: type of design, design paradigm and timing, timing and experiment parameters (Trial Duration TD, Stimulus Duration SD, Inter Trial Interval ITI, Inter Stimulus Interval ISI, Stimulus Onset Asynchrony OA, stimulus quantity), number

of participants (Amaro and Barker, 2006; Lindquist, 2008). According to the model of this research, it is desired to analyze the activations of three different stimuli (economy, neutral, and migration) in the brain and whether each of them creates activation with a significant difference compared to the other. For this reason, considering the design types and their sub-types (Amaro and Barker, 2006), the categorical design and its subtraction type were chosen. Two key aspects of the quality of the experimental design, namely detection power (a measure of the ability to detect an activation) and estimation efficiency (a measure of the ability to predict the shape of the hemodynamic response), need to be optimized (Amaro and Barker, 2006; Lindquist, 2008; Liu, 2012). For this, the design paradigm and timing are very critical. It is essential to choose between block design, event-related design, or mixed design as the design paradigm. If event-related design is selected, it is crucial to choose one of its subtypes: slow event-related design or rapid event-related design. Rapid event-related is also of two types, fixed ISI and random ISI. The event-related design maximizes estimation efficiency, while the block design maximizes detection power (Amaro and Barker, 2006; Lindquist, 2008; Liu, 2012). In this study, it is important to determine which parts of the brain are activated by unconscious stimuli. Therefore, it would be beneficial to choose a block design that provides high detection power and to maximize the number of participants and the number of stimuli. However, both the pilot study results and the literature review indicated that the block design was not suitable for this research. Problems were encountered because the participants were mentally adjusting to a block; they were able to predict the effect of every stimulus, even those not observed, which was not manageable for many stimulus types. Additionally, the number of participants and stimuli had to be limited due to the research's challenges. Therefore, it was decided to use the event-related design sequence. However, the starting point —the need for high detection power in this research —remains a valid requirement. Despite the use of event-related design, thereafter, ways to maximize the detection power were investigated and this was achieved in two ways: Choosing the rapid event-related design, using its jittered and randomized ISI (or ITI) method and using the Genetic Algorithm Method that enables to find the ISI (or ITI) and the most appropriate stimulus sequence which maximizes detection power and estimation efficiency (Durnez, Blair, and Poldrack, 2017; Kao and Mittelmann, 2014, Wager and Nichols, 2002). After evaluating the preliminary and final simulations conducted in a Python-based program package and a web-based software (NeuroPowerTools, 2020), it was decided to proceed with ITI min = 2 and ITI max = 4 options (Çimen, 2021). The raw data obtained from the simulation were converted into duration and time information. After this preprocessing, the duration of each of the three stimuli, in seconds, was determined, along with the start and end times, to decimal precision. Thanks to these measures and decisions, it has been possible to reach a satisfactory detection power. As a result, the images of Economy, Neutral, and Migration, which served as the stimuli, were used as the backgrounds. Truck images were arranged in a fixed order within each truck, and each truck was repeated 9 times. The Economy with the Background 1 stimulus was repeated 22 times, the Neutral with the Background 2 stimulus was repeated 17 times, and the migration with the Background 3 stimulus was repeated 33 times. According to these data, the final sequence was made in MS PowerPoint format. Subsequently, the sequence and duration of the images were uploaded to the fMRI software by the software specialist. The program, which works in harmony with the fMRI device, showed these images to the participants in the desired order, with the desired ITIs, and at the desired timing during the task (for more details, see Supplementary Material J).

Supplementary Material G: Selection of Truck Pictures and Preparation of Images

A total of 248 different truck pictures (Supplementary Figure 3) were accessed on the internet (anonymous) and from the archives of experts.

Supplementary Figure 3: Examples of Anonymous Truck Pictures Detected by Internet Search

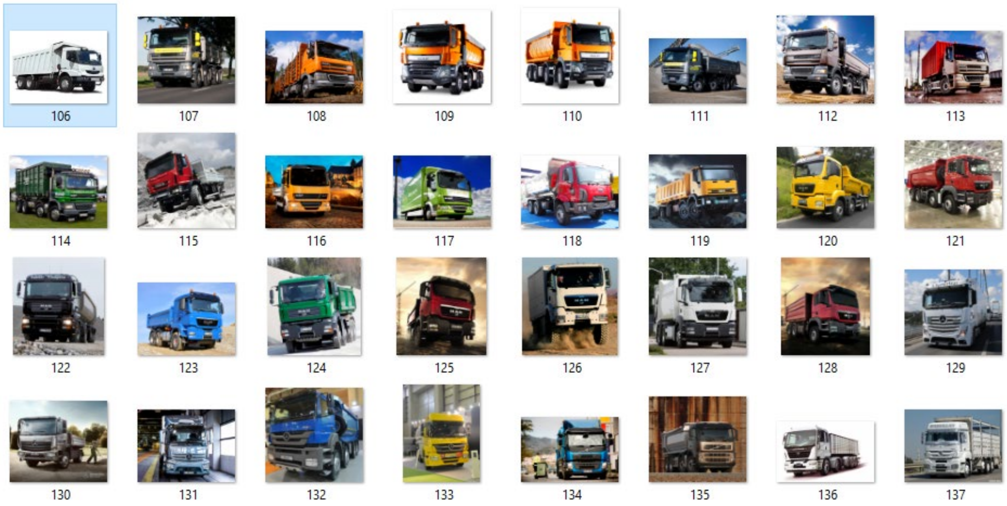

The truck images found differed in many ways. Selecting the most appropriate ones before conducting the visual study would improve the quality of subsequent studies. For these reasons, truck pictures were categorized according to the following criteria. These criteria include truck type (road truck, tractor unit (TIR), construction truck), view angle (right, left, front, or side), image quality (number of pixels), and guessing the brand.

The research aims to distinguish the effect of the background visual representing the theme discovered from that of control-variable backgrounds on the unconscious. For this reason, all participants will see the same truck pictures, which will not differ significantly. For example, visual differences should be avoided as much as possible, as one truck is red, the other is white, one is from the side, the other is from the front, one is of high visual quality, and the other is low, as this will create different activations in the brain. Another important factor that may create a noise factor is how easily the truck brand can be guessed. Because the brand phenomenon is a very complex set of psychological, sociological, and cultural meanings (Erk, et al., 2002; McClure, et al., 2004; Schaefer, et al., 2006; Schaefer and Rotte, 2006; Schaefer and Rotte, 2007; Roullet and Droulers, 2010; Plassmann, Ramsøy, and Milosavljevic, 2012) is likely to create different activations in the brain. This may create undesirable effects in the study, where the main purpose is to distinguish the effect of the background themes. For this reason, it is aimed to eliminate these effects as much as possible. Differentiating effects were identified, and the following studies were carried out on truck paintings by a visual arts expert:

- Painting trucks of different colors in the same tone of white
- Bringing trucks of different sizes to the same size
- Bringing truck images from different angles to the same angle
- Hiding brand logo and design details
- Placement in standard background template

Identifying the Trucks with the Least Guessed Brands

Although the brand logo and design details are visually hidden, it is possible to guess the truck brands based on the general design features, as with every product. Even if the logo and design details are hidden, truck drivers who have worked in this sector for many years can still guess the truck brands and models. An additional study was carried out to minimize the effect of truck drivers put it, "I can tell what brand a truck is even when it is coming from a distance on the road." In this study, 17 visually filtered (simulated) trucks were shown to a team of 6 automotive (truck business unit) experts, and they were asked to identify the truck brands. Two of these experts work as design studio experts, two as marketing experts, one as a marketing strategy expert, and one as a vehicle engineering expert.

According to the study results shared in Supplementary Table 4, while 6 experts tried to guess 17 truck brands, some of them could not say what the brands were, some guessed the brand but could not know the brand, some said a brand but could not know it, although they were not sure, and some said a brand and knew it, although they were not sure. According to this study, 6 brands from the Supplementary Table 4 were identified as the least guessed brands. The yellow-marked cells show the experts' inexact guesses in the survey. According to an assessment, the research team selected green-marked visuals as less ambiguous truck brand visuals and decided to use them in the research.

Supplementary Table 4: Brand Guess Study Results

	The Brand in Reality	Design Studio Expert 1	Marketing Expert 1	Marketing Expert 2	Strategy Expert	Design Studio Expert 2	Vehicle Engineer
Visual 1	Iveco	Iveco	Iveco	Iveco	May be Scania	Iveco	Iveco
Visual 2	Man	Man	Man	Man	Man	Man	Man
Visual 3	DAF	DAF	No Guess	No Guess	DAF	No Guess	May be DAF
Visual 4	Scania	Scania	Scania	Scania	Mercedes	No Guess	Scania
Visual 5	BMC	BMC	BMC	BMC	No Guess	BMC	BMC
Visual 6	Iveco	Iveco	Iveco	May be MAZ or Man	May be DAF	May be Iveco	Iveco
Visual 7	Iveco	May be Iveco	BMC	May be from China	May be KAMAZ	No Guess	No Guess
Visual 8	Man	Man	Man	Man	Man	May be Man	May be MAN
Visual 9	Scania	Scania	Scania	Scania	Volvo	Scania	Scania
Visual 10	Scania	Scania	Scania	Scania	Scania	Scania	Scania
Visual 11	Volvo	Volvo	Volvo	Volvo	Volvo	Volvo	Volvo
Visual 12	Volvo	Volvo	Volvo	Volvo	No Guess	Volvo	Volvo
Visual 13	Volvo	Volvo	Volvo	Volvo	No Guess	Volvo	May be Volvo
Visual 14	DAF	DAF	DAF	May be Scania	DAF	No Guess	DAF
Visual 15	Cargo	Cargo	Cargo	Cargo	Cargo	Cargo	Cargo
Visual 16	Cargo	Cargo	Cargo	Cargo	Cargo	Cargo	Cargo
Visual 17	Iveco	Iveco	Iveco	Kamaz or Maz	Scania	May be Iveco	May be Iveco

During the experimental design, it was decided that increasing the number of trucks would be beneficial, so two additional truck images were added. The new truck images were searched from completely different markets. Two trucks from the Far East truck market were added to the study. The final version of the trucks selected and visually filtered at the end of these studies is shown in Supplementary Figure 4.

Supplementary Figure 4: Truck Visuals After Filtering and Remediation

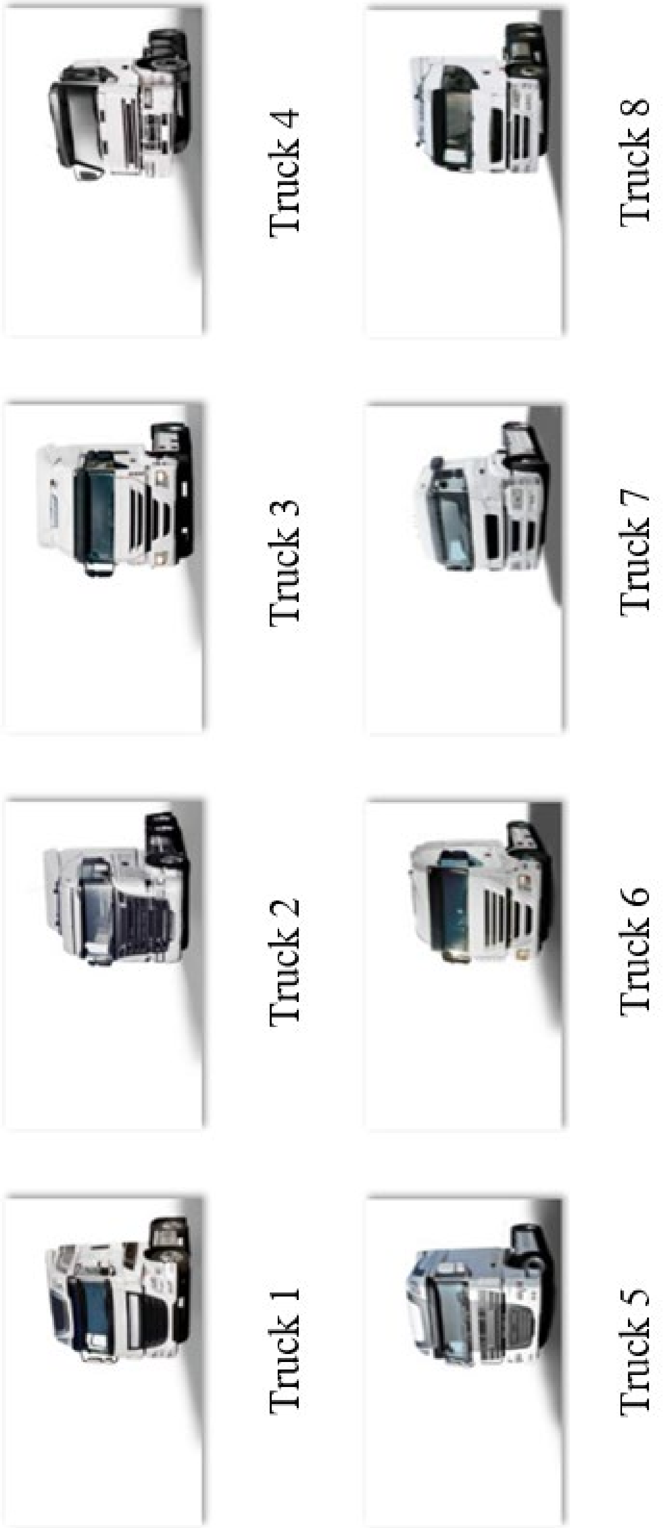

Supplementary Material H: Selection of Experiment Background Theme and Visual

Preparation

The experimental background theme was determined through psychological analysis in the first stage of the research, and the theme of migration was chosen as the cultural code for the truck product. For the migration theme to be used in fMRI testing, it must be visualized and turned into a background image. At this point, a research question arises: which image best represents the theme of migration? To answer this research question, various migration-themed images were collected on the open-source internet. Their number was reduced to four and shared with the visual arts expert. At the end of the visual study, 4 images were obtained. These visuals were prepared in MS PowerPoint presentation format for display to the participants, as shown in Supplementary Figure 5. Face-to-face interviews were conducted with 25 truck drivers in Northwest Anatolia who did not participate in the fMRI study. The data were collected in a standard form (Supplementary Figure 6).

Supplementary Figure 5: Survey Presentation for Migration Visuals Selection

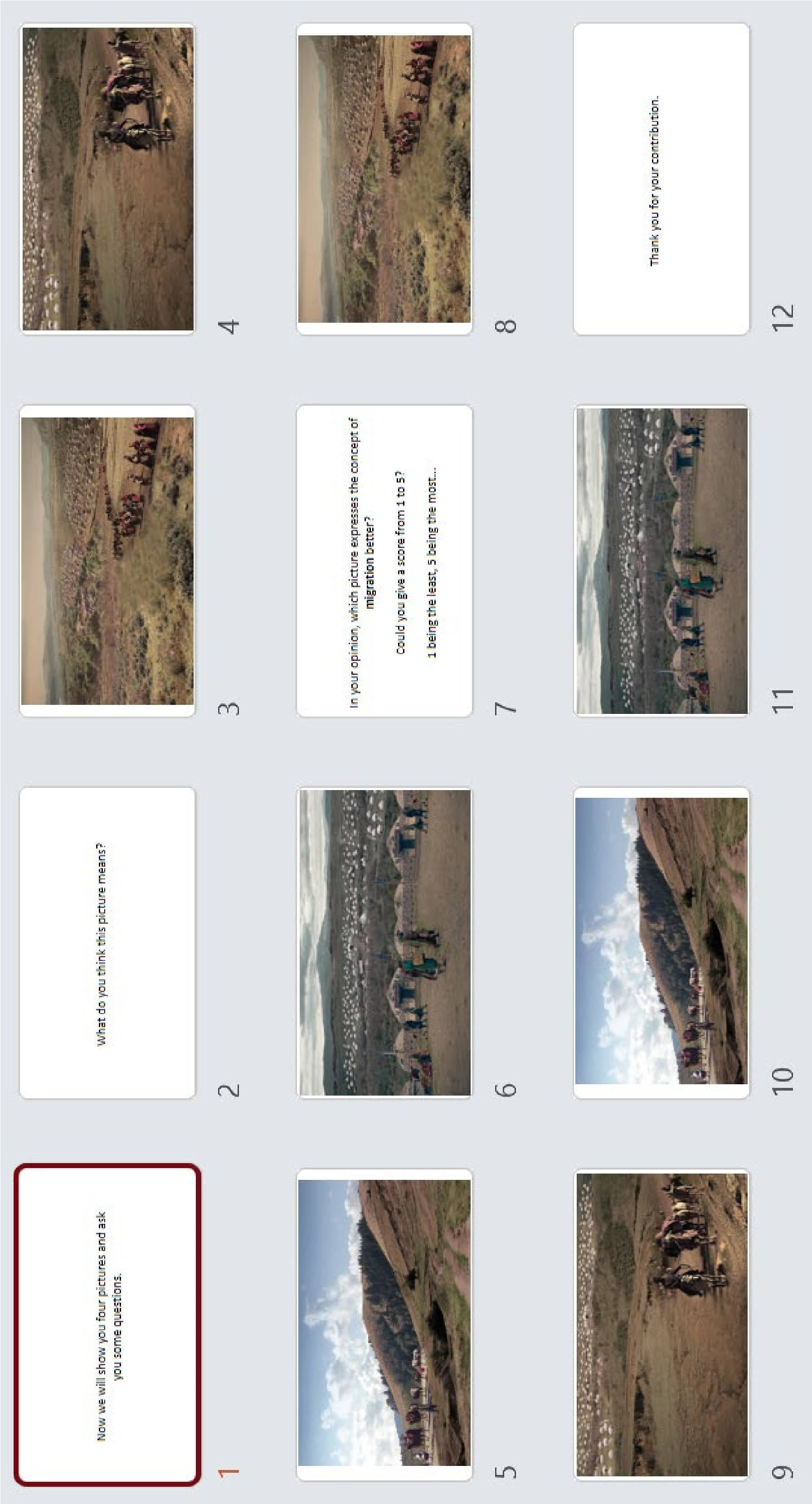

Supplementary Figure 6: Data Collection Form for Background Picture

DATA COLLECTION FORM FOR BACKGROUND PICTURE

Participant Name:

What do you think this picture means?

Picture 1:

Picture 2:

Picture 3:

Picture 4:

In your opinion, which picture expresses the concept of migration better? (1 the least, 5 the most)

Picture 1:

Picture 2:

Picture 3:

Picture 4:

The collected data were analyzed using three different methods and then interpreted together. In the first instance, quantitative scores ranging from 0 to 5 were collected from the participants. In the second one, the number of times participants repeated keywords (migration, nomad, immigrant, moving, caravan, etc.) was counted from their qualitative expressions. In the third one, whether they expressed the keywords or not was counted, regardless of the number of repetitions. The research results are summarized in Supplementary Table 5.

Supplementary Table 5: Survey Results of Background Picture

**Selection of Background Visual with Group 4 Data**	**Picture 1**	**Picture 2**	**Picture 3**	**Picture 4**
Quantitative (Based on scores)	94	72	108	49
Qualitative (Based on word repetition)	21	17	21	1
Qualitative (Based on "exists /doesn't exist")	18	14	21	1

Note: The highest numbers are yellow-marked

As a result of the study, it was decided to use Picture 3 (Supplementary Figure 7) as the experimental theme in fMRI studies, that is, as a visual representation of the migration background.

Supplementary Figure 7: The Selected Visual for the Background Representing the Migration (Picture 3)

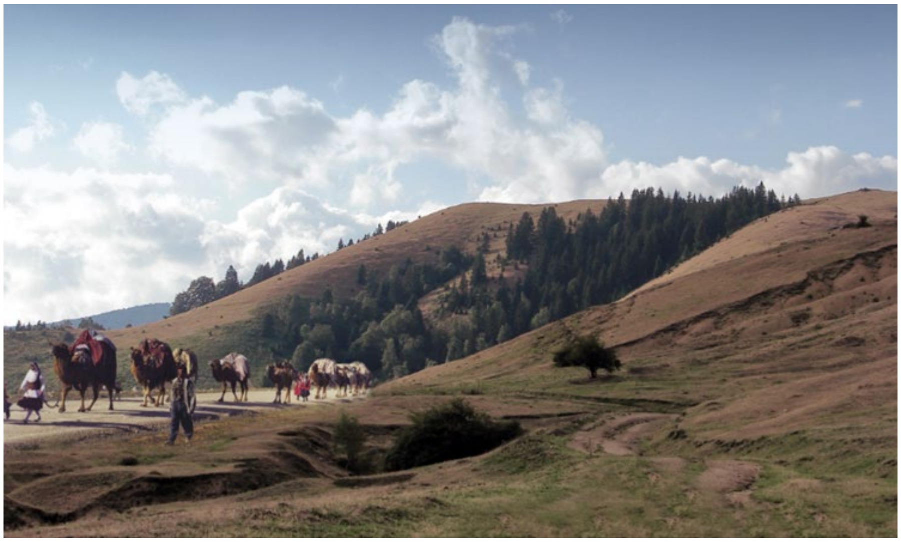

Supplementary Material I: Selection of Control Variables and Preparation of Background Images

Supplementary Table 6: Results of the Screening Research Conducted for the Selection of Control Variables (Themes) Based on the Ads of Brand A

Ads	Themes of Brand A	Comfort	Power	Economy	Superior/Good	Performance	New Product	Capacity	Other	Repetition
1	Innovation						1			1
2	Fuel			1						1
3	Innovation						1			1
4	Comfort	1								1
5	Innovation						1			1
6	Strong		1							1
7	High capacity							1		1
8	Strong		1							1
9	Strong		1							1
10	Safety								1	1
11	Favorable economy			1						1
12	Difficult and heavy load		1							1
13	Comfort	1								1
14	Carries load		1							1
15	New product						1			1
16	Capacity							1		1
17	New product						1			1
18	Hard conditions		1							1
19	Comfort	1								1
20	Origin								1	1
21	Origin								1	1
22	Hard conditions		1							1
23	Comfort	1								1
24	Power and economy		1	1						2
25	Strength		1							1
26	New product						1			1
27	Miscellaneous								1	1
28	Capacity, superior				1			1		2
29	New product						1			1
30	Strength		1							1
31	Strength		1							1
32	Capacity, less spending			1				1		2
33	Comfort	1								1
34	New product						1			1
35	Miscellaneous								1	1
36	Long life, strong		1	1						2
37	Strong		1							1
38	Resistant		1							1
	**Total**	5	14	5	1	0	8	4	5	42
	**Average x 100**	11,90	33,33	11,90	2,38	0,00	19,05	9,52	11,90	

Supplementary Table 7: Results of the Screening Research Conducted for the Selection of Control Variables (Themes) Based on the Ads of Brand B, and Overall Results

	Themes of Brand B	Comfort	Power	Economy	Superior/Good	Performance	New Product	Capacity	Other	Repetition
1	Fuel			1						1
2	Like a car	1								1
3	Resistant		1							1
4	Economy			1						1
5	Strong		1							1
6	Comfort	1								1
7	Comfort	1								1
8	Strong		1							1
9	Strong		1							1
10	Comfortable	1								1
11	Leader				1					1
12	Truck of the year				1					1
13	Brave fellow		1							1
14	Carries stones		1							1
15	Leader				1					1
16	Every load, economical		1	1						2
17	Fuel			1						1
18	Power and economy		1	1						2
19	Fuel			1						1
20	Economy and performance			1		1				2
21	Origin								1	1
22	Fuel			1						1
23	Capacity, profit			1				1		2
24	Leader				1					1
25	New Product						1			1
26	Strong		1							1
27	Productive			1						1
28	Leader				1					1
29	Leader				1					1
30	Comfort, fuel	1		1						2
31	Spare part			1						1
32	Trustworthy								1	1
33	Trustworthy								1	1
34	Chic				1					1
35	Fuel			1						1
36	Leader				1					1
37	New product						1			1
38	Trustworthy								1	1
39	Profitable			1						1
40	Fuel			1						1
41	Environmentalist								1	1
42	Beautiful				1					1
43	Environmentalist								1	1
44	Leader				1					1
	**Total**	5	9	15	10	1	2	1	6	49
	**Average x 100**	10,20	18,37	30,61	20,41	2,04	4,08	2,04	12,24	
	**Average of averages x 100**	11,05	25,85	21,26	11,39	1,02	11,56	5,78	12,07	

These control variables were evaluated considering both their dominance and ability to be expressed visually, and the theme of economy was chosen. As the third control variable, a neutral background was chosen to create maximum contrast. Colors were chosen the same to minimize visual distortions. The images obtained from these studies are shown in Supplementary Figure 8.

Supplementary Figure 8: Background Visuals with a Sample Truck: Migration, Economy, and Neutral

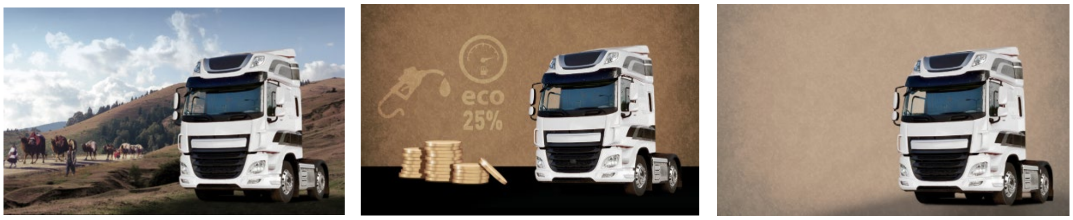

As a result of the studies, 24 visuals to be used in the fMRI experiment were obtained by placing 8 trucks on 3 background themes (economy, migration, neutral). All of these are shared in Supplementary Figure 9.

Supplementary Figure 9: 24 Final Visuals Consisted of 8 Trucks and 3 Backgrounds

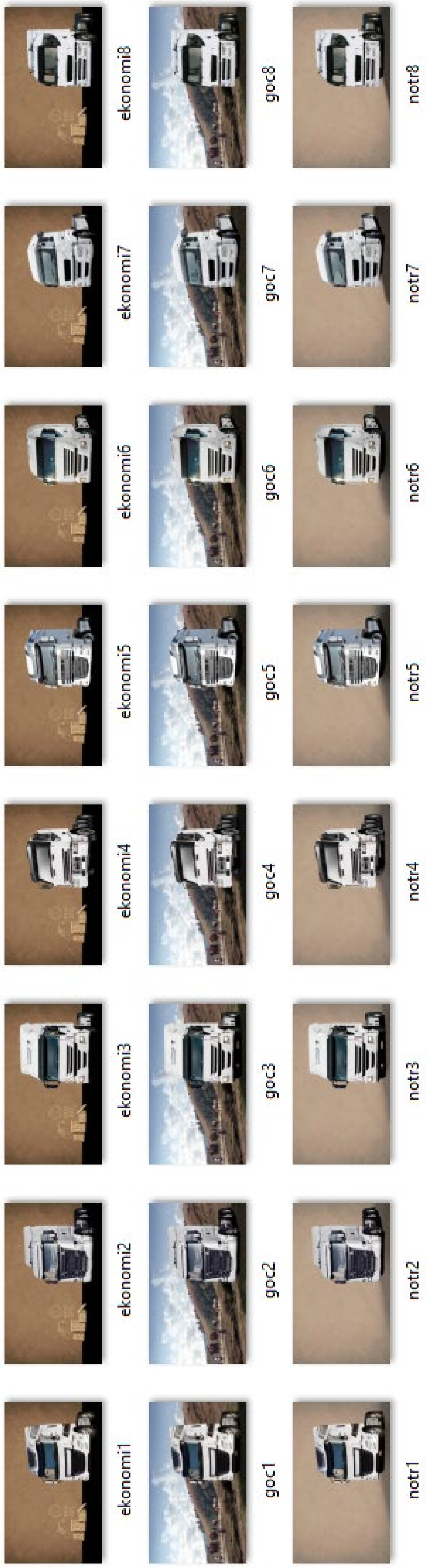

Supplementary Material J: Design Experiment Parameters by Genetic Algorithm

19 preliminary simulations were conducted online to determine the critical inputs for the research. The purpose of these preliminary simulations is to determine the impact of critical inputs, especially on detection power. For this purpose, simulations were made by differentiating the following inputs: Number of Stimulus Type, Stimulus Duration (sec), Trial Count, ITI min, ITI max, and Avg. In particular, ITI min and ITI max values have a significant impact on both detection power and the duration of the fMRI task. For this reason, special focus was placed on this input. A significant difficulty was encountered at this stage. It also includes the following genetic algorithm inputs regarding the number of generations, namely: Number of generations (iterations or cycles), number of generations in the pre-run to define the maximum efficiency and detection power, and number of stable generations to reach convergence. The list of inputs for each scenario is summarized in a format such as in Supplementary Figure 10.

Supplementary Figure 10: Review Screen of Genetic Algorithm Inputs (http://www.neuropowertools.org/design/review/)

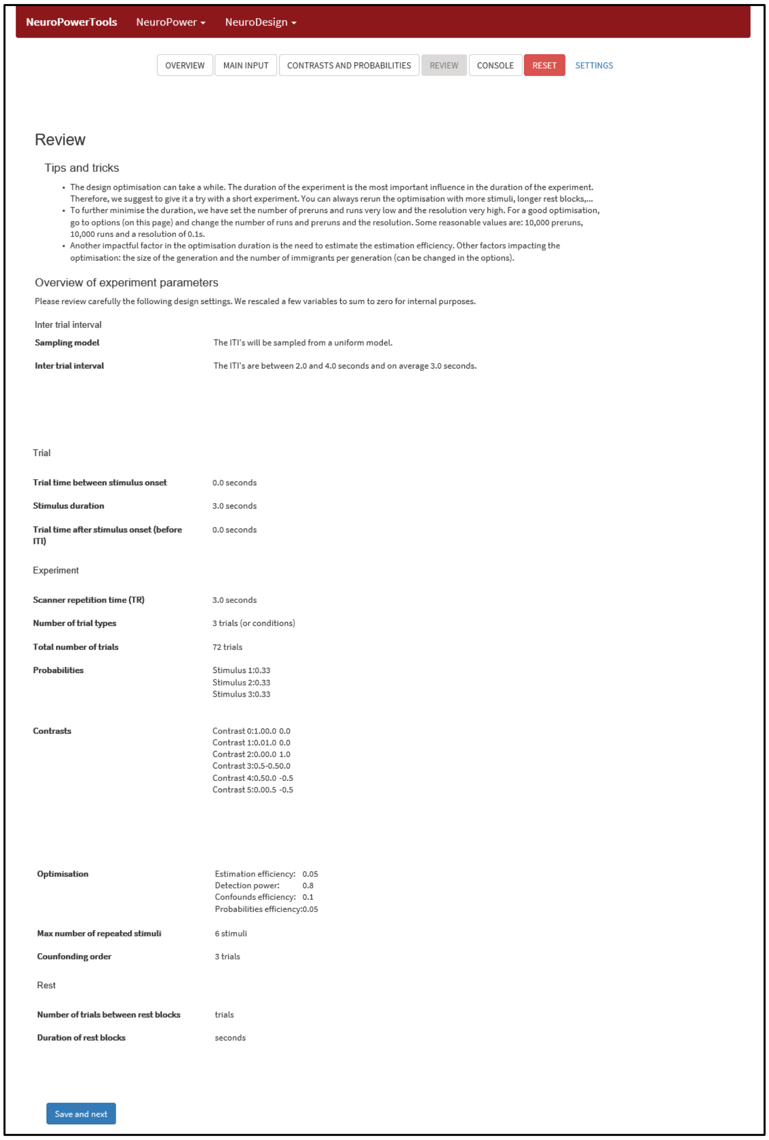

When each simulation is completed, the program shares the reports specified in Supplementary Figure 11, which show “Design Efficiency, Detection Power, Trial probabilities, Psychological confounds, and Overall Fit”; Supplementary Figure 12; Supplementary Figure 13; and Supplementary Table 8. Although it is possible to examine each stimulus separately, it can also be evaluated collectively. In this way, to determine the ITI min and max values, which are the most critical among the input parameters, a total of 19 simulations were conducted in the web-based simulation program, with relatively low generation numbers. Their input and output values are recorded in Supplementary Table 8.

Supplementary Figure 11: Program Graphic Results (http://www.neuropowertools.org//design/runGA/)

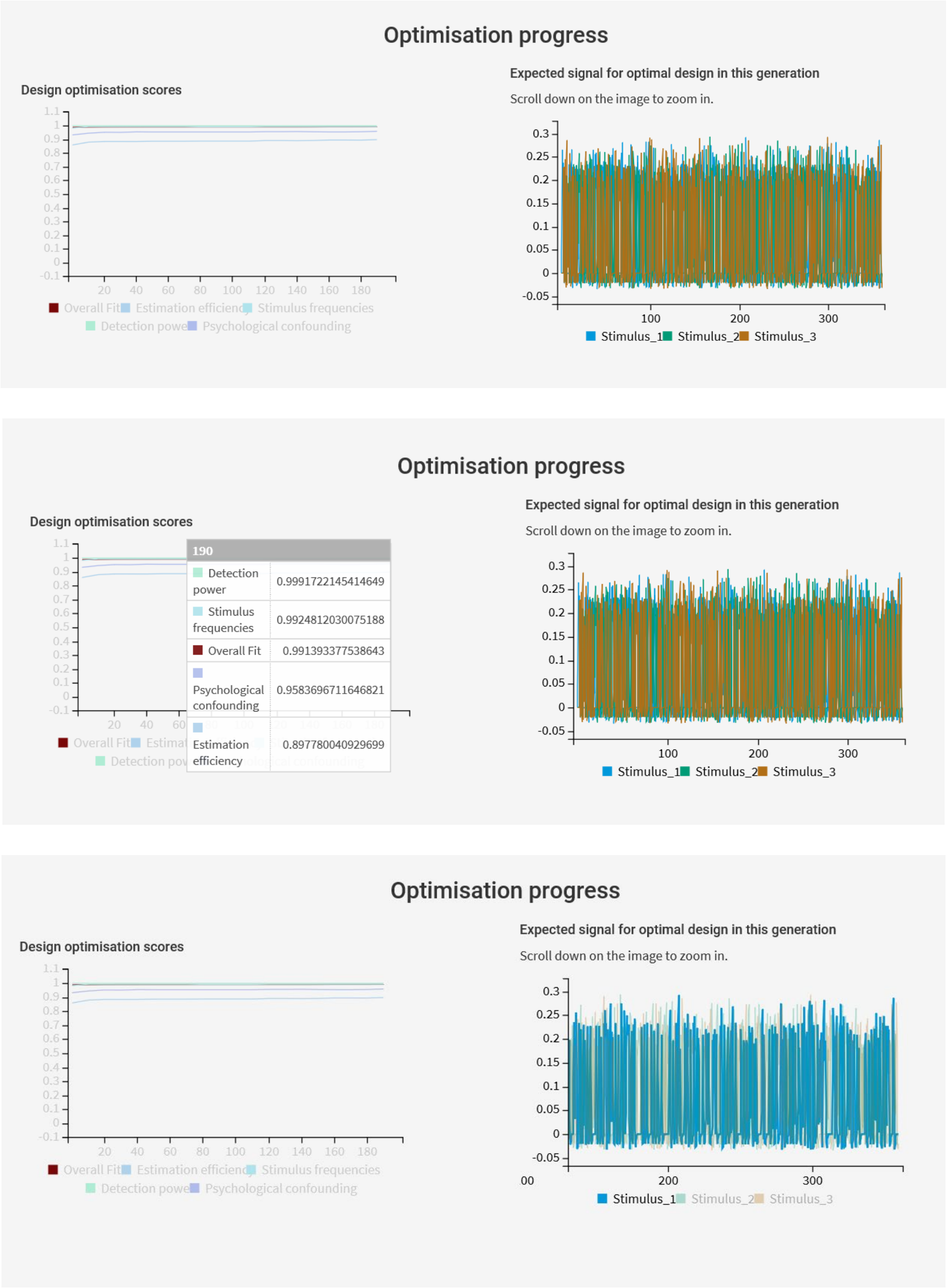

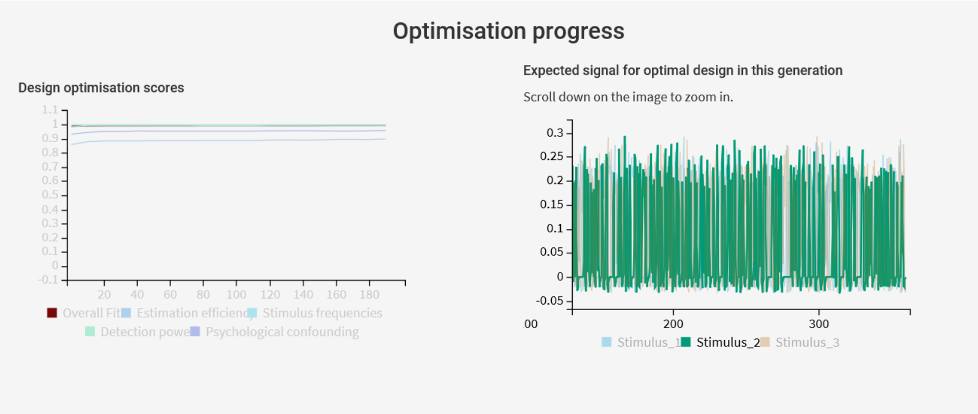
Optimisation progress
Design optimisation scores
Expected signal for optimal design in this generation
Scroll down on the image to zoom in.
Overall Fit
Estimation efficienc
Stimulus frequencies
Detection powe
Psychological confounding
Stimulus_1
Stimulus_2
Stimulus_3

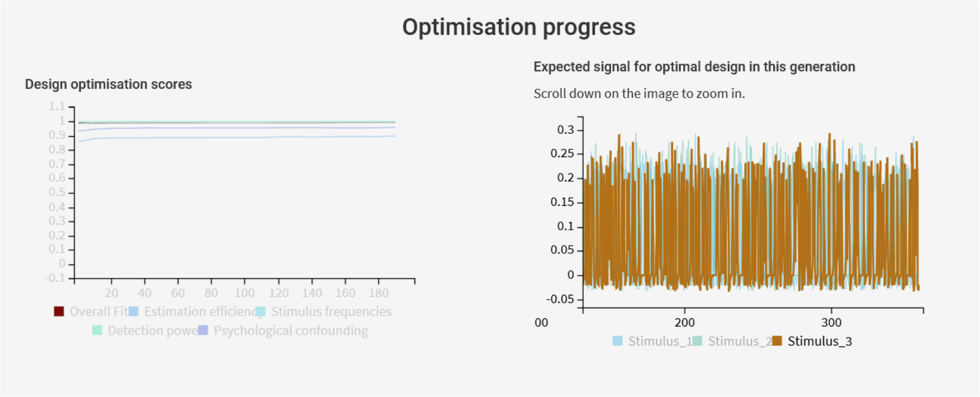
Optimisation progress
Design optimisation scores
Expected signal for optimal design in this generation
Scroll down on the image to zoom in.
Overall Fit
Estimation efficienc
Stimulus frequencies
Detection powe
Psychological confounding
Stimulus_1
Stimulus_2
Stimulus_3

Supplementary Table 8: Design Experiment Simulations Results and Comparison of the Different Simulations

		Sim 1	Sim 2	Sim 3	Sim 4	Sim 5	Sim 6	Sim 7	Sim 8	Sim 9	Sim 10	Sim 11	Sim 12	Sim 13	Sim 14	Sim 15	Sim 16	Sim 17	Sim 18	Sim 19
**Number of Stimulus Type**		3	3	3	3	NA	3	3	3	3	3	3	3	3	4	3	3	3	3	3
**Scanner TR (sec)**		3	3	3	3	3	3	3	3	3	3	3	3	3	3	3	3	3	3	3
**Seconds before stimulus**		0	0	0	0	0	0	0	0	1	0	0	0	0	0	0	0	0	0	0
**Stimulus Duration (sec)**		3	3	3	3	NA	3	3	3	1	3	3	1	5	3	3	3	12	3	3
**Seconds after stimulus**		0	0	0	0	0	0	0	0	1	0	0	0	0	0	0	0	0	0	0
**Duration /Trial Count**		360	432	72	504	540	540	612	684	72	72	72	72	72	72	200	72	72	72	72
**ITI Model**		Uniform	Uniform	Uniform	Uniform	Uniform	Uniform	Uniform	Uniform	Uniform	Uniform	Uniform	Uniform	Uniform	Uniform	Uniform	Uniform	Uniform	Uniform	Uniform
**ITI min**		1	1	1	1	1	1	1	1	1	1	10	1	1	1	1	2	5	2	2
**ITI max**		3	5	5	7		8	10	12	2	2	15	3	3	3	3	5	7	4	4
**Avg**		2	3	3	4	4,5	4,5	5,5	6,5	1,5	1,5	12,5	2	2	2	2	3,5	6	3	3
**Contrast: Check to include all pairwise contrast**		Yes	Yes	Yes	Yes	Yes	Yes	Yes	Yes	Yes	Yes	Yes	Yes	Yes	Yes	Yes	Yes	Yes	Yes	Yes
**# of custom contrasts**		0	0	0	0	0	0	0	0	0	0	0	0	0	0	0	0	0	0	0
**Rest block**		No	No	No	No	No	No	No	No	No	No	No	No	No	No	No	No	No	No	No
**Design Efficiency**		0,05	0,05	0,05	0,05	0,05	0,05	0,05	0,05	0,05	0,05	0,05	0,05	0,05	0,05	0,05	0,05	0,05	0,05	0,05
**Detection Power**		0,8	0,8	0,8	0,8	0,8	0,8	0,8	0,8	0,8	0,8	0,8	0,8	0,8	0,8	0,8	0,8	0,8	0,8	0,8
**Trial probabilities**		0,1	0,1	0,1	0,1	0,1	0,1	0,1	0,1	0,1	0,1	0,1	0,1	0,1	0,1	0,1	0,1	0,1	0,1	0,1
**Psychological confounds**		0,05	0,05	0,05	0,05	0,05	0,05	0,05	0,05	0,05	0,05	0,05	0,05	0,05	0,05	0,05	0,05	0,05	0,05	0,05
**Order of confounding control**		3	3	3	3	3	3	3	3	3	3	3	3	3	3	3	3	3	3	3
**Max number of repeated stimulus types**		6	6	6	6	6	6	6	6	6	6	6	6	6	6	6	6	6	6	6
**Contrasts and probabilities**		Equal	Equal	Equal	Equal	Equal	Equal	Equal	Equal	Equal	Equal	Equal	Equal	Equal	Equal	Equal	Equal	Equal	Equal	Equal
**Run**		2	2	2	1	NA	2	1	1	1	1	1	1	1	1	1	1	1	1	1
**Detection Power**	Max	1,016	1,018	0,962	1,016	1,044	1,008	1,031	1,034	0,169	0,749	0,994	0,413	1,007	1,107	1,011	1,022	NA	0,922	0,999
**Estimation Efficiency**	Max	2,153	250,994	13,927	14,849	NA	24,656	23,856	0,000	372,120	21,413	1,589	3,029	1,013E+07	14,699	17,882	10,204	NA	8,000	0,897
**Psychological Confounding**	Max	0,872	0,898	0,887	0,898	NA	0,893	0,904	0,888	0,819	0,930	0,888	0,976	0,914	0,853	0,915	0,914	NA	0,862	0,958
**Stimulus Frequency**	Max	0,992	0,913	0,955	0,976	NA	0,992	0,992	0,992	0,835	0,960	0,976	0,856	0,976	0,981	0,909	0,955	NA	0,976	0,002
**Overall fit**	Max	1,024	13,351	1,495	1,567	NA	2,130	2,086	0,969	18,866	1,812	1,010	0,840	5,063E+05	1,761	1,586	1,459	NA	1,279	0,991

Design and Optimization Parameters

	Sim 1	Sim 2	Sim 3	Sim 4	Sim 5	Sim 6	Sim 7	Sim 8	Sim 9	Sim 10	Sim 11	Sim 12	Sim 13	Sim 14	Sim 15	Sim 16	Sim 17	Sim 18	Sim 19
**The assumed temporal autocorrelation coefficient**	0,3	0,3	0,3	0,3	0,3	0,3	0,3	0,3	0,3	0,3	0,3	0,3	0,3	0,3	0,3	0,3	0,3	0,3	0,3
**Do you want to optimize by using A-optimality or D-Optimality**	A	A	A	A	A	A	A	A	A	A	A	A	A	A	A	A	A	A	A
**The resolution of timing of stimuli**	0,25	0,25	0,25	0,25	0,25	0,25	0,25	0,25	0,25	0,25	0,25	0,25	0,25	0,25	0,25	0,25	0,25	0,25	0,25
**How many designs do you want to get?**	1	1	1	1	1	1	1	1	1	1	1	1	1	1	1	1	1	1	1
**Do you want to get hard limit on the probabilities?**	No	No	No	No	No	No	No	No	No	No	No	No	No	No	No	No	No	No	No

Genetic Algorithm Parameters

	Sim 1	Sim 2	Sim 3	Sim 4	Sim 5	Sim 6	Sim 7	Sim 8	Sim 9	Sim 10	Sim 11	Sim 12	Sim 13	Sim 14	Sim 15	Sim 16	Sim 17	Sim 18	Sim 19
**How many designs go from one generation to the next?**	20	20	20	20	20	20	20	20	20	20	20	20	20	20	20	20	20	20	20
**What percentage of the trials gets mutated?**	0.01	0.01	0.01	0.01	0.01	0.01	0.01	0.01	0.01	0.01	0.01	0.01	0.01	0.01	0.01	0.01	0.01	0.01	0.01
**How many immigrants per generation?**	4	4	4	4	4	4	4	4	4	4	4	4	4	4	4	4	4	4	4
**Number of generations (iterations or cycles)?**	100	100	100	100	100	100	100	100	100	100	100	100	100	100	100	100	100	100	100
**Number of generation in the prerun to define the maximum efficiency**	10	10	10	10	10	10	10	10	10	10	10	10	10	10	10	10	10	10	10
**Number of stable generations to reach convergence?**	1000	1000	1000	1000	1000	1000	1000	1000	1000	1000	1000	1000	1000	1000	1000	1000	1000	1000	1000

Design Experiment Genetic Algorithm Results

According to the web-based preliminary simulation results, the following two ITI value sets were found to be the most suitable scenarios:

- ITI min = 1 and ITI max = 3 and
- ITI min = 2 and ITI max = 4

To select the optimum one, the simulation was run with a higher number of generations, this time using the high-capacity university servers, rather than the web-based program. After all preliminary and final simulations were evaluated, it was decided to proceed with ITI min = 2 and ITI max = 4. The results of this scenario are in Supplementary Figure 12, Supplementary Figure 13, and Supplementary Table 9.

Supplementary Figure 12: Correlation Between Designs in Genetic Algorithm Result Report (http://www.neuropowertools.org)

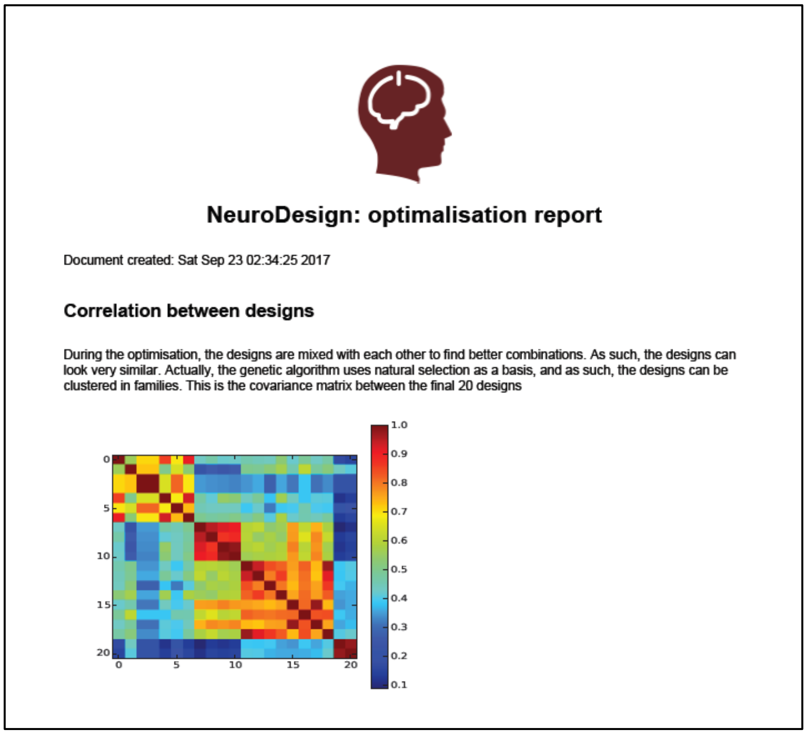

Supplementary Figure 13: Selected Designs in Genetic Algorithm Result Report

(http://www.neuropowertools.org)

Selected designs

The following figure shows in the upper panel the optimisation score over the different generations. Below are the expected signals of the best designs from different families, more specific and in relation with the covariance matrix, designs 0, 7, 19. Next to each design is the covariance matrix between the regressors, and the diagonalmatrix with the eigenvalues of the design matrix.

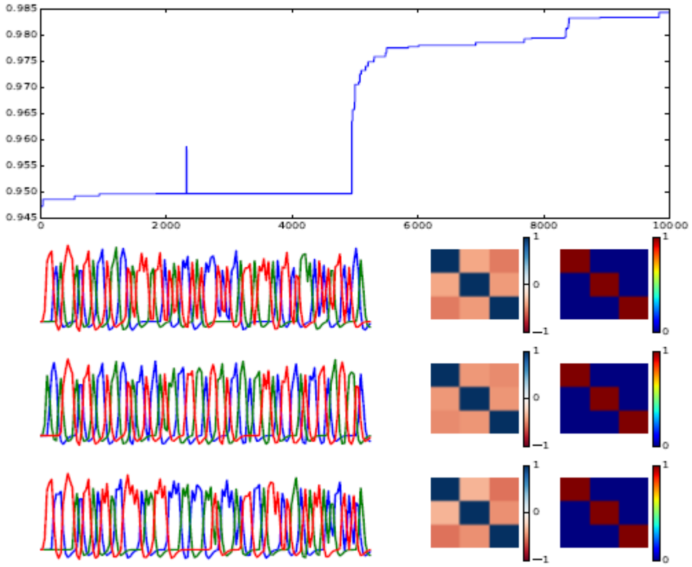

Supplementary Table 9: Genetic Algorithm Final Report (http://www.neuropowertools.org)

Experimental settings

Repetition time (TR):	3.0
Number of trials:	72
Number of scans:	144
Number of different stimuli:	3
Stimulus probabilities:	0.33 0.33 0.33
Duration of stimulus (s)	3.0
Seconds before stimulus (in trial):	0.0
Seconds after stimulus (in trial)	0.0
Duration of trial (s):	3.0
Total experiment duration(s):	432.0
Number of stimuli between rest blocks	0
Duration of rest blocks (s):	0.0
Contrasts:	0.5 -0.5 0.0<br>0.5 0.0 -0.5<br>0.0 0.5 -0.5<br>1.0 0.0 0.0<br>0.0 1.0 0.0<br>0.0 0.0 1.0<br>0.5 -0.5 0.0<br>0.5 0.0 -0.5<br>0.0 0.5 -0.5
ITI model:	uniform
minimum ITI:	2.0
mean ITI:	3.0
maximum ITI:	4.0
Hard probabilities:	False
Maximum number of repeated stimuli:	6
Resolution of design:	0.1
Assumed autocorrelation:	0.3

Optimalisation settings

Optimalisation weights (Fe,Fd,Fc,Ff):	0.05 0.8 0.1 0.05
Aoptimality?	True
Number of designs in each generation:	20
Number of immigrants in each generation:	4
Confounding order:	3
Convergence criterion:	1000
Number of precycles:	10000
Number of cycles:	10000
Percentage of mutations:	0.01
Seed:	1329817574

As mentioned above, the main goal of the final simulations is to determine the optimum ISI (or ITI) range and optimum stimulus sequence that will maximize detection power and prediction efficiency using the genetic algorithm. For this purpose, the program provides ITIs and the timing of the stimuli in the design in ".txt" format, as shown in Supplementary Table 10. However, these outputs are in raw data format and require processing as explained in the next section.

Supplementary Table 10: Simulation Raw Data

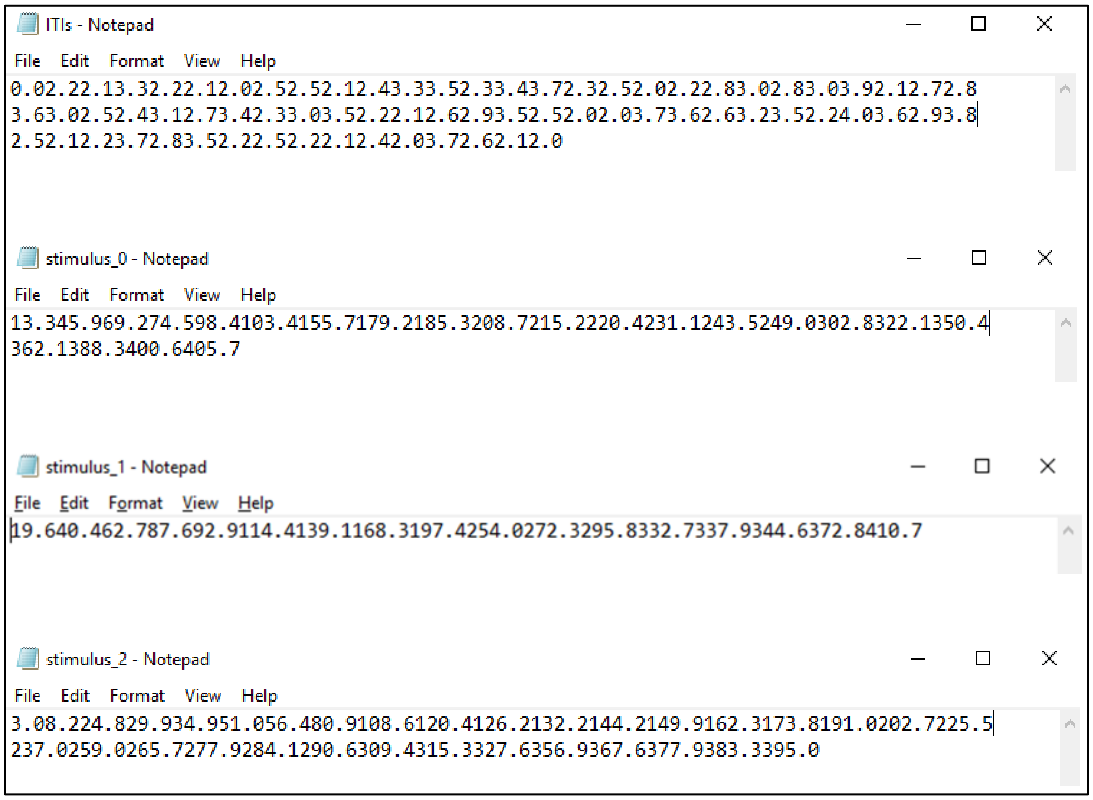
ITIs - Notepad

File Edit Format View Help

```
0.02.22.13.32.22.12.02.52.52.12.43.33.52.33.43.72.32.52.02.22.83.02.83.03.92.12.72.8
3.63.02.52.43.12.73.42.33.03.52.22.12.62.93.52.52.02.03.73.62.63.23.52.24.03.62.93.8
2.52.12.23.72.83.52.22.52.22.12.42.03.72.62.12.0
```

stimulus_0 - Notepad

File Edit Format View Help

```
13.345.969.274.598.4103.4155.7179.2185.3208.7215.2220.4231.1243.5249.0302.8322.1350.4
362.1388.3400.6405.7
```

stimulus_1 - Notepad

File Edit Format View Help

```
19.640.462.787.692.9114.4139.1168.3197.4254.0272.3295.8332.7337.9344.6372.8410.7
```

stimulus_2 - Notepad

File Edit Format View Help

```
3.08.224.829.934.951.056.480.9108.6120.4126.2132.2144.2149.9162.3173.8191.0202.7225.5
237.0259.0265.7277.9284.1290.6309.4315.3327.6356.9367.6377.9383.3395.0
```

Preparation of the Final Design Experiment Based on the Genetic Algorithm Results

The raw data obtained from the simulation were first converted into meaningful duration and time information under the headings of ITI and stimulus. Since there were three stimuli in this research, each was arranged as a separate time series. After the raw data preprocessing phase, it was arranged as in Supplementary Table 11.

After this pre-processing, the duration of each stimulus in seconds, the start time, and the end time were determined with decimal precision in the format shared in Supplementary Table 12. Here, the background images of Economy, Neutral, and Migration, which serve as our stimuli, are used as the basis, and the truck images are arranged in a fixed order. The duration of the images specified with Fixation in the table is arranged according to the ITIs, which are the program output. Numerical information about experimental design is shared in Supplementary Table 13. Accordingly, the economy stimulus which is Background 1 was repeated 22 times, the neutral stimulus which is Background 2 was repeated 17 times, and the migration stimulus which is Background 3 was repeated 33 times. Due to the sequence, each truck repeated 9 times. The same table also shows how many times the matching background and truck pairs are repeated.

According to these data, the final sequence was created in MS PowerPoint format, as shown in Supplementary Figure 14 (the starting section) and Supplementary Figure 15 (the complete design experiment). Subsequently, the image order and duration were shared with the software expert for uploading to the fMRI software. The program, which operates in conjunction with the fMRI device, presented these images to the participants in the intended order, with the specified ITIs, and at the designated timing during the test.

Supplementary Table 11: Pre-process Results of the Simulation Raw Data

No	ITI	Stimulus 1	Stimulus 2	Stimulus 3
1	0,00	13,30	19,60	3,00
2	2,20	45,90	40,40	8,20
3	2,10	69,20	62,70	24,80
4	3,30	74,50	87,60	29,90
5	2,20	98,40	92,90	34,90
6	2,10	103,40	114,40	51,00
7	2,00	155,70	139,10	56,40
8	2,50	179,20	168,30	80,90
9	2,50	185,30	197,40	108,60
10	2,10	208,70	254,00	120,40
11	2,40	215,20	272,30	126,20
12	3,30	220,40	295,80	132,20
13	3,50	231,10	332,70	144,20
14	2,30	243,50	337,90	149,90
15	3,40	249,00	344,60	162,30
16	3,70	302,80	372,80	173,80
17	2,30	322,10	410,70	191,00
18	2,50	350,40		202,70
19	2,00	362,10		225,50
20	2,20	388,30		237,00
21	2,80	400,60		259,00
22	3,00	405,70		265,70
23	2,80			277,90
24	3,00			284,10
25	3,90			290,60
26	2,10			309,40
27	2,70			315,30
28	2,80			327,60
29	3,60			356,90
30	3,00			367,60
31	2,50			377,90
32	2,40			383,30
33	3,10			395,00
34	2,70			
35	3,40			
36	2,30			
37	3,00			
38	3,50			
39	2,20			
40	2,10			
41	2,60			
42	2,90			
43	3,50			
44	2,50			
45	2,00			
46	2,00			
47	3,70			
48	3,60			
49	2,60			
50	3,20			
51	3,50			
52	2,20			
53	4,00			
54	3,60			
55	2,90			
56	3,80			
57	2,50			
58	2,10			
59	2,20			
60	3,70			
61	2,80			
62	3,50			
63	2,20			
64	2,50			
65	2,20			
66	2,10			
67	2,40			
68	2,00			
69	3,70			
70	2,60			
71	2,10			
72	2,00			

Supplementary Table 12: Design Experiment Prepared Based on the Simulation Data

	TD Duration (sec)	ITI (sec)	Ends at (sec)	Stimulus # (Fon#)	Kamyon #	Starts at (sec)	With +3 sec Starts at (sec)
Fixation		0,0	0,0				0,0
Trial 1	3,0		3,0	3	1	0,0	3,0
Fixation		2,2	5,2			3,0	6,0
Trial 2	3,0		8,2	3	2	5,2	8,2
Fixation		2,1	10,3			8,2	11,2
Trial 3	3,0		13,3	1	3	10,3	13,3
Fixation		3,3	16,6			13,3	16,3
Trial 4	3,0		19,6	2	4	16,6	19,6
Fixation		2,2	21,8			19,6	22,6
Trial 5	3,0		24,8	3	5	21,8	24,8
Fixation		2,1	26,9			24,8	27,8
Trial 6	3,0		29,9	3	6	26,9	29,9
Fixation		2,0	31,9			29,9	32,9
Trial 7	3,0		34,9	3	7	31,9	34,9
Fixation		2,5	37,4			34,9	37,9
Trial 8	3,0		40,4	2	8	37,4	40,4
Fixation		2,5	42,9			40,4	43,4
Trial 9	3,0		45,9	1	1	42,9	45,9
Fixation		2,1	48,0			45,9	48,9
Trial 10	3,0		51,0	3	2	48,0	51,0
Fixation		2,4	53,4			51,0	54,0
Trial 11	3,0		56,4	3	3	53,4	56,4
Fixation		3,3	59,7			56,4	59,4
Trial 12	3,0		62,7	2	4	59,7	62,7
Fixation		3,5	66,2			62,7	65,7
Trial 13	3,0		69,2	1	5	66,2	69,2
Fixation		2,3	71,5			69,2	72,2
Trial 14	3,0		74,5	1	6	71,5	74,5
Fixation		3,4	77,9			74,5	77,5
Trial 15	3,0		80,9	3	7	77,9	80,9
Fixation		3,7	84,6			80,9	83,9
Trial 16	3,0		87,6	2	8	84,6	87,6
Fixation		2,3	89,9			87,6	90,6
Trial 17	3,0		92,9	2	1	89,9	92,9
Fixation		2,5	95,4			92,9	95,9
Trial 18	3,0		98,4	1	2	95,4	98,4
Fixation		2,0	100,4			98,4	101,4
Trial 19	3,0		103,4	1	3	100,4	103,4
Fixation		2,2	105,6			103,4	106,4
Trial 20	3,0		108,6	3	4	105,6	108,6
Fixation		2,8	111,4			108,6	111,6
Trial 21	3,0		114,4	2	5	111,4	114,4
Fixation		3,0	117,4			114,4	117,4
Trial 22	3,0		120,4	3	6	117,4	120,4
Fixation		2,8	123,2			120,4	123,4
Trial 23	3,0		126,2	3	7	123,2	126,2
Fixation		3,0	129,2			126,2	129,2
Trial 24	3,0		132,2	3	8	129,2	132,2

Supplementary Table 12: Design Experiment Prepared Based on the Simulation Data (continued))

Fixation		3,9	136,1			132,2	135,2
Trial 25	3,0		139,1	2	1	136,1	139,1
Fixation		2,1	141,2			139,1	142,1
Trial 26	3,0		144,2	3	2	141,2	144,2
Fixation		2,7	146,9			144,2	147,2
Trial 27	3,0		149,9	3	3	146,9	149,9
Fixation		2,8	152,7			149,9	152,9
Trial 28	3,0		155,7	1	4	152,7	155,7
Fixation		3,6	159,3			155,7	158,7
Trial 29	3,0		162,3	3	5	159,3	162,3
Fixation		3,0	165,3			162,3	165,3
Trial 30	3,0		168,3	2	6	165,3	168,3
Fixation		2,5	170,8			168,3	171,3
Trial 31	3,0		173,8	3	7	170,8	173,8
Fixation		2,4	176,2			173,8	176,8
Trial 32	3,0		179,2	1	8	176,2	179,2
Fixation		3,1	182,3			179,2	182,2
Trial 33	3,0		185,3	1	1	182,3	185,3
Fixation		2,7	188,0			185,3	188,3
Trial 34	3,0		191,0	3	2	188,0	191,0
Fixation		3,4	194,4			191,0	194,0
Trial 35	3,0		197,4	2	3	194,4	197,4
Fixation		2,3	199,7			197,4	200,4
Trial 36	3,0		202,7	3	4	199,7	202,7
Fixation		3,0	205,7			202,7	205,7
Trial 37	3,0		208,7	1	5	205,7	208,7
Fixation		3,5	212,2			208,7	211,7
Trial 38	3,0		215,2	1	6	212,2	215,2
Fixation		2,2	217,4			215,2	218,2
Trial 39	3,0		220,4	1	7	217,4	220,4
Fixation		2,1	222,5			220,4	223,4
Trial 40	3,0		225,5	3	8	222,5	225,5
Fixation		2,6	228,1			225,5	228,5
Trial 41	3,0		231,1	1	1	228,1	231,1
Fixation		2,9	234,0			231,1	234,1
Trial 42	3,0		237,0	3	2	234,0	237,0
Fixation		3,5	240,5			237,0	240,0
Trial 43	3,0		243,5	1	3	240,5	243,5
Fixation		2,5	246,0			243,5	246,5
Trial 44	3,0		249,0	1	4	246,0	249,0
Fixation		2,0	251,0			249,0	252,0
Trial 45	3,0		254,0	2	5	251,0	254,0
Fixation		2,0	256,0			254,0	257,0
Trial 46	3,0		259,0	3	6	256,0	259,0
Fixation		3,7	262,7			259,0	262,0
Trial 47	3,0		265,7	3	7	262,7	265,7
Fixation		3,6	269,3			265,7	268,7
Trial 48	3,0		272,3	2	8	269,3	272,3

Supplementary Table 12: Design Experiment Prepared Based on the Simulation Data (continued)

Fixation		2,6	274,9			272,3	275,3
Trial 49	3,0		277,9	3	1	274,9	277,9
Fixation		3,2	281,1			277,9	280,9
Trial 50	3,0		284,1	3	2	281,1	284,1
Fixation		3,5	287,6			284,1	287,1
Trial 51	3,0		290,6	3	3	287,6	290,6
Fixation		2,2	292,8			290,6	293,6
Trial 52	3,0		295,8	2	4	292,8	295,8
Fixation		4,0	299,8			295,8	298,8
Trial 53	3,0		302,8	1	5	299,8	302,8
Fixation		3,6	306,4			302,8	305,8
Trial 54	3,0		309,4	3	6	306,4	309,4
Fixation		2,9	312,3			309,4	312,4
Trial 55	3,0		315,3	3	7	312,3	315,3
Fixation		3,8	319,1			315,3	318,3
Trial 56	3,0		322,1	1	8	319,1	322,1
Fixation		2,5	324,6			322,1	325,1
Trial 57	3,0		327,6	3	1	324,6	327,6
Fixation		2,1	329,7			327,6	330,6
Trial 58	3,0		332,7	2	2	329,7	332,7
Fixation		2,2	334,9			332,7	335,7
Trial 59	3,0		337,9	2	3	334,9	337,9
Fixation		3,7	341,6			337,9	340,9
Trial 60	3,0		344,6	2	4	341,6	344,6
Fixation		2,8	347,4			344,6	347,6
Trial 61	3,0		350,4	1	5	347,4	350,4
Fixation		3,5	353,9			350,4	353,4
Trial 62	3,0		356,9	3	6	353,9	356,9
Fixation		2,2	359,1			356,9	359,9
Trial 63	3,0		362,1	1	7	359,1	362,1
Fixation		2,5	364,6			362,1	365,1
Trial 64	3,0		367,6	3	8	364,6	367,6
Fixation		2,2	369,8			367,6	370,6
Trial 65	3,0		372,8	2	1	369,8	372,8
Fixation		2,1	374,9			372,8	375,8
Trial 66	3,0		377,9	3	2	374,9	377,9
Fixation		2,4	380,3			377,9	380,9
Trial 67	3,0		383,3	3	3	380,3	383,3
Fixation		2,0	385,3			383,3	386,3
Trial 68	3,0		388,3	1	4	385,3	388,3
Fixation		3,7	392,0			388,3	391,3
Trial 69	3,0		395,0	3	5	392,0	395,0
Fixation		2,6	397,6			395,0	398,0
Trial 70	3,0		400,6	1	6	397,6	400,6
Fixation		2,1	402,7			400,6	403,6
Trial 71	3,0		405,7	1	7	402,7	405,7
Fixation		2,0	407,7			405,7	408,7
Trial 72	3,0		410,7	2	8	407,7	410,7

Supplementary Table 13: Design Experiment Summary

		Design Experiment	Theoretical
Fon 1	Economy	22	24
Fon 2	Neutral	17	24
Fon 3	Migration	33	24
		72	72
		Design Experiment	Theoretical
	Truck 1	9	9
	Truck 2	9	9
	Truck 3	9	9
	Truck 4	9	9
	Truck 5	9	9
	Truck 6	9	9
	Truck 7	9	9
	Truck 8	9	9
		72	72
		Design Experiment	Theoretical
Fon 1	Truck 1	3	3
	Truck 2	1	3
	Truck 3	3	3
	Truck 4	3	3
	Truck 5	4	3
	Truck 6	3	3
	Truck 7	3	3
	Truck 8	2	3
		22	24
Fon 2	Truck 1	3	3
	Truck 2	1	3
	Truck 3	2	3
	Truck 4	4	3
	Truck 5	2	3
	Truck 6	1	3
	Truck 7	0	3
	Truck 8	4	3
		17	24
Fon 3	Truck 1	3	3
	Truck 2	7	3
	Truck 3	4	3
	Truck 4	2	3
	Truck 5	3	3
	Truck 6	5	3
	Truck 7	6	3
	Truck 8	3	3
		33	24

Supplementary Figure 14: The Starting Section of the Design Experiment (translated)

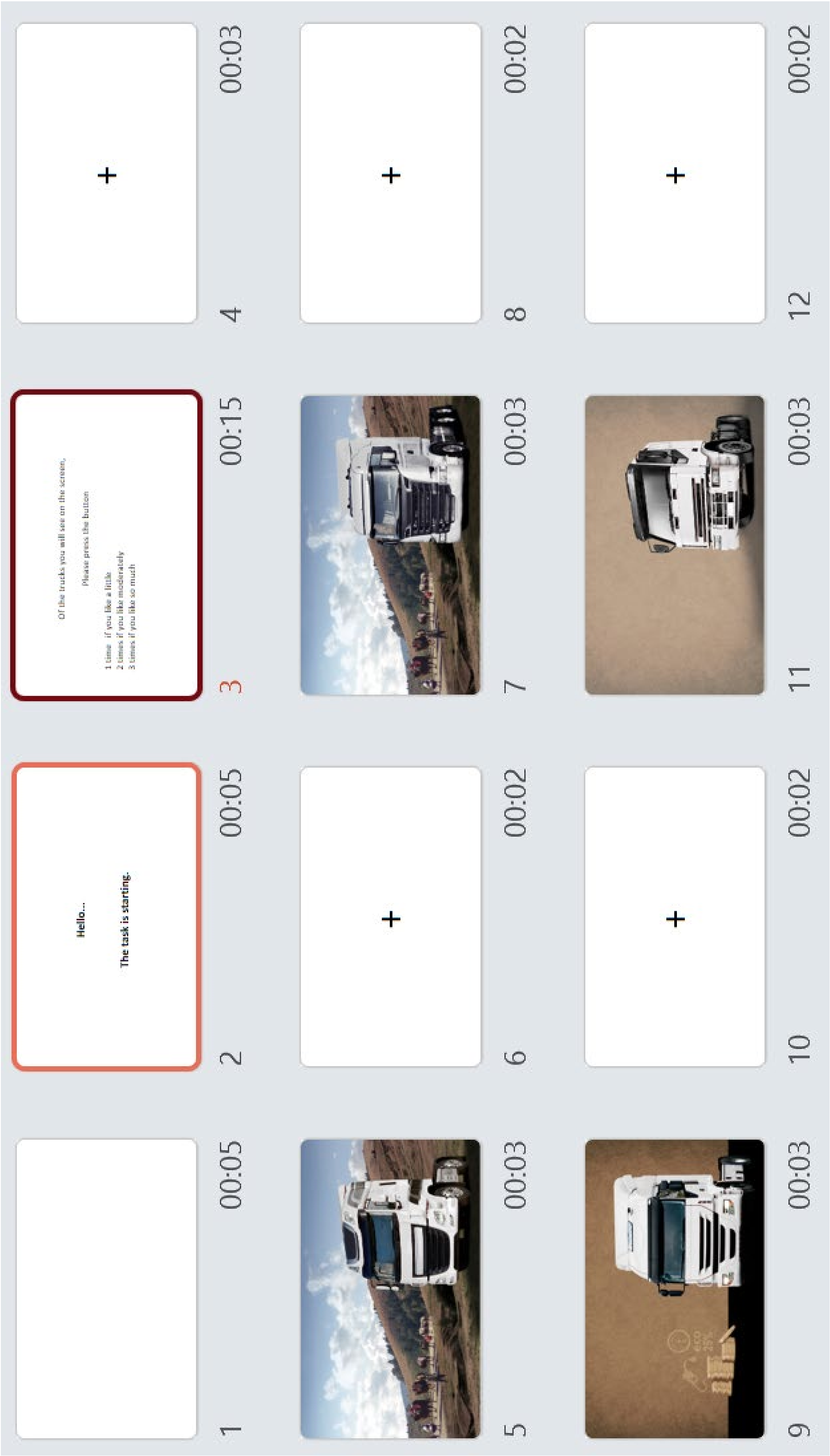

Supplementary Figure 15: The Complete Design Experiment (the original file without translation)

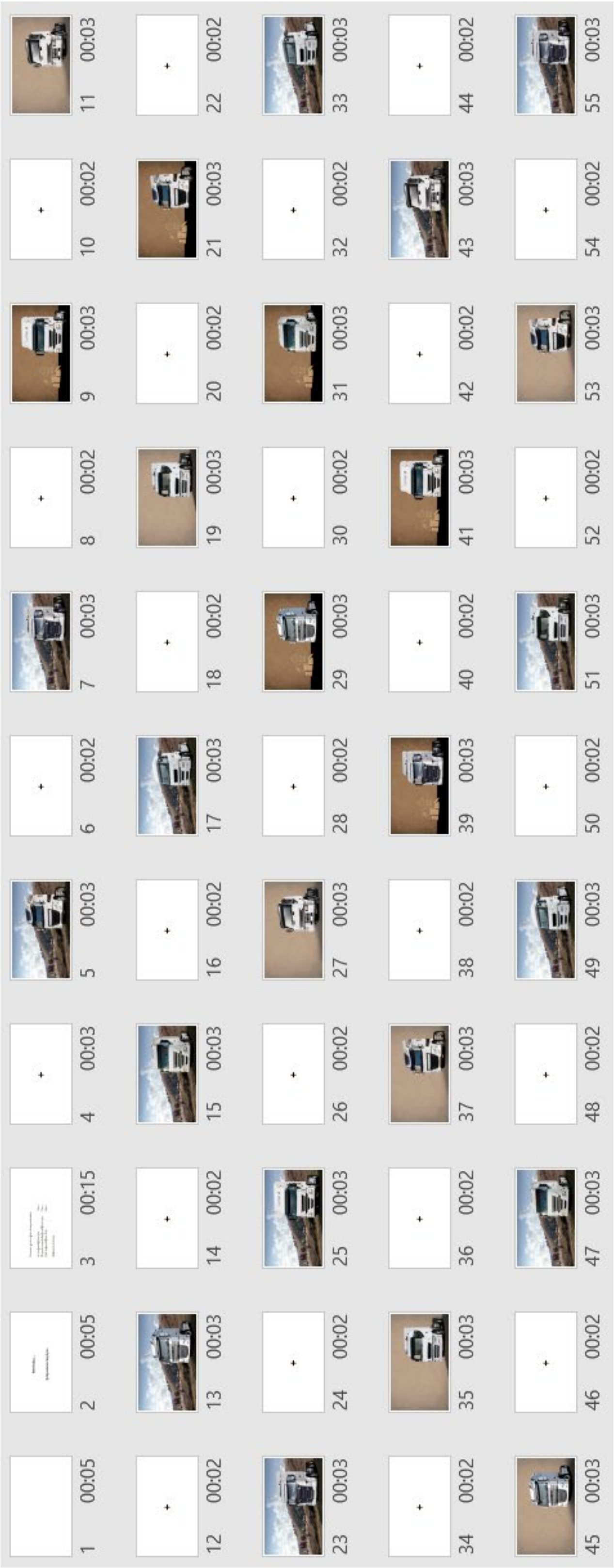

Supplementary Figure 15: The Complete Design Experiment (the original file without translation) (continued)

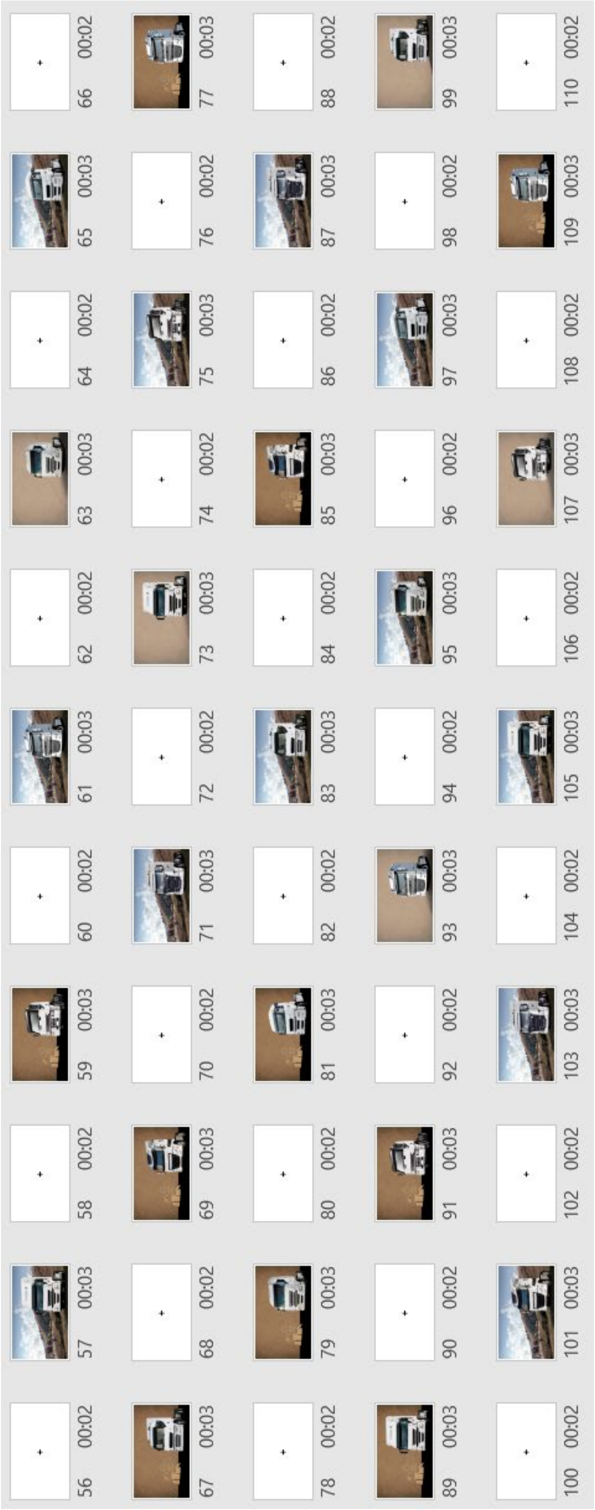

Supplementary Figure 15: The Complete Design Experiment (the original file without translation) (continued)

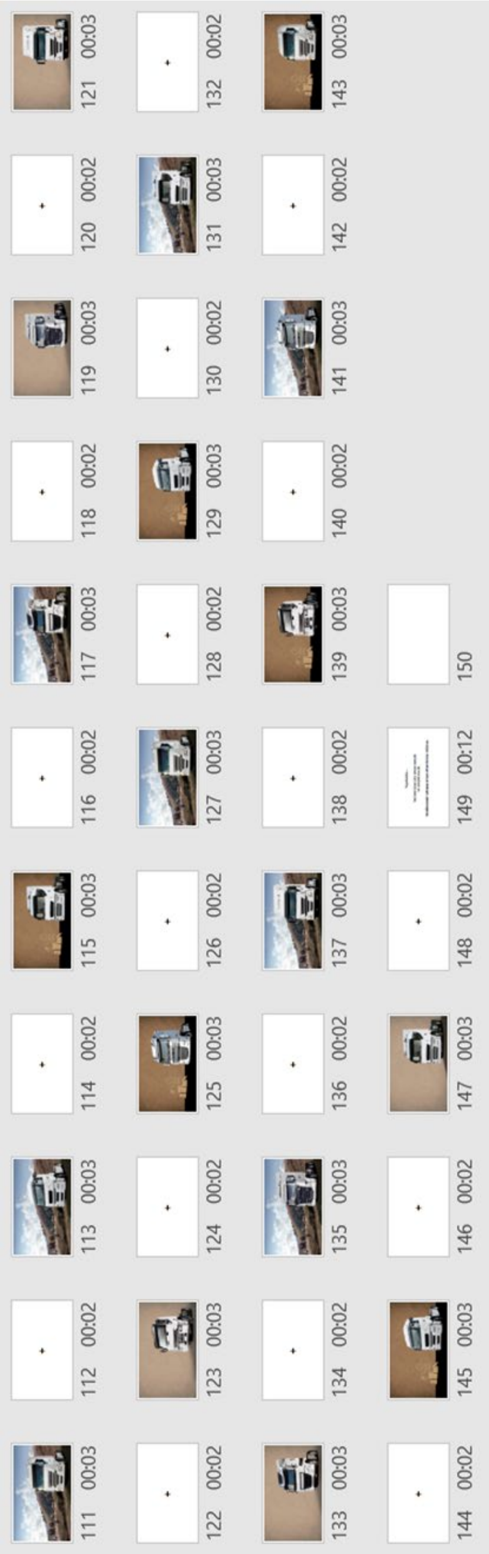

Supplementary Material K: fMRI Task Results

Supplementary Table 14: Background 1 (F1) Average

CORTICAL

Label	Left Cluster Size	z-stats	Coordinates x y z	Right Cluster Size	z-stats	Coordinates x y z
Frontal Pole	589.0	4.662	-34 44 10	395.0	4.827	38 36 12
Insular Cortex	225.0	4.631	-32 14 2	122.0	4.690	34 20 0
Superior Frontal Gyrus	43.0	3.368	-2 14 58	25.0	3.319	2 10 62
Middle Frontal Gyrus	382.0	5.540	-40 28 22	121.0	3.824	46 6 40
Inferior Frontal Gyrus, pars triangularis	70.0	5.023	-40 28 20	14.0	3.541	42 32 14
Inferior Frontal Gyrus, pars opercularis	191.0	4.646	-40 10 24	118.0	3.844	44 8 24
Precentral Gyrus	1466.0	5.943	-34 -24 46	335.0	5.577	42 6 26
Inferior Temporal Gyrus, posterior division	0.0	0.000	0 0 0	9.0	2.923	54 -38 -22
Inferior Temporal Gyrus, temporooccipital part	73.0	4.378	-42 -62 -10	83.0	4.938	46 -60 -14
Postcentral Gyrus	1132.0	6.434	-44 -20 54	40.0	3.134	56 -14 34
Superior Parietal Lobule	277.0	4.213	-28 -46 42	70.0	3.637	38 -50 42
Supramarginal Gyrus, anterior division	204.0	5.642	-50 -30 42	31.0	3.138	46 -32 40
Supramarginal Gyrus, posterior division	69.0	4.182	-40 -46 42	106.0	3.877	46 -40 42
Angular Gyrus	32.0	3.204	-38 -58 44	40.0	3.888	38 -50 40
Lateral Occipital Cortex, superior division	688.0	5.393	-24 -68 38	730.0	5.072	28 -64 36
Lateral Occipital Cortex, inferior division	699.0	6.090	-28 -90 -4	726.0	6.825	28 -88 -4
Intracalcarine Cortex	17.0	4.027	-12 -88 2	42.0	4.875	12 -88 2
Juxtapositional Lobule Cortex	594.0	6.205	-2 -6 54	324.0	5.005	6 4 52
Paracingulate Gyrus	448.0	4.970	-4 8 44	320.0	4.467	6 16 42
Cingulate Gyrus, anterior division	387.0	5.212	-4 4 44	296.0	4.648	6 16 36
Cingulate Gyrus, posterior division	6.0	3.610	-4 -16 46	0.0	0.000	0 0 0
Precuneous Cortex	6.0	3.235	-18 -66 30	8.0	2.953	22 -60 30
Cuneal Cortex	2.0	2.447	-20 -70 24	0.0	0.000	0 0 0
Frontal Orbital Cortex	48.0	4.333	-30 26 -6	26.0	3.379	32 26 -4
Parahippocampal Gyrus, posterior division	0.0	0.000	0 0 0	7.0	2.835	26 -34 -18
Lingual Gyrus	239.0	6.471	-12 -88 -10	508.0	6.041	12 -84 -12
Temporal Fusiform Cortex, posterior division	199.0	5.195	-36 -44 -24	177.0	5.239	30 -36 -26
Temporal Occipital Fusiform Cortex	489.0	6.007	-40 -64 -20	637.0	6.144	32 -44 -24
Occipital Fusiform Gyrus	888.0	6.609	-24 -88 -10	880.0	6.658	26 -86 -6
Frontal Operculum Cortex	109.0	4.247	-42 16 -2	13.0	2.768	32 20 10
Central Opercular Cortex	119.0	3.568	-44 4 6	6.0	2.746	40 4 16
Parietal Operculum Cortex	4.0	3.172	-42 -28 24	0.0	0.000	0 0 0
Occipital Pole	1054.0	8.199	-12 -96 -8	963.0	6.715	18 -96 2

SUB-CORTICAL

Label	Left Cluster Size	z-stats	Coordinates x y z	Right Cluster Size	z-stats	Coordinates x y z
Left Cerebral White Matter	6183.0	6.804	-14 -98 2	0.0	0.000	0 0 0
Left Cerebral Cortex	10553.0	8.199	-12 -96 -8	33.0	4.320	2 -96 -8
Left Lateral Ventrical	11.0	3.838	-16 26 10	0.0	0.000	0 0 0
Left Thalamus	5.0	2.715	-12 -4 8	0.0	0.000	0 0 0
Left Caudate	33.0	3.446	-16 4 16	0.0	0.000	0 0 0
Left Putamen	234.0	3.658	-26 -2 -6	0.0	0.000	0 0 0
Left Pallidum	57.0	3.392	-24 -4 -2	0.0	0.000	0 0 0
Brain-Stem	2.0	2.571	-28 -36 -32	14.0	3.477	26 -38 -30
Right Cerebral White Matter	0.0	0.000	0 0 0	4157.0	6.817	26 -88 -4
Right Cerebral Cortex	28.0	4.086	-2 -80 -22	7578.0	6.852	26 -88 -6
Right Lateral Ventricle	0.0	0.000	0 0 0	4.0	2.509	16 24 10
Right Thalamus	0.0	0.000	0 0 0	2.0	2.429	10 0 8
Right Caudate	0.0	0.000	0 0 0	18.0	2.791	16 18 2
Right Putamen	0.0	0.000	0 0 0	43.0	3.284	18 8 2
Right Pallidum	0.0	0.000	0 0 0	15.0	3.406	16 6 2

Supplementary Table 15: Background 2 (F2) Average

CORTICAL

Label	Left Cluster Size	z-stats	Coordinates x y z	Right Cluster Size	z-stats	Coordinates x y z
Frontal Pole	365.0	4.387	-24 44 16	430.0	4.814	38 38 12
Insular Cortex	162.0	4.377	-28 22 -2	142.0	5.010	34 20 -2
Superior Frontal Gyrus	22.0	3.322	-12 2 64	19.0	3.102	2 10 62
Middle Frontal Gyrus	249.0	4.615	-38 28 22	136.0	4.084	46 6 40
Inferior Frontal Gyrus, pars triangularis	41.0	4.195	-38 28 20	17.0	3.408	44 34 12
Inferior Frontal Gyrus, pars opercularis	72.0	4.790	-40 10 24	168.0	4.575	48 12 26
Precentral Gyrus	1275.0	6.008	-42 -20 56	439.0	5.501	42 6 26
Inferior Temporal Gyrus, posterior division	0.0	0.000	0 0 0	9.0	3.067	54 -38 -22
Inferior Temporal Gyrus, temporooccipital part	73.0	4.294	-46 -64 -16	122.0	5.219	46 -60 -16
Postcentral Gyrus	1091.0	6.181	-44 -20 52	0.0	0.000	0 0 0
Superior Parietal Lobule	233.0	4.469	-28 -46 40	73.0	3.852	30 -54 42
Supramarginal Gyrus, anterior division	158.0	5.019	-50 -30 42	27.0	3.150	48 -32 42
Supramarginal Gyrus, posterior division	63.0	4.333	-40 -46 42	77.0	3.443	46 -40 42
Angular Gyrus	30.0	3.592	-36 -58 40	43.0	4.469	36 -54 38
Lateral Occipital Cortex, superior division	560.0	5.315	-26 -64 38	765.0	5.145	34 -80 12
Lateral Occipital Cortex, inferior division	718.0	6.562	-28 -90 -4	788.0	6.629	30 -86 -6
Intracalcarine Cortex	22.0	4.399	-8 -90 -2	12.0	3.840	12 -88 2
Juxtapositional Lobule Cortex	561.0	6.338	-4 -4 54	343.0	4.988	6 4 52
Paracingulate Gyrus	352.0	5.044	-6 18 38	305.0	4.873	6 18 38
Cingulate Gyrus, anterior division	436.0	5.020	-4 12 40	356.0	5.134	6 18 36
Cingulate Gyrus, posterior division	10.0	3.781	-4 -16 46	0.0	0.000	0 0 0
Precuneous Cortex	6.0	3.293	-18 -66 30	19.0	3.196	20 -60 32
Frontal Orbital Cortex	39.0	4.456	-28 26 -6	24.0	3.513	32 24 -6
Parahippocampal Gyrus, posterior division	0.0	0.000	0 0 0	7.0	2.849	24 -34 -20
Lingual Gyrus	243.0	6.541	-12 -88 -12	406.0	5.893	12 -86 -14
Temporal Fusiform Cortex, posterior division	168.0	5.328	-34 -44 -22	145.0	5.137	30 -36 -26
Temporal Occipital Fusiform Cortex	515.0	5.553	-36 -64 -14	681.0	6.282	32 -44 -24
Occipital Fusiform Gyrus	894.0	6.511	-14 -90 -12	874.0	6.207	38 -72 -14
Frontal Operculum Cortex	54.0	3.596	-44 12 -2	32.0	3.137	40 14 6
Central Opercular Cortex	29.0	3.030	-46 8 0	2.0	2.457	40 4 16
Parietal Operculum Cortex	2.0	2.745	-42 -28 24	0.0	0.000	0 0 0
Occipital Pole	1025.0	8.037	-12 -96 -6	899.0	6.607	16 -96 2

SUB-CORTICAL

Label	Left Cluster Size	z-stats	Coordinates x y z	Right Cluster Size	z-stats	Coordinates x y z
Left Cerebral White Matter	4865.0	7.235	-14 -98 2	0.0	0.000	0 0 0
Left Cerebral Cortex	9381.0	8.037	-12 -96 -6	28.0	4.033	2 -96 -8
Left Thalamus	3.0	2.382	-10 -4 6	0.0	0.000	0 0 0
Left Putamen	147.0	3.181	-22 2 0	0.0	0.000	0 0 0
Left Pallidum	35.0	3.094	-20 2 0	0.0	0.000	0 0 0
Brain-Stem	0.0	0.000	0 0 0	10.0	3.263	26 -36 -30
Right Cerebral White Matter	0.0	0.000	0 0 0	4162.0	6.602	30 -84 -4
Right Cerebral Cortex	20.0	3.330	0 -82 -20	7591.0	6.629	30 -86 -6
Right Caudate	0.0	0.000	0 0 0	3.0	2.617	16 18 -2
Right Putamen	0.0	0.000	0 0 0	77.0	3.051	22 12 -4
Right Pallidum	0.0	0.000	0 0 0	10.0	2.750	14 4 2

Supplementary Table 16: Background 3 (F3) Average

CORTICAL

Label	Left Cluster Size	z-stats	Coordinates x y z	Right Cluster Size	z-stats	Coordinates x y z
Frontal Pole	682.0	5.201	-34 48 10	396.0	4.628	38 38 14
Insular Cortex	282.0	5.627	-32 14 2	134.0	4.841	34 20 -2
Superior Frontal Gyrus	73.0	4.109	0 12 64	36.0	3.976	2 10 62
Middle Frontal Gyrus	404.0	5.534	-40 28 22	202.0	4.067	44 32 18
Inferior Frontal Gyrus, pars triangularis	76.0	5.100	-42 26 22	16.0	3.666	44 32 16
Inferior Frontal Gyrus, pars opercularis	184.0	5.133	-40 10 24	151.0	3.933	50 12 26
Precentral Gyrus	1682.0	6.742	-34 -24 46	454.0	5.351	42 6 26
Inferior Temporal Gyrus, temporooccipital part	90.0	4.284	-44 -64 -12	107.0	5.032	46 -60 -16
Postcentral Gyrus	1237.0	6.231	-36 -24 44	5.0	2.764	40 -32 40
Superior Parietal Lobule	382.0	5.118	-28 -46 40	117.0	4.239	38 -50 42
Supramarginal Gyrus, anterior division	239.0	6.080	-50 -30 42	51.0	3.630	48 -32 42
Supramarginal Gyrus, posterior division	85.0	4.619	-40 -46 42	155.0	4.123	46 -42 42
Angular Gyrus	68.0	4.135	-40 -56 44	102.0	4.533	38 -50 40
Lateral Occipital Cortex, superior division	912.0	6.112	-24 -68 38	1007.0	5.705	34 -80 12
Lateral Occipital Cortex, inferior division	795.0	6.444	-28 -90 -4	775.0	6.880	30 -86 -6
Intracalcarine Cortex	24.0	4.304	-8 -90 -2	155.0	5.637	10 -86 -2
Juxtapositional Lobule Cortex	618.0	6.292	-4 -4 54	403.0	4.900	6 4 54
Paracingulate Gyrus	526.0	5.429	-4 8 44	346.0	5.006	6 20 36
Cingulate Gyrus, anterior division	563.0	5.369	-4 4 44	403.0	5.115	4 18 36
Cingulate Gyrus, posterior division	15.0	4.004	-4 -16 46	0.0	0.000	0 0 0
Precuneous Cortex	50.0	4.074	-18 -66 30	35.0	3.776	20 -60 30
Cuneal Cortex	6.0	2.858	-20 -70 24	0.0	0.000	0 0 0
Frontal Orbital Cortex	85.0	4.720	-30 26 -6	31.0	3.310	28 20 -8
Parahippocampal Gyrus, posterior division	10.0	3.398	-24 -42 -14	50.0	4.097	24 -34 -18
Lingual Gyrus	363.0	6.739	-8 -90 -10	812.0	7.332	12 -84 -10
Temporal Fusiform Cortex, posterior division	216.0	5.045	-34 -44 -22	191.0	5.106	30 -36 -26
Temporal Occipital Fusiform Cortex	564.0	5.975	-28 -60 -16	702.0	5.995	36 -62 -16
Occipital Fusiform Gyrus	909.0	6.678	-34 -76 -18	880.0	6.838	26 -86 -6
Frontal Operculum Cortex	114.0	4.709	-44 12 -2	39.0	3.123	44 10 6
Central Opercular Cortex	156.0	4.105	-56 -18 20	26.0	3.345	44 8 6
Parietal Operculum Cortex	5.0	3.042	-42 -28 24	0.0	0.000	0 0 0
Supracalcarine Cortex	2.0	2.572	-22 -64 20	0.0	0.000	0 0 0
Occipital Pole	1082.0	8.319	-12 -96 -6	1000.0	7.019	18 -96 2

SUB-CORTICAL

Label	Left Cluster Size	z-stats	Coordinates x y z	Right Cluster Size	z-stats	Coordinates x y z
Left Cerebral White Matter	7498.0	6.760	-14 -98 2	0.0	0.000	0 0 0
Left Cerebral Cortex	12342.0	8.319	-12 -96 -6	32.0	3.913	2 -88 -18
Left Lateral Ventrical	22.0	3.603	-16 26 10	0.0	0.000	0 0 0
Left Thalamus	17.0	3.175	-12 -4 8	0.0	0.000	0 0 0
Left Caudate	99.0	3.966	-16 4 16	0.0	0.000	0 0 0
Left Putamen	260.0	4.029	-22 4 2	0.0	0.000	0 0 0
Left Pallidum	71.0	3.766	-20 2 2	0.0	0.000	0 0 0
Brain-Stem	0.0	0.000	0 0 0	24.0	3.706	26 -36 -30
Right Cerebral White Matter	0.0	0.000	0 0 0	4872.0	7.001	28 -86 -6
Right Cerebral Cortex	41.0	4.955	-2 -80 -22	9264.0	7.332	12 -84 -10
Right Caudate	0.0	0.000	0 0 0	33.0	2.916	18 24 6
Right Putamen	0.0	0.000	0 0 0	107.0	3.655	18 8 2
Right Pallidum	0.0	0.000	0 0 0	19.0	3.550	16 6 2

Supplementary Table 17: Background 1 > Background 2 (F1 > F2)

CORTICAL

Label	Left Cluster Size	z-stats	Coordinates x y z	Right Cluster Size	z-stats	Coordinates x y z
Intracalcarine Cortex	0.0	0.000	0 0 0	102.0	4.801	14 -80 2
Lingual Gyrus	0.0	0.000	0 0 0	234.0	4.740	14 -74 -2
Occipital Fusiform Gyrus	0.0	0.000	0 0 0	3.0	2.737	16 -74 -10
Occipital Pole	0.0	0.000	0 0 0	6.0	2.600	16 -90 2

SUB-CORTICAL

Label	Left Cluster Size	z-stats	Coordinates x y z	Right Cluster Size	z-stats	Coordinates x y z
Right Cerebral White Matter	0.0	0.000	0 0 0	151.0	4.801	14 -80 2
Right Cerebral Cortex	0.0	0.000	0 0 0	308.0	4.675	12 -76 -4

Supplementary Table 18: Background 3 > Background 1 (F3 > F1)

CORTICAL

Label	Left Cluster Size	z-stats	Coordinates x y z	Right Cluster Size	z-stats	Coordinates x y z
Middle Temporal Gyrus, temporooccipital part	0.0	0.000	0 0 0	4.0	2.684	40 -56 12
Postcentral Gyrus	0.0	0.000	0 0 0	3.0	2.592	12 -38 50
Angular Gyrus	0.0	0.000	0 0 0	20.0	3.111	48 -58 32
Lateral Occipital Cortex, superior division	231.0	3.152	-38 -80 22	478.0	3.815	34 -76 20
Lateral Occipital Cortex, inferior division	31.0	3.001	-42 -72 14	29.0	2.724	44 -68 14
Intracalcarine Cortex	72.0	3.250	0 -80 4	499.0	5.701	12 -82 0
Cingulate Gyrus, posterior division	107.0	3.268	-10 -44 -2	203.0	4.001	6 -36 46
Precuneous Cortex	622.0	4.088	-20 -58 4	464.0	3.971	2 -60 48
Cuneal Cortex	162.0	3.797	-4 -74 24	195.0	3.834	4 -80 26
Parahippocampal Gyrus, posterior division	116.0	5.109	-20 -38 -16	72.0	3.607	22 -38 -14
Lingual Gyrus	938.0	4.638	-20 -42 -14	1356.0	5.748	6 -80 -4
Temporal Fusiform Cortex, posterior division	40.0	4.277	-24 -38 -18	9.0	3.470	22 -38 -18
Temporal Occipital Fusiform Cortex	47.0	3.944	-22 -44 -16	97.0	4.506	22 -48 -14
Occipital Fusiform Gyrus	78.0	3.823	-18 -70 -14	77.0	4.009	20 -70 -12
Supracalcarine Cortex	37.0	3.261	0 -74 20	107.0	3.935	4 -78 12
Occipital Pole	91.0	3.409	-8 -92 20	238.0	4.269	8 -90 -4

SUB-CORTICAL

Label	Left Cluster Size	z-stats	Coordinates x y z	Right Cluster Size	z-stats	Coordinates x y z
Left Cerebral White Matter	426.0	3.586	-10 -86 -8	0.0	0.000	0 0 0
Left Cerebral Cortex	2944.0	5.109	-20 -38 -16	1.0	2.527	2 -86 40
Brain-Stem	1.0	2.366	-8 -38 -8	42.0	2.982	20 -30 -26
Right Cerebral White Matter	0.0	0.000	0 0 0	833.0	5.622	12 -80 0
Right Cerebral Cortex	16.0	3.653	0 -68 -8	4152.0	5.748	6 -80 -4

Supplementary Table 19: Background 3 > Background 2 (F3 > F3)

CORTICAL

Label	Left Cluster Size	z-stats	Coordinates x y z	Right Cluster Size	z-stats	Coordinates x y z
Lateral Occipital Cortex, superior division	0.0	0.000	0 0 0	28.0	3.402	22 -84 24
Intracalcarine Cortex	53.0	3.107	-16 -62 6	419.0	6.060	12 -82 2
Cingulate Gyrus, posterior division	11.0	3.222	-18 -50 2	13.0	3.452	18 -48 2
Precuneous Cortex	142.0	3.661	-20 -58 6	86.0	3.555	20 -56 10
Cuneal Cortex	35.0	3.188	0 -80 36	33.0	2.834	10 -86 32
Parahippocampal Gyrus, posterior division	63.0	4.270	-24 -42 -14	12.0	3.087	22 -38 -16
Lingual Gyrus	770.0	4.684	-8 -82 -10	915.0	6.054	14 -82 -6
Temporal Fusiform Cortex, posterior division	28.0	4.335	-26 -42 -14	7.0	3.141	24 -38 -18
Temporal Occipital Fusiform Cortex	15.0	3.802	-24 -44 -14	51.0	3.276	22 -40 -18
Occipital Fusiform Gyrus	37.0	3.302	-14 -80 -10	133.0	4.482	16 -74 -12
Supracalcarine Cortex	13.0	2.593	0 -74 18	34.0	3.170	20 -66 16
Occipital Pole	17.0	3.136	-2 -90 28	221.0	4.605	12 -90 -2

SUB-CORTICAL

Label	Left Cluster Size	z-stats	Coordinates x y z	Right Cluster Size	z-stats	Coordinates x y z
Left Cerebral White Matter	120.0	3.670	-10 -86 -8	0.0	0.000	0 0 0
Left Cerebral Cortex	1566.0	4.684	-8 -82 -10	1.0	2.358	2 -86 40
Right Cerebral White Matter	0.0	0.000	0 0 0	551.0	6.054	14 -82 -6
Right Cerebral Cortex	18.0	3.070	0 -76 -14	2258.0	6.060	12 -82 2

Supplementary Table 20: Comparison of Backgrounds in Terms of Brain Activations

CORTICAL			
**LABEL**	**FON1 > FON 2**	**FON 3 > FON 1**	**FON 3 > FON 2**
Frontal Pole			
Insular Cortex			
Superior Frontal Gyrus			
Middle Frontal Gyrus			
Inferior Frontal Gyrus, pars triangularis			
Inferior Frontal Gyrus, pars opercularis			
Precentral Gyrus			
Inferior Temporal Gyrus, posterior division			
Inferior Temporal Gyrus, temporooccipital part			
Postcentral Gyrus		√	
Superior Parietal Lobule			
Supramarginal Gyrus, anterior division			
Supramarginal Gyrus, posterior division			
Angular Gyrus		√	
Lateral Occipital Cortex, superior division		√	√
Lateral Occipital Cortex, inferior division		√	
Intracalcarine Cortex	√	√	√
Juxtapositional Lobule Cortex			
Paracingulate Gyrus			
Cingulate Gyrus, anterior division			
Cingulate Gyrus, posterior division		√	√
Precuneous Cortex		√	√
Cuneal Cortex		√	√
Frontal Orbital Cortex			
Parahippocampal Gyrus, posterior division		√	√
Lingual Gyrus	√	√	√
Temporal Fusiform Cortex, posterior division		√	√
Temporal Occipital Fusiform Cortex		√	√
Occipital Fusiform Gyrus	√	√	√
Frontal Operculum Cortex			
Central Opercular Cortex			
Parietal Operculum Cortex			
Supracalcarine Cortex		√	√
Occipital Pole	√	√	√
Middle Temporal Gyrus, temporooccipital part		√	

SUBCORTICAL			
**LABEL**	**FON1 > FON 2**	**FON 3 > FON 1**	**FON 3 > FON 2**
Left Cerebral White Matter		√	√
Left Cerebral Cortex		√	√
Left Lateral Ventrical			
Left Thalamus			
Left Caudate			
Left Putamen			
Left Pallidum			
Brain-Stem		√	
Right Cerebral White Matter	√	√	√
Right Cerebral Cortex	√	√	√
Right Lateral Ventricle			
Right Thalamus			
Right Caudate			
Right Putamen			
Right Pallidum			

References

Amaro, E. Jr., and Barker, G. J. (2006). Study design in fMRI: basic principles. Brain and Cognition 60(3), 220–232.

Avcıoğlu, D. (1978). Türklerin Tarihi. İstanbul: Tekin Yayınevi.

Brooks, S. J., Savov, V., Allzén, E., and Benedict, C. (2012). Exposure to subliminal arousing stimuli induces robust activation in the amygdala, hippocampus, anterior cingulate, insular cortex and primary visual cortex: a systematic meta-analysis of fMRI studies. NeuroImage 59, 2962–2973.

Casado-Aranda, L.-A., Dimoka, A., and Sánchez-Fernández, J. (2021). Looking at the brain: neural effects of "made in" labeling on product value and choice. Journal of Retailing and Consumer Services 60, 102452. doi: 10.1016/j.jretconser.2021.102452.

Çimen, S. (2021). Pazarlamada kültür kodlarının ve nörobilim deneylerinin kullanımı: bir ürünün bilinçdışı kültür kodunun keşfi ve nöropazarlama kapsamında fMRI tekniği ile test edilmesi. [doctoral dissertation]. Kocaeli (Türkiye): University of Kocaeli, Institute of Social Sciences, School of Management.

Durnez, J., Blair, R., and Poldrack, R. A. (2017). Neurodesign: optimal experimental designs for task fMRI. BioRxiv. Available at: https://doi.org/10.1101/119594.

Erbay, B. (2016). Göç ve müzik: blues ve arabesk üzerinde göçün etkileri. in Göç ve Kültür Sempozyum Bildirileri Kitabı Vol. 1, eds. N. Kahraman, Ç. Dürüst and T. Yılmaz (Amasya: Amasya Üniversitesi, KIBATEK Vakfı, KIBATEK), 355–362.

Erk, S., Spitzer, M., Wunderlich, A. P., Galley, L., and Walter, H. (2002). Cultural objects modulate reward circuitry. Neuroreport 13(18), 2499–2503.

Erkaya, H. (2016). Anadolu coğrafyasında göç olgusunun Selçuklu dönemi mimari yapılardaki tezyinata yansıması: Kayseri Güllük Camii mihrap örneği. in Göç ve Kültür Sempozyum Bildirileri Kitabı Vol. 1, eds. N. Kahraman, Ç. Dürüst and T. Yılmaz (Amasya: Amasya Üniversitesi, KIBATEK Vakfı, KIBATEK), 93–101.

Ford Otosan. (2015). Ford Cargo Rekabet Analizi 2004–2015. Ford Otosan.

Ford Otosan. (2015). Mercedes Trucks Rekabet Analizi 2004–2014. Ford Otosan.

Kafesoğlu, İ. (1977). Türk Milli Kültürü. İstanbul: Ötüken Neşriyat.

Kao, M.-H., and Mittelmann, H. D. (2014). A fast algorithm for constructing efficient event-related fMRI designs. Journal of Statistical Computation & Simulation 84(11), 2391–2407.

Kouider, S., and Dehaene, S. (2007). Levels of processing during non-conscious perception: critical review of visual masking. Philosophical Transactions of the Royal Society B Biological Sciences 362, 857–875.

Kümbetoğlu, B. (2008). Sosyolojide ve Antropolojide Niteliksel Yöntem ve Araştırma. İstanbul: Bağlam Yayıncılık.

Lindquist, M. A. (2008). The statistical analysis of fMRI data. Statistical Science 23(4), 439–464.

Liu, T. T. (2012). The development of event-related fMRI designs. NeuroImage 62(2), 1157–1162.

McClure, S. M., Li, J., Tomlin, D., Cypert, K. S., Montague, L. M., and Montague, P. R. (2004). Neural correlates of behavioral preference for culturally familiar drinks. Neuron 44, 379–387.

Meneguzzo, P., Tsakiris, M., Schioth, H. B., Stein, D. J., and Brooks, S. J. (2014). Subliminal versus supraliminal stimuli activate neural responses in anterior cingulate cortex, fusiform gyrus and insula: a meta-analysis of fMRI studies. BMC Psychology 2, 52.

NeuroPowerTools. (2020). Available at: http://www.neuropowertools.org/. [Accessed June 6, 2020].

Osmanoğlu, Ö. (2016). Türk sineması'nda dış göç olgusu: sosyo-kültürel karşılaşmalar, kimlik çatışması ve yabancılaşma. Marmara İletişim Dergisi 25, 77–98.

Ögel, B. (1984). İslamiyetten Önce Türk Kültür Tarihi. Ankara: T.C. Atatürk Kültür, Dil ve Tarih Yüksek Kurumu Türk Tarih Kurumu Yayınları.

Plassmann, H., Ramsøy, T. Z., and Milosavljevic, M. (2012). Branding the brain: a critical review and outlook. Journal of Consumer Psychology 22(1), 18–36.

Ran, G., Chen, X., Cao, X., and Zhang, Q. (2016). Prediction and unconscious attention operate synergistically to facilitate stimulus processing: an fMRI study. Consciousness and Cognition 44, 41–50.

Roullet, B., and Droulers, O. (2010). Neuromarketing, Le Marketing Revisité Par Les Neurosciences Du Consommateur. Paris: Dunod.

Roux, J. P. (2007). Türklerin Tarihi. trans. A. Kazancıgil and L. A. Özcan. İstanbul: Kabalcı Yayınevi.

Roxburgh, D. J. (2005). Turks, A Journey of Thousand Years, 600–1600. New York: Royal Academy Publications.

Ruch, S., Herbert, E., and Henke, K. (2017). Subliminally and supraliminally acquired long-term memories jointly bias delayed decisions. Frontiers in Psychology 8:1542.

Satık, G. (2018). Refik Halid Karay'ın "eskici'' adlı hikâyesinde göç izleği. in Uluslararası Avrasya Göç Sempozyumu Tam Metin Bildiri Kitabı, ed. H. Ateş (İstanbul: İstanbul Medeniyet Üniversitesi Medeniyet Araştırmaları Merkezi), 317–333.

Schaefer, M., Berens, H., Heinze, H.-J., and Rotte, M. (2006). Neural correlates of culturally familiar brands of car manufacturers. NeuroImage 31, 861–865.

Schaefer, M., and Rotte, M. (2006). Favorite brands as cultural objects modulate reward circuit. NeuroReport 18(2), 141–145.

Schaefer, M., and Rotte, M. (2007). Thinking on luxury or pragmatic brand products: brain responses to different categories of culturally based brands. Brain Research 1165, 98–104.

Schaefer, M., and Rotte, M. (2010). Combining a semantic differential with fMRI to investigate brands as cultural symbols. Social Cognitive and Affective Neuroscience 5(2-3), 274–281.

Sevim, Y. (2007). Göç ve Türk tiyatrosuna yansıması, iç/dış göç ve kültür. in IV. Kültür Araştırmaları Sempozyumu (İstanbul: Işık Üniversitesi), 108.

Stephan, K. M., Thaut, M. H., Wunderlich, G., Schicks, W., Tian, B., Tellmann, L. et al. (2002). Conscious and subconscious sensorimotor synchronization – prefrontal cortex and the influence of awareness. NeuroImage 15, 345–352.

Togan, A. Z. V. (1981). Umumi Türk Tarihine Giriş. İstanbul: İstanbul Üniversitesi Edebiyat Fakültesi Yayınları.

Türkan, H. K., and Arı, B. (2018). Karacaoğlan'da göç. Türük Uluslararası Dil, Edebiyat ve Halkbilimi Araştırmaları Dergisi 6(12), 263–282.

Üner, Ö. (2018). Sanat eserleriyle göçlere tanıklık. in Uluslararası Avrasya Göç Sempozyumu Tam Metin Bildiri Kitabı, ed. H. Ateş (İstanbul: İstanbul Medeniyet Üniversitesi Medeniyet Araştırmaları Merkezi), 346–375.

Vural, T. (2017). Türkülerdeki göç algısı. in Geçmişten Günümüze Göç III, ed. O. Köse (Samsun: Canik Belediyesi Kültür Yayınları), 2003–2008.

Wager, T. D., and Nichols, T. E. (2002). Optimization of experimental design in fMRI: a general framework using a genetic algorithm. NeuroImage 18(2), 293–309.
